# Polyoxometalates in environmental remediation and energy storage

**DOI:** 10.1039/d5en00964b

**Published:** 2026-02-02

**Authors:** Ingrid Gregorovic, Nahid Lotfian, Ruhollah Khajavian, Sukanya Maity, Masoud Mirzaei, Sib Sankar Mal, Manuel Aureliano, Annette Rompel

**Affiliations:** a Universität Wien, Fakultät für Chemie, Institut für Biophysikalische Chemie Josef-Holaubek-Platz 2 1090 Wien Austria annette.rompel@univie.ac.at https://www.bpc.univie.ac.at/en/; b Vienna Doctoral School in Chemistry (DoSChem), Universität Wien Währinger Straße 42 1090 Vienna Austria https://doschem.univie.ac.at/; c Department of Chemistry, Faculty of Science, Ferdowsi University of Mashhad Mashhad 9177948974 Iran mirzaeesh@um.ac.ir; d Department of Physics, Chemistry and Biology (IFM), Linköping University 58183 Linköping Sweden; e Department of Chemistry, National Institute of Technology Karnataka Surathkal Mangalore-575025 India malss@nitk.edu.in; f Faculdade de Ciências e Tecnologia (FCT), Campus de Gambelas, Universidade do Algarve 8005-139 Faro Portugal; g Centro de Ciências do Mar do Algarve (CCMAR/CIMAR LA), Campus de Gambelas, Universidade do Algarve 8005-139 Faro Portugal maalves@ualg.pt

## Abstract

Over recent decades, while environmental awareness and pollution control efforts have yielded localized improvements, ongoing industrial growth, rapid global population expansion, and escalating energy demands continue to drive significant global environmental pollution challenges. Polyoxometalates, a remarkable class of metal-oxide complexes, have recently emerged as promising compounds in the development of multifunctional materials for environmental pollutant removal, energy conversion and storage, and sensing. This review critically examines current research on their use for the removal of common toxic gases – such as H_2_S, NO_*x*_, and volatile organic compounds (VOCs) – from polluted air, as well as the elimination of various organic dyes, heavy metals, and pharmaceutical contaminants from wastewater. POMs have also gained recognition as adaptable redox-active materials suitable for next-generation energy storage systems. Their high electron-transfer capacity, structural flexibility, and remarkable chemical stability make them ideal candidates for various applications. POMs can facilitate multi-electron redox processes, allowing for their application in batteries, supercapacitors, and hybrid devices, which results in improved energy density and cycling performance. Recent developments in POM-based composites and electrode designs are further discussed for innovative, sustainable, and scalable energy storage solutions. Additionally, their tunable electrical and magnetic properties make them effective sensors for detecting various environmental pollutants.

## Abstract 2

The article is about the application of POMs and POM-based hybrid materials in the removal of environmental pollutants and energy storage devices.

## Graphical abstract

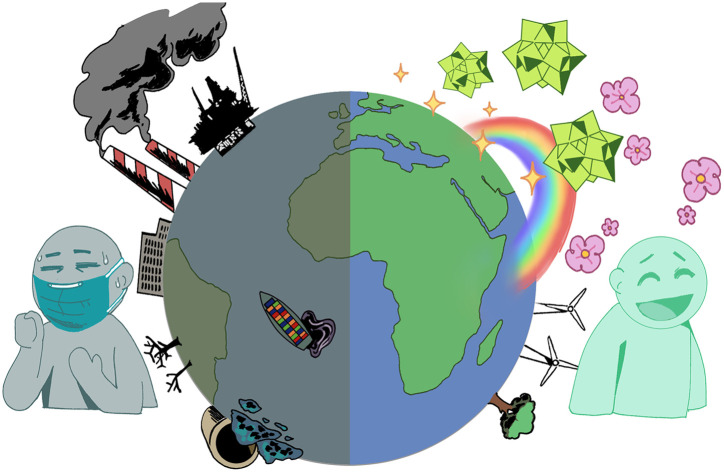

**Environmental significance**

This comprehensive review covers remediation, sensing, and energy storage, inspiring sustainable polyoxometalate innovations. Polyoxometalates (POMs) are metal-oxide complexes with exceptional redox tunability, pseudocapacitive charge storage, and great structural versatility, making them ideal nanomaterials for environmental remediation. This review analyses the POM-based technologies for detection and treatment of air/water pollutants, surpassing conventional technologies that require harsh conditions for hard-to-remove contaminants such as refractory sulfur compounds. Global pollution includes refractory sulfur compounds from fossil fuels, toxic gases in air, and heavy metals, dyes, and emerging contaminants in water, driving acid rain, smog, antibiotic resistance and ecosystem toxicity. POMs provide efficient oxidative desulfurization, photocatalytic dye/heavy metal removal, and multipollutant adsorption in POM-based hybrid materials. POM structures enable visible-light mineralization in low-input environments with less energy; benefits include scalable low-toxicity remediation, while metal leaching risks under extreme pH are mitigated by heterogenization.

## 1 Introduction

In recent times, rapid industrial and technological development has caused a significant increase in energy demand and environmental pollution (EP).^1–3^ The Encyclopaedia Britannica defines environmental pollution as the addition of any substance (solid, liquid, or gas) or any form of energy (such as heat, sound, or radioactivity) to the environment at a rate faster than it can be removed from the environment or stored in a harmless form. It further categorizes environmental pollution based on the affected medium into air, water, and land pollution.^4^ Increasing attention has been paid to the development of new methods for the removal of potential environmental pollutants^1–3^ during industrial processes and clean energy production.^5^ Although industrial development has brought many positive aspects to everyday life (*e.g.* new technology, better food safety and supply, medicines, *etc.*), it has also increased consumption and pollution of natural resources (water, soil and air),^3,6^ which has become both an environmental problem and a health threat for the entire human population.^7^

**Fig. 1:**
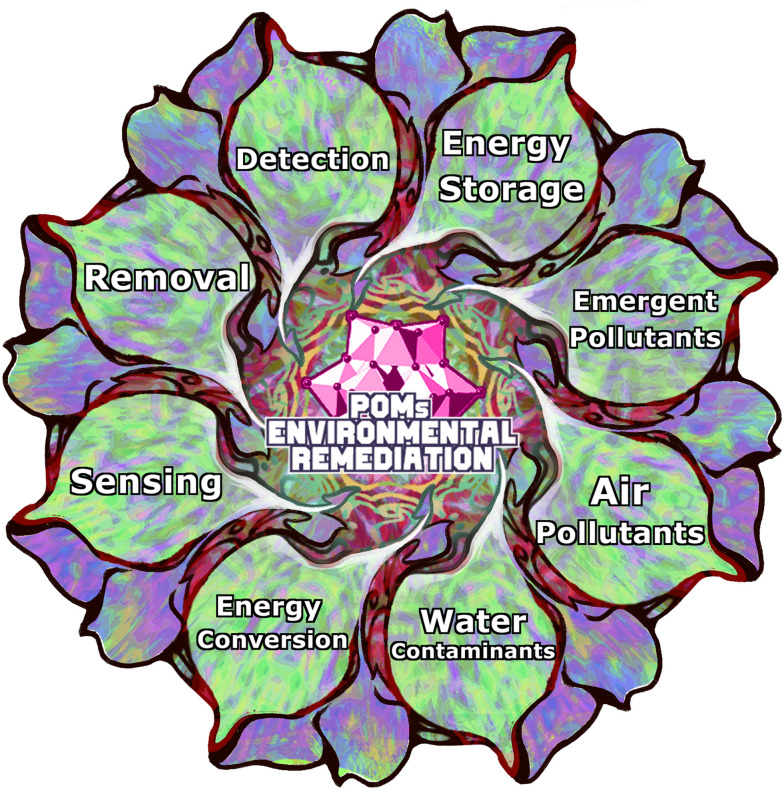
Schematic illustration of the main roles of polyoxometalates (POMs) in environmental remediation and energy storage. The central part emphasizes POM-based environmental remediation, while the surrounding segments shows key applications, including pollutants detection, removal, sensing, treatment of (health) emerging pollutants, air pollutants and water contaminants, energy storage, energy conversion, and signalling.

The global shortage of clean water and the pollution of water resources pose critical health, economic,^8^ and environmental challenges.^9–11^ Especially in many underdeveloped and currently developing parts of the world, sewage wastewater and wastewater from different factories are discharged directly into the environment, causing catastrophic water pollution (section 2; Fig. 1) with hard-to-remove toxic chemicals – inorganic pollutants (section 2.2) such as heavy metals (section 2.2.1) and organic pollutants^12^ (section 2.3) such as organic dyes^13^ and solvents.^14^

The most prominent classes of health emerging pollutants (EPs)^15–17^ (section 3; Fig. 1) are pharmaceuticals^18^ (section 3.1), pesticides and herbicides^19^ (section 3.2), cosmetics,^20,21^ industrial and household products,^22^ metals^13^ (section 2.2), dyes^13^ and aromatic hydrocarbons (section 2.3.2).^23^ The presence of EPs in wastewater has been associated with the development of bacterial resistance,^20,24^ and mutagenicity and toxicity in aquatic organisms^21,25^ and humans.^22,25^

Pesticides and herbicides (for their removal, see section 3.2) are an inevitable part of the modern agricultural industry and food production.^26^ However, in addition to ensuring yields and protecting crops from pests, the widespread use of these chemicals also affects soil enzymes and microorganisms^26^ crucial for many essential biological processes, such as N_2_-fixation in plants by rhizobacteria.^27^ The excessive use of pesticides also impacts wildlife, with a scientific focus on bees, birds, fish and small mammals.^28–30^ Human health is also affected by pesticide residues in the environment and food^31^ causing various health problems.^32–37^ Therefore, many Western countries (*e.g.* EU, USA) have introduced stricter controls and limitations^38^ on the use and allowable levels of pesticide residues in food, water and soil.^31^

Fossil fuels continue to be one of the primary energy sources in today's world.^39^ Their combustion (section 4.1) produces various toxic refractory sulfur-containing compounds (dibenzothiophenes, DBTs)^40,41^ and gases such as hydrogen sulfide^42^ (section 4.2.1), nitrogen oxides (section 4.2.2), and sulfur oxides (section 4.2.2),^43^ which cause different severe environmental issues such as global warming,^40^ smog^44^ and acid rains.^45^ Toxic gases generated from traffic and flue gases from the industry have made poor air quality an important factor in causing respiratory^46–48^ and cardiovascular health^49^ issues in urban areas.^50^ Air purification (section 4; Fig. 1) using adsorption processes^51^ (section 4.2) and desulfurization of fossil fuels^52^ (section 4.1) is currently a logical approach to decreasing air pollution.

Global environmental pollution has escalated to crisis levels, driven significantly by fossil fuel combustion that releases refractory sulfur compounds such as dibenzothiophenes (DBTs), toxic gases including H_2_S, NO_*x*_, and SO_2_, and emerging contaminants resistant to conventional treatment methods. These pollutants contribute directly to the formation of acid rain, smog, and severe health crises that impact billions worldwide.^52,53–59^ Conventional technologies like hydrodesulfurization (HDS) are ineffective against sterically hindered DBTs and require extreme conditions (300–400 °C, 30–100 bar H_2_), while amine scrubbing and selective catalytic reduction (SCR) systems^60^ face limitations in capacity, cost-efficiency, and simultaneous multi-pollutant management for air purification.^53,61^ Water faces persistent heavy metals, dyes, pharmaceuticals, and microplastics that evade standard filtration and oxidation.^15–17,20,22,24,25^ POMs offer a powerful, direct solution to these multifaceted challenges *via* mild-condition oxidative desulfurization achieving over 99% removal of refractory sulfur, versatile multi-pollutant adsorption and catalysis, and photocatalytic mineralization.^62,63^ Their uniquely tunable redox properties and acidity provide sustainable remediation options precisely where traditional technologies are insufficient.^53,62^

The first step in combating pollution is building a good system to monitor and detect various harmful compounds present in the environment. In this regard, various materials have been extensively researched and designed to develop new chemical,^64^ electrochemical,^65^ and biosensors^66^ (section 5; Fig. 1) for environmental monitoring. For example, metal or metal oxide nanoparticles are widely used to develop various electrochemical sensors.^67–69^

New efficient technologies for energy conversion and storage need to be developed (section 6; Fig. 1) because renewable energy sources such as wind, hydroelectric, and solar power alone cannot meet the world's current energy demands.^70^ In addition, the growing popularity and use of various portable electronic devices in everyday life have led to intensive research and development of new efficient battery technologies such as lithium-ion,^71^ sodium-ion,^72^ and redox-flow batteries.^73^ Rechargeable Li-ion batteries and supercapacitors have been commercially utilized due to their ability to hold high energy with power density for various applications (*e.g.*, electric vehicles, power tools, or portable/wearable electronic devices).^74–76^

### 1.1 Polyoxometalates

Polyoxometalates (POMs)^77^ are a class of transition metal-oxide clusters, usually containing Mo or W ions in their highest oxidation states. They exhibit exciting and unique physical and chemical properties, such as controllable shape and size,^77^ oxo-enriched surfaces, photoactivity,^78^ molecular conductivity,^79^ excellent chemical stability, and redox properties.^80^ These properties have led to their increasing use in diverse fields, including catalysis,^81,82^ magnetism,^83^ medicine,^84,85^ biotechnology,^86^ protein crystallography,^87–89^ and material science.^90^

**Fig. 2:**
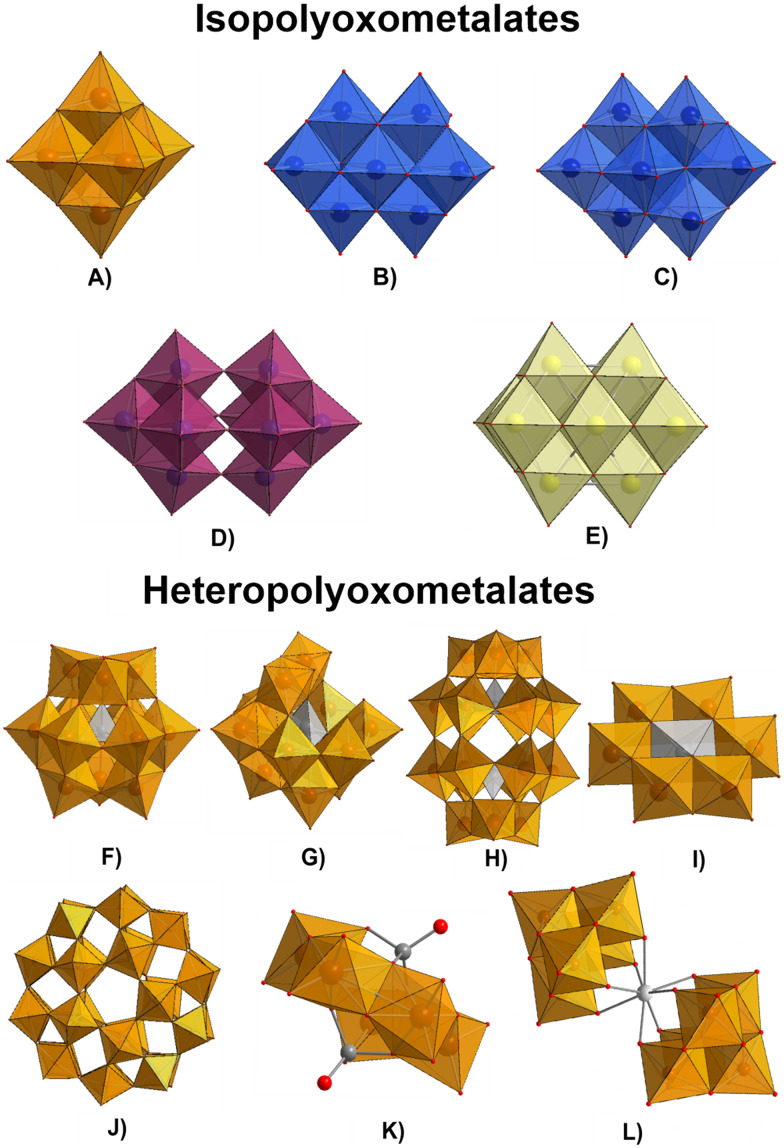
Structures of isopolyoxometalates and heteropolyoxometalates: A) Lindqvist ([M^VI^_6_O_19_]^2−^), B) heptamolybdate ([Mo^VI^_7_O_24_]^6−^), C) β-octamolybdate (β-[Mo^VI^_8_O_26_]^4−^), D) decatungstate ([W^VI^_10_O_32_]^4−^), E) decavanadate ([V^V^_10_O_28_]^5−^), F) Keggin ([XM^VI^_12_O_40_]^*n*−^), G) monolacunary Keggin ([XM^VI^_11_O_39_]^*n*−^), H) Wells–Dawson ([XM^VI^_18_O_62_]^*n*−^), I) Anderson–Evans ([XM^VI^_6_O_24_]^*n*−^), J) Preyssler ([MP_5_M^VI^_30_O_110_]^(15−*n*)−^), K) Strandberg ([X_2_Mo^VI^_5_O_23_]^*n*−^), and L) Weakley ([M^III^(M^VI^_5_O_18_)_2_]^*n*−^). Color legend: orange = M (either Mo^VI^, W^VI^ or V^V^), blue = Mo^VI^, purple = W^VI^, yellow = V^V^, gray = X (heteroion), white = M^III^, and red = oxygen.

POMs are typically synthesized *via* controlled acidification and condensation of simple metal oxoanions such as Mo^VI^O_4_^2−^, W^VI^O_4_^2−^, or V^V^O_4_^3−^, which allows the precise formation of diverse structural archetypes, including some of the most common POM archetypes like Keggin (Fig. 2F), Wells–Dawson (Fig. 2H), and Anderson–Evans (Fig. 2I).^76,77,91–93^ Their functionality in pollutant removal is often enhanced by immobilization or hybridization,^94^ where POMs are incorporated into different solid supports like metal–organic frameworks (MOFs),^95–105^ porous silica,^106,107^ graphene oxide (GO_*x*_),^108,109^ or polymeric supports,^94,110,111^ improving POM stability and catalytic efficiency.^94,101,112^ Ion exchange with organic or inorganic cations,^113–115^ surface modifications,^94^ or doping with lanthanide ions^116^ further tailor their physicochemical properties. Such synthetic versatility enables customization of POM-based materials to optimize catalytic, adsorptive, and photocatalytic performance in environmental remediation.^94,101,117^ The structural characteristics of polyoxometalates can be divided into two main general subgroups, isopolyoxometalates and heteropolyoxometalates.^76,77^ The isopolyoxometalates, with the general formula [M_*x*_O_*y*_]^*n*−^ (where M = Mo, W or V; Fig. 2A–D), contain only addenda metals and oxygen atoms in their structure, such as Lindqvist^118,119^ ([M_6_O_19_]^2−^; Fig. 2A), heptamolybdate^120,121^ ([Mo^VI^_7_O_24_]^6−^; Fig. 2B), octamolybdate^122,123^ ([Mo^VI^_8_O_26_]^4−^; Fig. 2C), decatungstate^124,125^ ([W^VI^_10_O_32_]^4−^; Fig. 2D) and decavanadate^126,127^ ([V^V^_10_O_28_]^6−^; Fig. 2E). Heteropolyoxo species have the general formula [X_*z*_M_*x*_O_*y*_]^*n*−^ (X = heteroion, M = Mo, W or V, *z* < *x*, *y* = number of oxygen atoms in the POM structure, *n* = overall anion charge), where different heteroions X are present alongside addenda ions M and oxygen atoms. This composition allows them to form a variety of structural types, including common ones such as Keggin^128,129^ ([XM_12_O_40_]^*n*−^; Fig. 2F), lacunary Keggin^130^ ([XM_11_O_39_]^*n*−^; Fig. 2G), Wells–Dawson^131,132^ ([X_2_M_18_O_62_]^*n*−^; Fig. 2H), Anderson–Evans^133,134^ ([XM_6_O_24_]^*n*−^; Fig. 2I), Preyssler^135^ ([MP_5_M_30_O_110_]^(15−*n*)−^; Fig. 2J), Strandberg^136,137^ ([X_2_Mo^VI^_5_O_23_]^*n*−^, (X = P^V^, S^VI^, As^V^, Se^VI^); Fig. 2K), Weakley^138,139^ ([M^III^(M^VI^_5_O_18_)_2_]^*n*−^; Fig. 2L), among others. Moreover, if the POM solution is reduced, a unique class of giant molybdenum blue and molybdenum brown-type structures ({Mo_154_} and {Mo_132_}) are formed.^140^ For more detailed information on POMs structures and general synthetic procedures, the reader is referred to the reviews in ref. 91–93, 141 and 142.

Pure POMs exhibit different solution behaviors across the wide pH range; some, like Wells–Dawson-type structures, maintain their structural integrity, while others, such as Keggin-type POMs, undergo monolacunarization under acidic conditions relevant to environmental remediation.^143–145^ Their high solubility in aqueous media presents significant challenges for their use in applications, including leaching during wastewater treatment and difficulties in catalyst recovery.^113^ While pure POMs often dissolve in aqueous media,^113^ strategic heterogenization approaches,^94^ such as immobilization on mesoporous silica (SBA-15),^106,107,146^ metal–organic frameworks (like UiO-66 and MIL-101),^95–101^ and POM-supported ionic liquid phases (POM-SILPs),^112,147^ address this issue. Such methods significantly reduce leaching to <1% after 10 cycles (Tables S1 and S2). These enable recyclability over 5–10 cycles with minimal activity loss (Table S1).^148–151^ Nevertheless, challenges remain including potential metal cation leaching from POM-composites under prolonged extreme pH exposure and the need for long-term stability studies under real environmental conditions. These heterogenized systems demonstrate >95% POM retention after multiple uses.^63^

Keggin-type POMs (Fig. 2F and G) are the most widely studied POM archetype, representing an average of 77.6% of all published articles, particularly in applications targeting environmental pollutant removal (approximately 69%). This predominance in environmental applications surpasses that of Wells–Dawson (Fig. 2H; ∼9%), Anderson–Evans (Fig. 2I; ∼9%), sandwich-type (Fig. 2L; ∼5%), isopolymolybdates (Fig. 2A–E; ∼5%), and other types of POMs (each ∼5%). In this review, Keggin-type POMs (Fig. 2F and G) are most frequently addressed in section 3 (wastewater treatment, 75%) and section 4 (air pollutant removal, 85%). Wells–Dawson type POMs (Fig. 2H) rank second in environmental pollutant removal (average 16.9%), with their primary use found in sensing (75%, section 5). Notably, section 4.1 showcases the broadest diversity of structural archetypes for POM-mediated fossil fuel desulfurization.

**Fig. 3:**
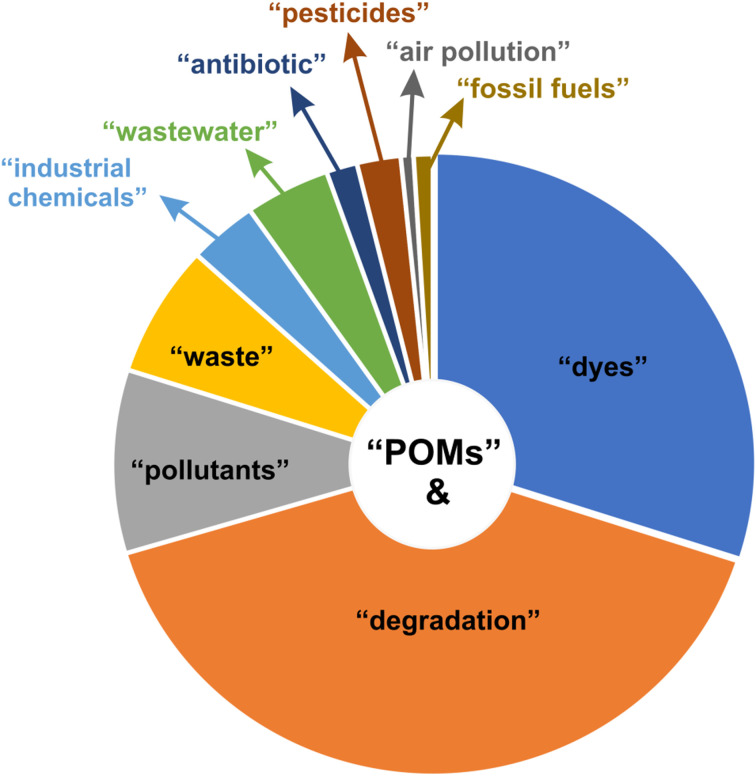
Number of articles containing the term “polyoxometalate” combined with keywords such as dyes, pollutants, industrial chemicals, wastewater, pesticides, and antibiotics, as of August 14, 2025.

A literature search conducted on Web of Science in August 2025 (Fig. 3) revealed that approximately 12% (1928) of the published articles on POMs related to the keyword “environment”, out of a total of 15 830 articles. As of August 14, 2025, the number of articles varies by specific subject: the combination of “polyoxometalate” and “degradation” yielded 1306 articles, while “polyoxometalates” and “dyes” yielded 910 articles. These numbers exceed those for “polyoxometalate” combined with “pollutants” (353), “waste” (258), “industrial chemicals” (134), and “wastewater” (215). Fewer articles were found for combinations with “antibiotics” (98), “pesticides” (48), “fossil fuels” (40), and “air pollution” (26). The number of publications related to “antibiotics” and “wastewater” has more than doubled over the past 2 years, reflecting a marked increase in research interest in these areas.

In fact, the importance of POMs in environmental science and their relationship to sustainable development and green chemistry is clearly increasing. POMs are crucial in environmental science for their roles as catalysts and adsorbents, aiding in the degradation of emerging pollutants such as dyes, plastics, and antibiotics, in addition to well-known organic and inorganic contaminants.^152–160^ Moreover, POMs can act as novel antibacterial agents for water purification.^161^ As described in the sections below, POMs are also fundamental for sustainable development by enabling energy applications such as solar hydrogen production and energy storage.^70–76^ Recent studies further explore POMs as electrochemical sensors for the simultaneous detection of inorganic heavy metal ions and organic antibiotic contaminants in aquatic environments,^162^ and as triboelectric nanomaterials for gait monitoring.^163^

## 2 Water decontamination by polyoxometalates

Inorganic contaminants (section 2.2) enter the environment as inorganic salts, mineral acids, sulfates, cyanides, and metal ions, including heavy and radioactive metals. These contaminants are generally more persistent and more difficult to eliminate than organic ones.^164,165^ On the other hand, organic contaminants (section 2.3) represent a more diverse class, consisting of organic dyes, aromatic hydrocarbons, pesticides, and pharmaceuticals (see section 3 for pharmaceutical and pesticide removal). Due to rapid industrial development, large amounts of industrial, sewage, and agricultural waste discharged into water bodies cause organic pollutants to become pseudo-persistent in the ecosystem.^166,167^ Therefore, the removal of this class of contaminants requires careful consideration to move toward a sustainable ecosystem.

As discussed in section 2.1, oxidation, catalysis, photocatalysis, ion-exchange, adsorption, and membranes are among the commonly used technologies for the removal of these pollutants due to their high efficiency, cleanliness, and simple operation. POMs have shown promise in mitigating the global water purification issue using the above-mentioned technologies. This section covers novel solutions by highlighting recent achievements in designing multi-component materials for use in water-purification systems.

### 2.1 Emerging pollution treatment technologies

**Fig. 4:**
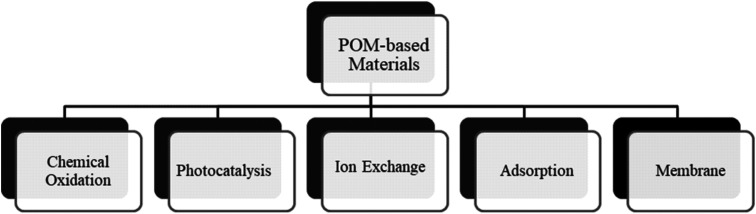
Summary of tertiary treatment technologies used against inorganic and organic pollutants for water purification.

Water treatment is a multi-stage process, comprising several stages with various technologies. Tertiary treatment is the final stage of the multi-stage wastewater treatment process. It is used after preliminary stages, and commonly used techniques utilized for the treatment include oxidation, photocatalysis, ion exchange, adsorption, and membranes technology (Fig. 4).^168^

Chemical oxidation is a cost-effective and simple technology for the decontamination of both organic and inorganic pollutants using an oxidizing agent such as chlorine, hydrogen peroxide, ozone, and molecular oxygen. In advanced oxidation processes (AOPs), POMs (especially iron-containing POMs) have been used as efficient catalysts for the decomposition of the oxidizing agent (H_2_O_2_) and removal of organic pollutants.^169^ In particular, POMs can initiate the activation through electron transfer to H_2_O_2_ (originating from the redox property of the addenda atoms) or *via* formation of peroxo complexes.^147,170^ This method, however, may produce secondary pollutants that are formed after the initial oxidation. This may cause a decrease in the catalyst selectivity, while increasing the costs.

In photocatalysis, the ability of the catalyst to harvest photons from a light source and to generate free radicals to undergo photocatalytic oxidation or reduction reactions is crucial. In this regard, POMs have shown promise since i) their band gap value can be adjusted by changing the heteroatoms or adjusting the valence states of addenda atoms, and ii) they can store multiple electrons in one molecule; thus they exhibit fast charge transfer properties.^171,172^ Due to some drawbacks associated with pure POMs (*e.g.*, limited light absorption, high solubility), they are often employed in the form of hybrids or composites.^112^ In these structures, the intermolecular interactions between two species can improve the stability and promote the lifetime of photogenerated charge carriers. In this regard, the incorporation of noble metals,^173,174^ metals from the lanthanide series,^175^ metal oxides,^176^ metal–organic frameworks^177^ and metal-free species^110^ have been reported to be effective. Ion exchange water purification technology relies on the availability of exchange surfaces with accessible specific surface area and the ability to reversibly uptake/release ions from water. POMs can fulfill some of these requirements. For example, their diverse topology, high negative charge, and redox properties of POMs have turned them into potential candidates for cation (heavy metal) uptake and exchange. However, POMs lack a high surface area that is problematic.^113^

Adsorption-based protocols have been extensively used for wastewater treatment on the account of cost, simplicity, and energy considerations. The concept of this approach is based on removing pollutants by promoting their adsorption on the adsorbent surface *via* physical or chemical interactions.^178^ In this context, some intrinsic properties of POMs (*e.g.*, high negative charge, strongly basic oxygen surfaces) are advantageous for the physi/chemisorption of adsorbate molecules. However, when considering POMs as water purifiers, some limitations such as their high solubility and the low surface area must be taken into consideration. The heterogenization of POMs by inorganic substrates^106,107,179^ or organic matrices^111,179,180^ is the common approach to solve their solubility issue and low specific surface area. In heterogenization with organic matrices, the surface chemistry of the matrix plays an important role. Along with the degree of POM dispersion and matrix morphology, it can enhance the physicochemical properties and improve the membrane's performance. Heterogenization by porous coordination polymers (MOFs) is another successful strategy that combines both the merits of POMs and MOFs (*e.g.* recyclability and porosity).^95–101^ This strategy is commonly used for the adsorptive removal of cationic dyes.^102–105^ However, the catalytic activity of POM composites greatly depends on their structural properties. In some cases, as for POM@MOF composites, the activity is mainly governed by pore-dependent diffusion limitation, where the match of pore aperture and POM diameter is essential.^139^ Meanwhile, each individual structural component can also induce different electron transfer kinetics due to its unique electron-storage/transfer capacity.^181,182^

Controlled deposition of POMs on substrates is another concept that enables the fabrication of POM-based functional devices for water purification.^94^ Techniques such as layer-by-layer assembly, casting, and dip-coating have been recently reported.^183–185^

**Fig. 5:**
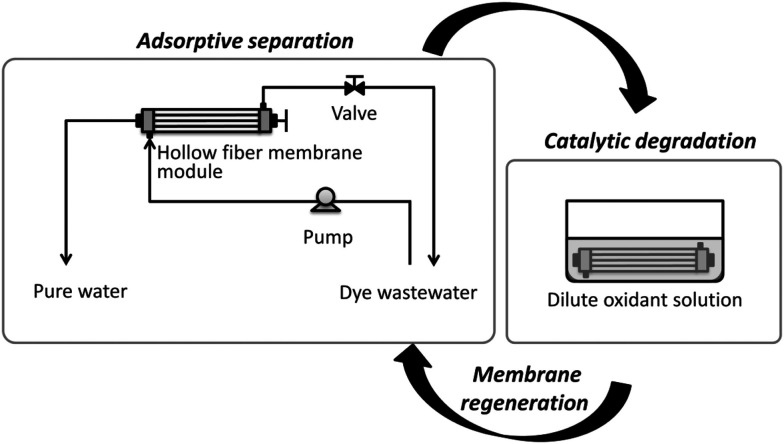
Illustration of a POM-integrated catalytic membrane for organic dye decontamination from water. Reproduced from ref. 190 with permission from Elsevier, copyright 2016.

Membrane filtration is a reliable, and environmentally friendly process with relatively low cost and simple operation, which has been widely used for water purification. Catalytic membranes represent a new generation of membranes created by incorporating inorganic particles, such as POMs, into a polymer matrix to enhance the membrane's (photo)catalytic properties.^186–189^ As a convincing demonstration of this approach, Yao *et al.* designed and fabricated an amine-functionalized APTMS-treated PEI membrane for dye removal from wastewater. [PV^V^_2_Mo^VI^_12_O_40_]^5−^ was incorporated into the matrix *via* a simple sol–gel protocol. The presence of [PV^V^_2_Mo^VI^_12_O_40_]^5−^ in the membrane not only enhanced the mechanical strength of PEI but also catalyzed the degradation of RB5 in the presence of a diluted solution of an oxidant (Fig. 5).^190^ The presence of different POM species was reported to be necessary for the self-cleaning property of the membrane.^183^

### 2.2 Removal of inorganic pollutants

#### 2.2.1 Removal of heavy metals

Catalysis and photocatalysis are appropriate strategies for removing reductive toxic metal ions from water.^108,191,192^ Gong *et al.* demonstrated that different highly reduced molybdophosphate hybrid materials such as {Co^II^[P^V^_4_Mo^V^_6_X_31_]_2_}^*n*−^ (X = O or OH)^193^ or {Mn^II^[P^V^_4_Mo^V^_6_O_31_]_2_}^194^ clusters could act as efficient heterogeneous catalysts for the reduction of toxic Cr(vi) to nontoxic Cr(iii) in the presence of formic acid as the reducing agent under mild conditions. These noble metal-free POM catalysts have great potential to replace high-priced Pt/Pd catalysts for the elimination of Cr(vi) from water.

POMs or their modified derivatives, acting as electron reservoirs, have demonstrated efficiency in photoactivity, especially in visible light photocatalysis. Therefore, there is continuous effort to design a POM-based photocatalyst that can utilize solar energy for the reduction of highly toxic Cr(vi). Due to the good photocatalytic response of Ag-based photocatalysts, Wang's group heterogenized H_3_[PMo^VI^_12_O_40_] with Ag^+^ counter cations.^195^ Ag/Ag_*x*_H_3−*x*_PMo^VI^_12_O_40_ nanowires were synthesized by a facile solid-state reaction route and *in situ* photodeposited method. The resulting Ag/Ag_*x*_[H_3−*x*_PMo^VI^_12_O_40_] (Ag/AgHPMo_12_) nanowires, where *x* denotes the irradiation time (*x* = 2, 4, 6, 8 h, respectively), showed higher stability and photocatalytic activity than traditional Ag-based photocatalysts (*e.g.* Ag/AgX (X = Cl, Br, I), AgPO_4_ or AgVO_3_)^196–199^ for Cr(vi) reduction. This is attributed to their good visible-light absorption and reversible redox properties of the Keggin-type POM (Fig. 2F). In addition, a part of the Ag^+^ in the nanowires was *in situ* photoreduced to Ag NPs under visible light irradiation, and these Ag NPs enhanced visible-light absorption and the charge separation of photogenerated electrons (e^−^) and holes (h^+^) in Ag/[AgHPMo^VI^_12_]. In order to improve the catalytic efficiency of Ag/[Ag_*x*_H_3−*x*_PMo^VI^_12_O_40_] nanowires, these Ag-loaded 1D silver POM nanowires were well dispersed on duplicated 2D graphite-like carbon nitride (g-C_3_N_4_) nanosheets.^200^ The obtained [Ag_*x*_H_3−*x*_PMo^VI^_12_O_40_]/Ag/g-C_3_N_4_ (*x* represents the irradiation time; *x* = 2, 4, and 6 h, respectively) 1D/2D Z-scheme heterojunction photocatalyst exhibited excellent and durable photocatalytic performance towards the reduction of Cr(vi), methyl orange (MO) and tetracycline (TCY) under visible light.^200^

**Fig. 6:**
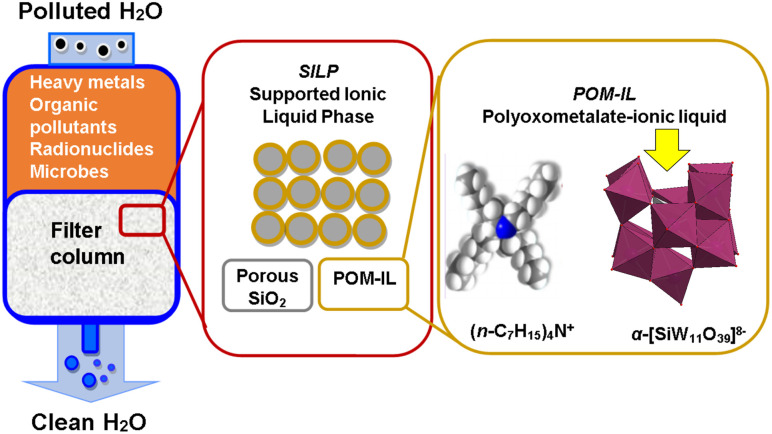
Water purification using POM-SLIPs: the POM-SLIP column filter removes toxic heavy metals (*e.g.* Ni(ii), Pb(ii), UO_2_(ii)), microbes (*E. coli*), and aromatic organic pollutants (*e.g.* trityl dyes) due to the presence of lacunary polyoxometalate anions with specific metal-binding sites (yellow arrow) and antimicrobial tetra-alkyl ammonium cations. Reproduced from ref. 63 with permission form Wiley-VCH, copyright 2017.

In attempts to obtain efficient photocatalysts based on inorganic–organic hybrid POMs, a series of [Ag_4_(H_2_O)(L)_3_(SiW^VI^_12_O_40_)], [Zn(L)(H_2_O)]_2_[SiW^VI^_12_O_40_]·3H_2_O, [Cu(L)(H_2_O)]_2_[SiW^VI^_12_O_40_], and [Cu_2_(L)_2_(HPW^VI^_10_W^V^_2_O_40_)]·4H_2_O (L = 1,4-bis(3-(2-pyridyl)pyrazole)butane), have been synthesized.^201^ Interestingly, [Ag_4_(H_2_O)(L)_3_(SiW^VI^_12_O_40_)] (1) hybrid was able to act as an efficient photocatalyst to reduce Cr(vi) using the scavenger isopropanol under visible light at ambient temperature. In comparison with [Ag_4_(H_2_O)(L)_3_(SiW^VI^_12_O_40_)], the three other POM hybrids showed relatively weak photocatalytic activity. In a possible reduction mechanism of Cr(vi) to Cr(iii), first, the [Ag_4_(H_2_O)(L)_3_]^4+^ unit was excited under visible light, and the excited state electrons on the organic ligand were inclined to transfer to the [Ag_4_(H_2_O)(L)_3_(SiW^VI^_12_O_40_)] POM. Simultaneously, the isopropanol on the surface of the hybrid yielded reducing radicals and captured the photoinduced holes produced by the hybrid photocatalyst. Finally, the isopropanol scavenged the photoinduced holes and formed CO_2_, H_2_O, and other products. This charge transfer maintains the recombination of holes and electrons. The electrons accumulated on [Ag_4_(H_2_O)(L)_3_(SiW^VI^_12_O_40_)] were responsible for reducing Cr(vi). It was concluded that the much larger [Ag_4_(H_2_O)(L)_3_]^4+^ metal–organic unit, in comparison to the other metal–organic units presented in other above-mentioned inorganic–organic hybrids, is probably responsible for the higher photocatalytic activity of the [Ag_4_(H_2_O)(L)_3_(SiW^VI^_12_O_40_)] compared to the other three compounds.^201^ Adsorption is the other most used purification technique to remove heavy metals from wastewater. In order to prepare a multi-functional composite, Herrmann *et al.*^63^ used a combination of lacunary Keggin anions [α-SiW^VI^_11_O_39_]^8−^ and tetra-*n*-alkyl ammonium cations ((*n*-C_6_H_13_)_4_N^+^ and (*n*-C_7_H_15_)_4_N^+^) to prepare a highly viscous, lipophilic POM-IL complex, which was then immobilized on porous silica to give POM-SILP.^63^ Each component of the POM-SILP composite contributed to the removal of a specific type of water contaminant. The lacunary Keggin tungstate anions (Fig. 2G) were responsible for metal–ion binding, whereas the long-chain quaternary organo-ammonium cations^202^ acted as an antimicrobial. In addition, the POM-IL lipophilicity enabled the adsorption of organic contaminants, and the silica support bound radionuclides. Thus, using the water-insoluble POM-SILP composite in filtration columns allowed the simultaneous removal of toxic heavy metals (as Ni^2+^, Pb^2+^, Cu^2+^, Cr^3+^ and Co^2+^), microbes (*E. coli*), organic aromatics (trityl dye), and nuclear waste (UO_2_^+^) from water (Fig. 6).^63^

**Fig. 7:**
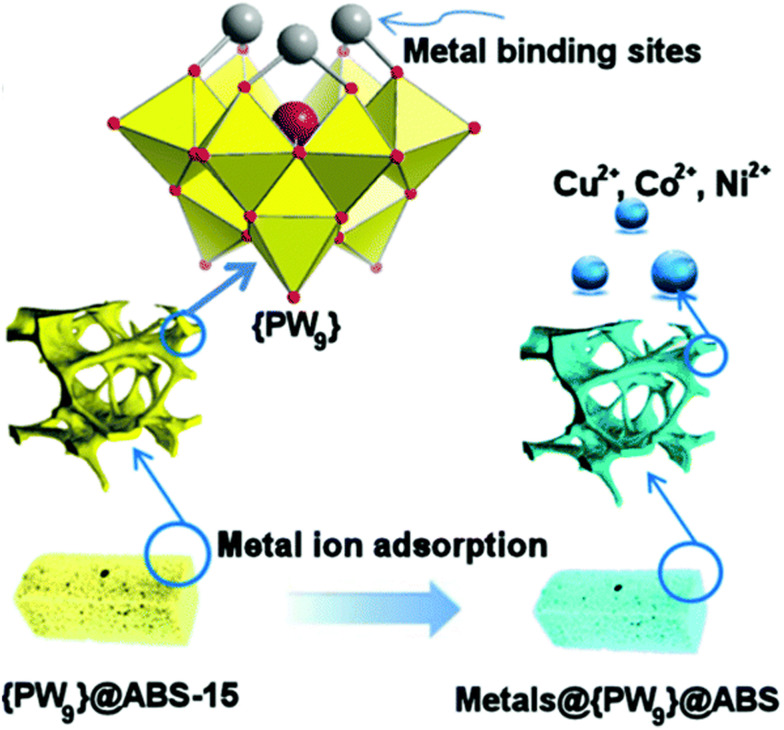
Schematic illustration of the POM-modified 3D-printed copolymer substrates used for transition-metal removal by the cation binding sites of the lacunary [α-PW^VI^_9_O_34_]^9−^. Reproduced from ref. 205 with permission from The Royal Society of Chemistry, copyright 2018.

The highly hydrophobic nature of POM-IL leads to surface heterogeneity and thus facilitates biphasic removal of metal ions from aqueous solutions. At the same time, the negative charge present on the POM units is the driving force for the removal of metal ions with a positive charge. In order to increase the removal of heavy metals from water by POM-IL, Shakeela and Rao synthesized a series of Keggin-based ionic liquids by reacting *in situ* generated first-row transition-metal ion (Mn^2+^, Fe^3+^, Co^2+^, Ni^2+^, Cu^2+^, and Zn^2+^) substituted monolacunary Keggin with tetraoctylammonium (TOA) cations.^203^ Metal-substituted lacunary POMs carried a relatively higher negative charge which facilitated the absorption of metal cations. Thus, all these thermoreversible POM-ILs effectively removed Cd^2+^ and Pb^2+^ metal ions from the aqueous phase.^203^ Embedding POM-ILs with tri-lacunary Keggin [α-PW^VI^_9_O_34_]^9−^ featuring coordinative binding of up to six metal cations into 3D printed organic polymers^204^ has been shown to produce a highly porous organic–inorganic composite for effective transition-metal removal (Fig. 7).^205^

Cation exchange is another process for the removal of various metal cations from water. Synthetic inorganic ion exchangers with well-defined chemical and phase compositions appear to be the most suitable ones compared to organic ion exchangers due to higher thermal and chemical stability and higher exchange capacity and selectivity for a wide range of metal ions.^113^ For example, Cronin's group designed an inorganic open framework nanocube-based K_18_Li_6_[Mn^II^_8_(H_2_O)_48_P_8_W^VI^_48_O_184_]·108H_2_O, from highly anionic crown-type POM ([P_8_W^VI^_48_O_184_]^40−^) and Mn^II^ as linkers to accommodate Cu^II^ cations from a solution into the network of channels and cavities. The cation-exchange capacity and rate are controlled by oxidizing the Mn linkers from +II to +III.^206^ In some cases, POM-IL systems exhibited greater efficiency than conventional ion-exchange resins.^207^

#### 2.2.2 Removal of radioactive metals

Although metal–organic frameworks (MOFs) initially exhibited a unique performance for the adsorptive removal of metal ions, most of these materials have low stability in aquatic media, which has limited their applications for water purification. To improve the stability of MOFs, Zou *et al.* functionalized HKUST-1 MOF with Keggin-type POM [H_3_PW^VI^_12_O_40_] POM (Fig. 2F) to form HKUST-1@[H_3_PW^VI^_12_O_40_] under microwave conditions. It was proposed that the improved water stability of HKUST-1@[H_3_PW^VI^_12_O_40_] was the result of POMs being encapsulated into HKUST-1 pores. The HKUST-1@[H_3_PW^VI^_12_O_40_] showed high adsorption affinity and capacity towards selective adsorption of heavy metal ions (highly selective for Pb^2+^ and Cd^2+^, but no adsorption of Hg^2+^) from contaminated water.^208^ Studies on HKUST-1@[H_3_PW^VI^_12_O_40_] adsorption ability to remove U(vi) from wastewater showed that it could selectively adsorb U(vi) from low concentration uranium solutions in the presence of other metal ions.^209^ The adsorption capacity of HKUST-1@[H_3_PW^VI^_12_O_40_] was strongly pH dependent and did not significantly decrease after three adsorption–desorption cycles. The presence of phosphate groups in the adsorbent structure has a great affinity for radioactive U(vi) ions in an aqueous solution.^210,211^ In this regard, a ship-type nano-cage POM {[C_5_NH_5_]_9_[H_31_Mo^VI^V^V^_12_O_24_Co^II^_12_(PO_4_)_23_(H_2_O)_4_]}^2−^ (Co-POM) with 23 {PO_4_} groups was designed and synthesized. The high adsorption capacity of this POM-based inorganic framework for U(vi) ions in aqueous solution was mainly ascribed to coordination interaction between U(vi) and O in the phosphate groups on Co-POM which was proved by FT-IR and XPS analyses.^212^ Composites of POMs (H_3_PW^VI^_12_O_40_) with graphene oxide also exhibited a significant potential for uranyl uptake from wastewater.^113^

**Fig. 8:**
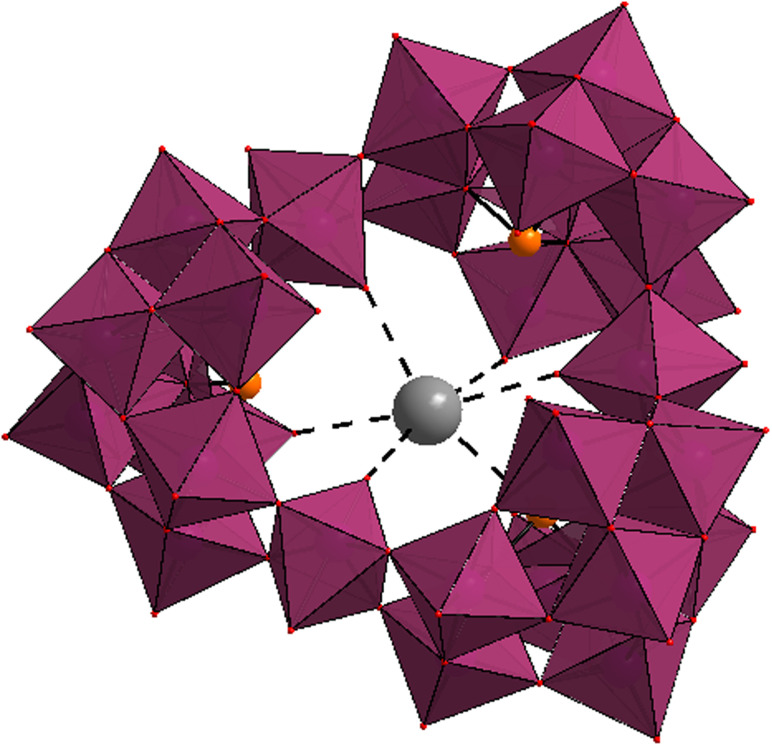
Structure of [M⊂{(β-As^III^W_8_O_30_)(WO(H_2_O))}_3_]^14−^ with the central guest being either K^+^ or Rb^+^. Color code: WO_6_ (violet octahedra), As (orange), K/Rb (grey).^114^

The cation exchange studies by POMs have been widely used to separate radioactive metal ions from radioactive wastes.^109^ Kortz's group worked on a cyclic K^+^-templated POM, [K⊂{(β-As^III^W_8_O_30_)(W^VI^O(H_2_O))}_3_]^14−^, which showed high selectivity to Rb^+^, due to the relatively large size of the central cavity for K^+^ (Fig. 8).^114^ Uchida's group combined the Keggin cluster [SiMo^VI^_12_O_40_]^4−^ anions with a cationic oxo-centered trinuclear complex, to produce ionic crystals with isolated pores, (etpyH)_2_[Cr_3_O(OOCH)_6_(etpy)_3_]_2_[SiMo^VI^_12_O_40_]·3H_2_O (etpy = 4-ethylpyridine), which selectively adsorbed Cs^+^ among alkali and alkaline earth metals *via* reduction of the Keggin [SiMo^VI^_12_O_40_] with ascorbic acid.^213^ The previously reported ionic crystal, (mepyH)[Cr_3_O(OOCH)_6_(mepy)_3_]_2_[PMo^VI^_12_O_40_]·5H_2_O (mepy = 4-methylpyridine, mepyH^+^ = 4-methylpyridinium ion), with 1D open channels, was able to incorporate Na^+^ as well as Cs^+^ by the reduction-induced cation exchange processes.^115^ The authors concluded that the high selectivity towards Cs^+^ is due to the existence of closed pores rather than open channels. Despite the high selectivity towards Cs^+^ however, several disadvantages such as the requirement of heating (343 K) and slow adsorption kinetics (12 h to reach equilibrium) limited the widespread application of (mepyH)[Cr_3_O(OOCH)_6_(mepy)_3_]_2_[PMo^VI^_12_O_40_]·5H_2_O. Later, this group overcame disadvantages by utilizing the large-molecular size and easily reducible Wells–Dawson-type of POMs [P_2_Mo^VI^_18_O_62_]^6−^ (M = Mo, W).^214^ In comparison with the Keggin-type POM, the larger molecular size and higher reduction potential of Dawson-type POM increased the pore volume and facilitated the reduction-induced Cs^+^ exchange. As expected, the capacity and rate of Cs^+^ uptake increased significantly (with only 1 h to reach equilibrium at room temperature), demonstrating the potential application of these adsorbents for radioactive Cs^+^ (Cs-137) removal.^214^

### 2.3 Removal of organic pollutants

#### 2.3.1 Removal of organic dyes

As shown in Table S2 (SI), the decontamination mechanisms, in the case of organic pollutants, are similar to previously discussed methods for inorganic ones. Adsorption of dye molecules, especially cationic ones, by POMs is strongly governed by solution pH. The selective adsorption of methylene blue (MB) in the presence of methyl orange (MO) over [P_2_W^VI^_18_]/MOF-5 catalyst is spontaneous and endothermic. In addition, the pH value of dye solution should also be carefully controlled to obtain maximum adsorption capacity, because the surface charge of the adsorbent is strongly affected by the pH (pH_PZC_; PZC = point-of-zero charge).^215^ Furthermore, olation and oxolation processes are responsible for the high negative charge on the POM surface at lower pH values.^216^

In a generally accepted approach, photooxidation of dye molecules occurs through generation of free OH˙. The proposed mechanism is based on the photoexcitation of Cs_4_SiW^VI^_12_O_40_ POM and a subsequent hydrogen abstraction reaction which results in the homolytic bond cleavage of H_2_O. The photocatalytic activity of POMs, such as [SiW^VI^_12_O_40_]^4−^, can be enhanced in the presence of semiconductors. In fact, in such heterojunction structures with suitable energy band alignment, photogenerated carriers could be separated more efficiently.^217^ Dye sensitization is another mechanism that may contribute to dye degradation in photocatalytic reactions. Due to the visible-light absorption abilities of the sensitizers, dye-sensitized POM photocatalysts can be excited upon visible-light irradiations. In these cases, the oxidation of dye proceeds through electron transfer between the excited dye (*e.g.*, thionine, phthalocyanine) and LUMO of Keggin ([PW^VI^_12_O_40_]^3−^)^218,219^ or Wells–Dawson-type ([α-P_2_W^VI^_18_O_62_]^6−^ (P_2_W_18_) and [α-P_2_W^VI^_17_O_61_]^10−^)^218^ type POMs.^218,219^

As an interesting example of membrane filtration technology, Yao *et al.*^179^ incorporated surfactant-encapsulated POM microparticles into a PVDF matrix as a microfiltration membrane for the adsorptive removal of the anionic dye reactive black 5 (RB5). The authors prepared spherical microparticles through an ion exchange reaction between a cationic surfactant (DODA·Br) and [PV^V^_2_Mo^VI^_10_O_40_]^5−^. This architecture enhanced the flow rate of the system and dye removal efficiency reached up to 97.5% within 120 min.^179^ A similar concept has been applied in the case of surface-active ionic-liquid-encapsulated POMs.^220^ Ion exchange reaction has also been used to replace small anions in the structure of layered double hydroxides (LDHs) with large polyanions. By this method the surface area of the resulting composite can be enhanced, since the interlayer distances of LDH increase. These composites have been used for the removal of cationic dyes from water; however, the instability of LDH in acidic media may limit their application.^221,222^ In 2005 Zhao and co-workers suggested that an active peroxo species is responsible in the photo-Fenton oxidation of Rhodamine B (RhB) under visible light irradiation. The authors proposed that the active species is formed upon the interaction of reduced POM with H_2_O_2_.^223^ Similarly, in Fenton systems the active species is formed by the coordination of iron to [PW^VI^_12_O_40_]^3−^ POM.^224^ In Fenton-like systems the iron species is replaced with different POMs, like mentioned Keggin^224^ or [HPW^V^_4_W^VI^_8_O_40_]^6−^ POMs.^225^ The radical-based pathways, however, can enhance apparent degradation rate if not properly identified or controlled.

**Fig. 9:**
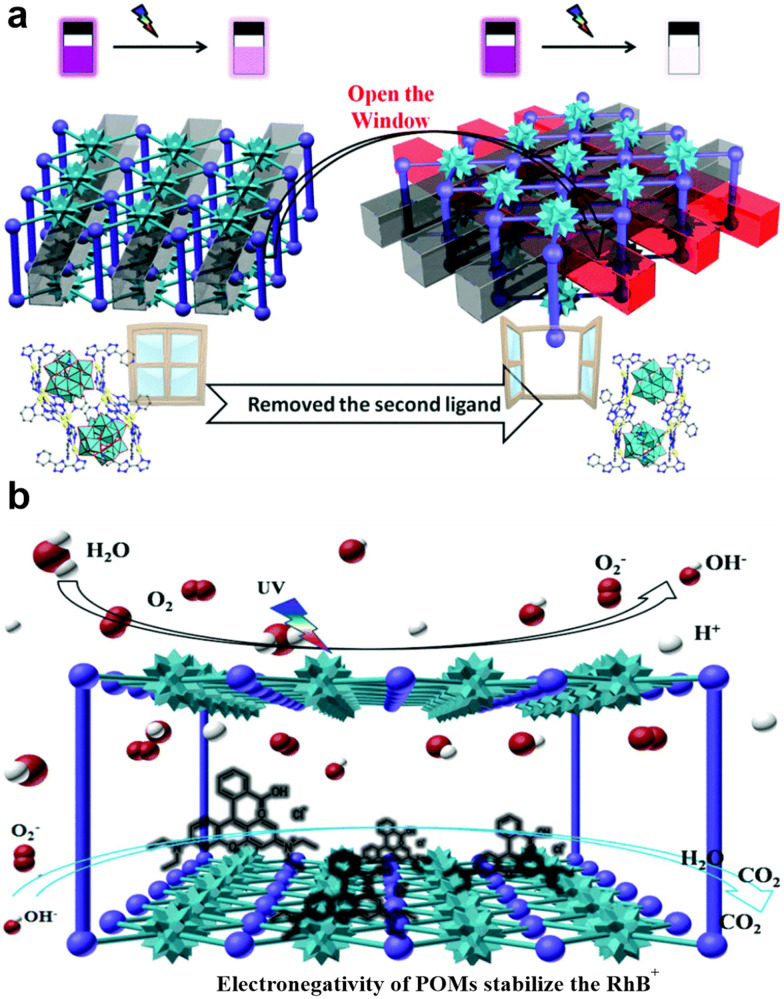
a) Representation of the [Ag_4_(H_2_pyttz-I)(H_2_pyttz-II)(Hpyttz-II)][HSiW_12_O_40_] and [Ag_4_(H_2_pyttz-II)(Hpyttz-II)_2_][H_2_SiW_12_O_40_] compounds ((H_2_pyttz-I = 3-(pyrid-2-yl)-5-(1*H*-1,2,4-triazol-3-yl)-1,2,4-triazolyl) and H_2_pyttz-II = 3-(pyrid-4-yl)-5-(1*H*-1,2,4-triazol-3-yl)-1,2,4-triazolyl) with similar underlying frameworks and different tunnels. b) Representation of the photocatalytic mechanisms for POMCPs. Reproduced from ref. 227 with permission from The Royal Society of Chemistry, copyright 2015.

Among different transition metals (Co, Ni, Cu), Co-substituted Wells–Dawson anions [α_2_P_2_W^VI^_17_CoO_61_]^8−^ exhibited higher catalytic performance.^226^ Li's group prepared two POMCPs, [Ag_4_(H_2_pyttz-I)(H_2_pyttz-II)(Hpyttz-II)][HSiW^VI^_12_O_40_]·4H_2_O (H_2_pyttz-I = 3-(pyrid-2-yl)-5-(1*H*-1,2,4-triazol-3-yl)-1,2,4-triazolyl) and [Ag_4_(H_2_pyttz-II)(Hpyttz-II)_2_][H_2_SiW^VI^_12_O_40_]·3H_2_O (H_2_pyttz-II = 3-(pyrid-4-yl)-5-(1*H*-1,2,4-triazol-3-yl)-1,2,4-triazolyl) with similar structure and different tunnels (Fig. 9a). The photocatalytic degradation of methylene blue (MB) demonstrated that the structure of the hybrids influences the photocatalytic properties. The larger cavities in the compound and [Ag_4_(H_2_pyttz-II)(Hpyttz-II)_2_][H_2_SiW^VI^_12_O_40_]·3H_2_O increase the contact area between catalysts and crude materials and promote more active sites to participate in the reactions process. Thus, the photocatalytic properties of [Ag_4_(H_2_pyttz-II)(Hpyttz-II)_2_][H_2_SiW^VI^_12_O_40_]·3H_2_O were improved. The proposed mechanism for enhanced photocatalytic activity in these hybrids is shown in Fig. 9b. This mechanism includes LMCT from the HOMO to the LUMO, which was facilitated by Ag–O bridging units. In addition to this, Ag-pyttz acted as photosensitizers and promoted the transition of electrons onto [SiMo^VI^_12_O_40_]^4−^ POMs. Therefore, the [SiMo^VI^_12_O_40_]^4−^ POMs had a higher charge density and exhibited a considerable impact on the photocatalytic degradation of RhB.^227^

#### 2.3.2 Removal of aromatic hydrocarbons

The oxidative potential of POMs has been broadly used in AOPs for phenol oxidation.^228^ For example, [PW_11_O_39_Fe^III^(H_2_O)]^4−^ can degrade chlorophenol (CP) compounds only if H_2_O_2_ is added to the solution. No photocatalytic activity was observed in aerated aqueous solution. In addition, the reaction rate was influenced by the initial concentration of the catalyst or H_2_O_2_ and the number of chlorines in the aromatic ring of CP.^228^ Iron-containing POMs have also been used to construct heterojunction photocatalysts by grafting Fe-POM nanoclusters onto oxygen-deficient TiO_2_. The synergistic effect between photocatalysis and Fenton-like reactions resulted in efficient degradation of sulfosalicylic acid (SSA).^229^ Deposition of Au NPs on the surface of POM/TiO_2_ is another strategy to improve light absorption and activity of the catalyst. A 4.6-fold increase was observed in photocatalytic degradation of nitrobenzene (NBZ).^230^ Zhang *et al.* prepared a ferrocene-containing silicotungstate catalyst *via* a co-precipitation method for the photocatalytic oxidation of 4-chlorophenol (4-CP). It was suggested that the synergism between ferrocene and silicotungstate leads to the charge-transition from ferrocene to the POM unit, which ultimately contributes to the oxidation of the organic pollutant through a Fenton-like mechanism.^231^ In another study, [Cs_3_PMo^VI^_12_O_40_] was used as a modifier of the semiconductor Bi_2_O_3_. The experimental results indicated that the [Cs_3_PMo^VI^_12_O_40_] generated on the surface of the semiconductor creates a P–N heterojunction photocatalyst with visible-light activity in the degradation of phenol. The best photocatalytic performance was observed when 2.5% (mol) of [Cs_3_PMo^VI^_12_O_40_] was added to the semiconductor. Also, trapping experiments showed that the major active species involved in the degradation process are superoxide and hydroxyl radicals.^232^ Heterogenization of POMs with graphene aerogels (GA) has also shown promise in the adsorptive removal of a series of organic compounds from water.^233^ A more comprehensive analysis of the studies from the past 5 years is provided in Table S2 in SI.

### 2.4 Summary of water treatment technologies by polyoxometalates

Although the literature review shows promising evidence on how POM-based materials have attracted considerable attention for water treatment, like any emerging technology, they also have their own set of challenges and limitations. As tabulated in Table S2, POM-based materials have often been utilized as photocatalysts with high removal efficiencies. A key negative result that is rarely reported, but likely exists, is the structural instability of POM-based photocatalysts under realistic water matrices (containing chloride, carbonate, or natural organic matter). Such components can significantly suppress the photocatalytic activity or even partially decompose the structure, yet these effects are often not disclosed. Acknowledging this limitation is important for assessing the practical applicability of POM materials. For their broad implementation, they must also maintain the cost of processed water as low as possible. In this regard, substantial costs associated with synthesizing POMs and their composites remain as a significant challenge. In terms of the technology itself, other economically beneficial methods such as adsorption and ion exchange should also be considered, as they tend to provide more affordable solutions for water purification.

## 3 Removal of emerging health pollutants

Some of the most prominent classes of emerging health pollutants (EPs) are pharmaceuticals (antibiotics, antifungals, antidepressants, synthetic hormones)^12,13,18,28,234^ plant protection products (pesticides, biocides),^31,235^ and microplastics.^235–237^ Excessive use of antibiotics and cosmetic products, *e.g.*, disinfectants and cleaning products, has led to the development of bacterial resistance through DNA mutations of bacterial cells, which have resulted in the adaptation and resistance of bacteria to these products.^24,25,238^ In addition, bacterial resistance also occurs through the horizontal gene transfer mechanism from resistant bacteria to non-resistant bacteria through transformation, transduction, or conjugation.^25^ Moreover, water bodies containing EPs play an essential role in this horizontal gene transfer mechanism by facilitating the horizontal gene transfer from pathogenic to non-pathogenic microorganisms. In addition to contributing to the development of antibiotic resistance, pollutants such as UV filters from sunscreens have been shown to harm marine life significantly. These compounds accumulate in aquatic environments and negatively affect organisms, including phytoplankton, corals, microalgae, and sea urchins, by disrupting their physiology and ecosystem functions.^24,239^

A study, conducted over two consecutive years (2015 and 2016), on the final effluents from wastewater treatment plants in Europe, revealed high average concentrations of antibiotics in wastewater, especially in countries such as Portugal, Spain, and Ireland. The study identified that the most commonly found antibiotics, ciprofloxacin, azithromycin, and cephalexin, have a potentially significant impact on aquatic systems and the development of antibiotic resistance.^24,240^

**Fig. 10:**
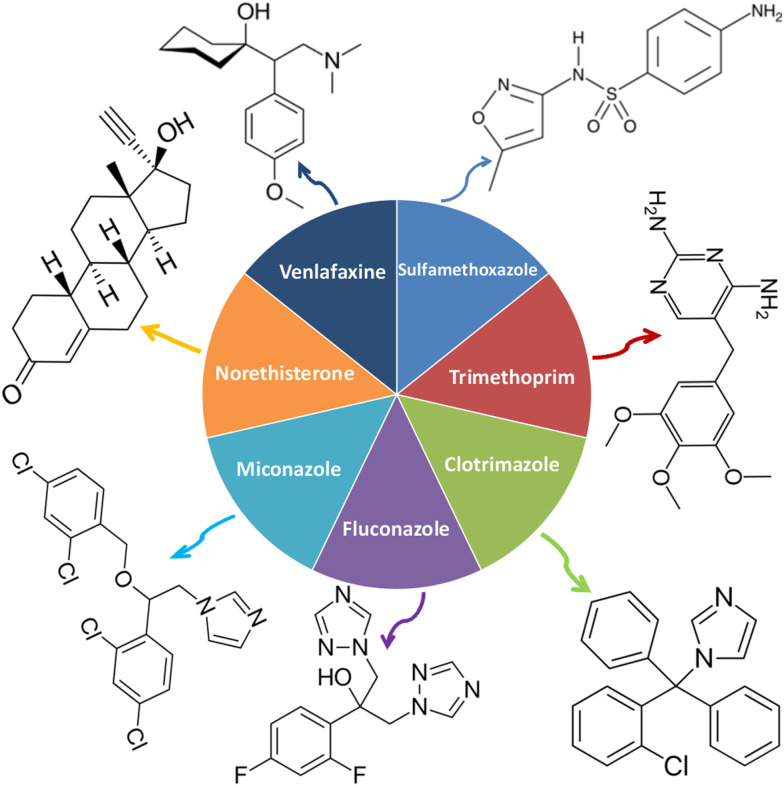
Emergent pharmaceuticals pollutants included in the updated 4th water watch list under the European Water Framework Directive: the antibacterial sulfamethaxazole and trimethoprim; the anti-fungus clotrimazole, fluconazole and miconazole; the antidepressant venlafaxine and the synthetic hormone norethisterone.^17^

Ciprofloxacin, a fluoroquinolone antibiotic, and erythromycin have also been detected in effluents and surface waters in other studies,^24^ and are included, along with the macrolides azithromycin and clarithromycin, as well as the penicillin-type antibiotic amoxicillin, in the surface water Watch List under the European Water Framework Directive.^17,240,241^ More recently, this report has been updated to include other pharmaceutical products such as the antibacterials sulfamethoxazole and trimethoprim, the antifungal clotrimazole, fluconazole, and miconazole, the antidepressant venlafaxine, and the synthetic hormone norethisterone (Fig. 10).^240,241^ In addition to the aforementioned pharmaceuticals, such as proton pump inhibitors (PPIs), lansoprazole and omeprazole,^242,243^ have been proposed as potential Watch List candidates due to their recently discovered possible mutagenic and toxic effects on aquatic organisms.^17,25,240^

Herein, we focus on the POMs' ability to degrade priority pharmaceuticals, mainly antibiotics, pesticides, microplastics, and dyes, to identify POMs with higher removal efficiency and kinetics, thus facilitating the development of more environmentally friendly POM materials.^244,245^

### 3.1 Removal of pharmaceutical pollutants

Every day, humans release pharmaceutical products into the environment in different forms and under different circumstances. This behavior of humanity has a major impact on health and economy and has a profound effect on our lives. It is therefore of great importance to conduct environmental protection in an effective and inexpensive manner to combat emerging health pollutants. Some of the most prominent classes of emerging pharmaceutical pollutants are the antimicrobial pharmaceuticals (antibiotics, antifungals) and other pharmaceuticals (antidepressants, synthetic hormones). It has been described that contamination of the environment with these pharmaceutical products can lead to bacterial resistance, which is an emerging and growing phenomenon worldwide in the 21st century.^10,20,22,24,246^ Nonconventional low-cost adsorbents for pharmaceutical removal from wastewater, pollutant removal mechanisms, and detection using nanodevices and polymer-based adsorbents, as well as using fungal treatments, were recently summarized.^12,13,18^ POMs have also been used for the detection of several pharmaceuticals, such as drugs of abuse^247^ and triclosan (TCS),^248^ as well for the selective extraction of antidepressants in undiluted urine.^249^ TCS, a diphenyl ether with antibacterial properties, is used as a disinfectant in antiseptic creams, toothpaste, hand soaps, deodorants, and even in plastics.^21,22^ In Europe, TCS is one of the most frequently detected contaminants in wastewater. However, studies from the United States have reported that its concentration in wastewater can be up to five times higher.^22^ TCS has already been detected in surface waters in several regions of the world, including in fish tissues. In fact, the methylated form of TCS (M-TCS) is bioaccumulative in tissues, due to its lipophilic properties and stability. Moreover, it has been described that contamination of the environment with TCS can lead to bacterial resistance to four antibiotics: chloramphenicol, tetracycline, ciprofloxacin, and colistin. This resistance poses potential risks to human health as well as aquaculture.^21,22^

**Fig. 11:**
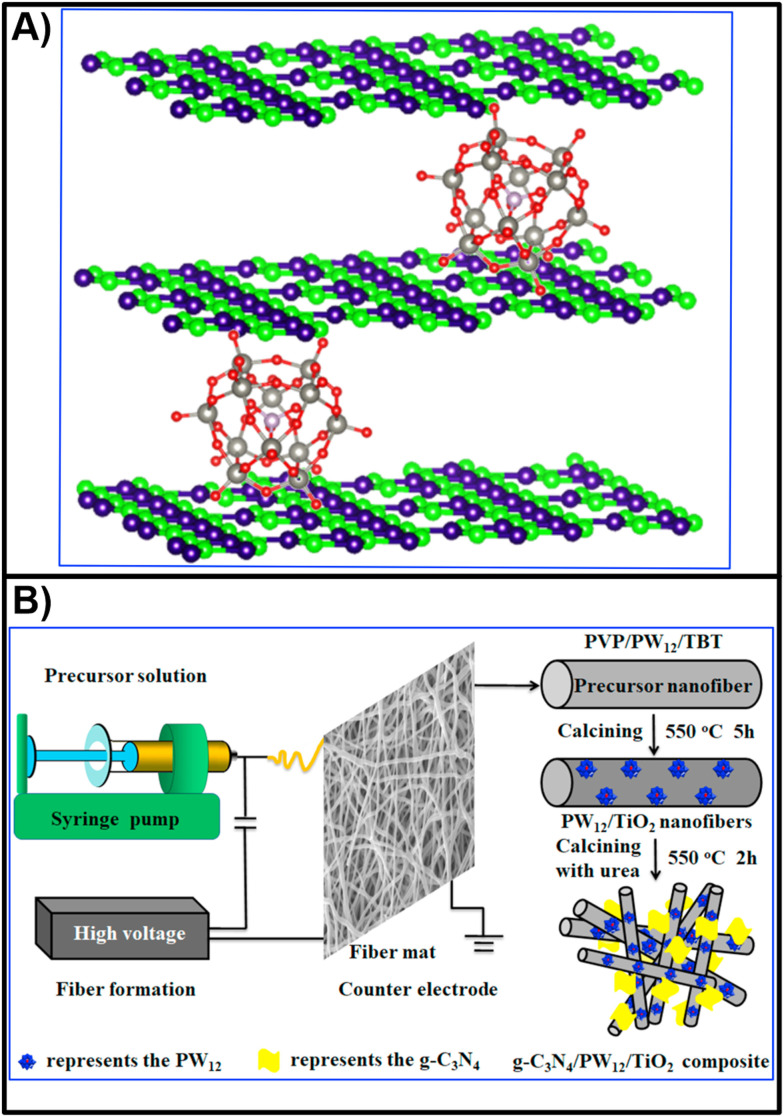
A) Nitrogen-deficient g-C_3_N_*x*_/POMs porous nanosheets (where *x* denotes N-deficiency) with P–N heterojunctions capable of photocatalytic degradation of drugs; recreated from ref. 250 with permission from The Royal Society of Chemistry. B) Fabrication of g-C_3_N_4_/PW_12_/TiO_2_ composite with enhanced photocatalytic performance under visible light; reproduced from ref. 251 with permission form Elsevier, copyright 2021.

Of the seventeen pharmaceutical pollutants mentioned above, only one study has referred to the removal of ciprofloxacin by POMs. He *et al.* immobilized three Keggin-type POMs [H_3_PMo^VI^_12_O_40_]·*n*H_2_O, [H_3_PW^VI^_12_O_40_]·*n*H_2_O, and [H_3_PW^VI^_12_O_40_]·*n*H_2_O onto nitrogen-deficient carbon nitride nanosheets (g-C_3_N_4_) and successfully utilized all three POM-based composites (Fig. 11A) for the removal of ciprofloxacin within only five minutes under visible light irradiation with 93.1%, 97.4% and 95.6% efficiency, respectively.^250^ This type of POM-based hybrid material was further explored on g-C_3_N_4_/PW_12_/TiO_2_ composites (PW_12_ = [H_3_PW^VI^_12_O_40_]) (Fig. 11A and B),^250,251^ which showed remarkable and stable photocatalytic performance under visible light irradiation, not only for the removal of TC but also for bisphenol A and Cr(vi).^251^ Their removal properties and stability without any observed structural changes in the photocatalyst were attributed to the enhanced adsorption under visible light irradiation, a high specific surface area, effective separation, and photoinduced charge transfer *via* g-C_3_N_4_ and PW_12_.^251^

**Fig. 12:**
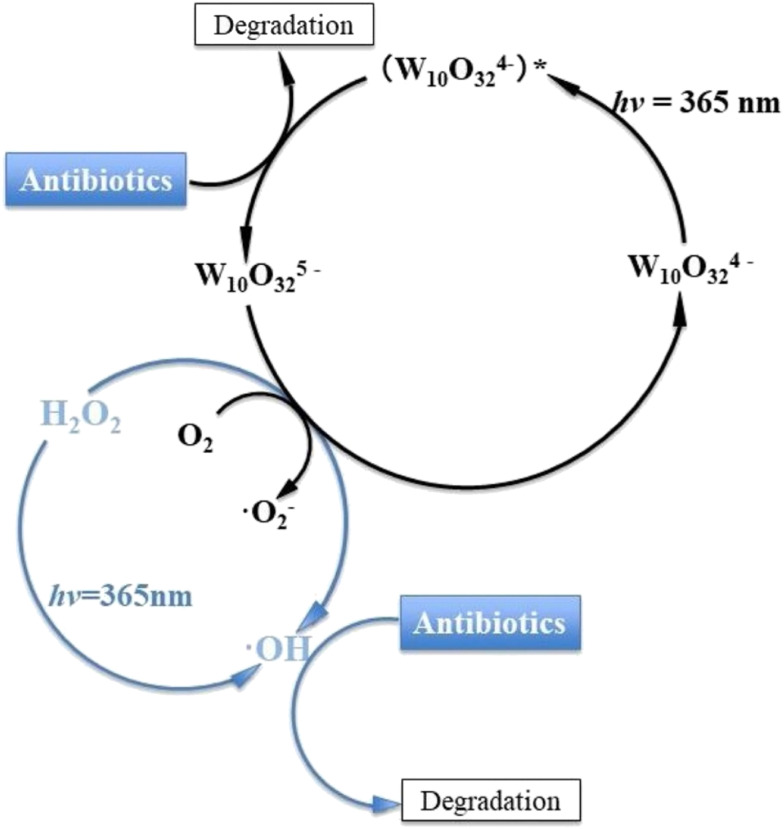
Cycle of photocatalysis and degradation of antibiotics (left) through the isopolyoxometalate decatungstate. Reproduced from ref. 252 with permission from Elsevier, copyright 2002.

**Fig. 13:**
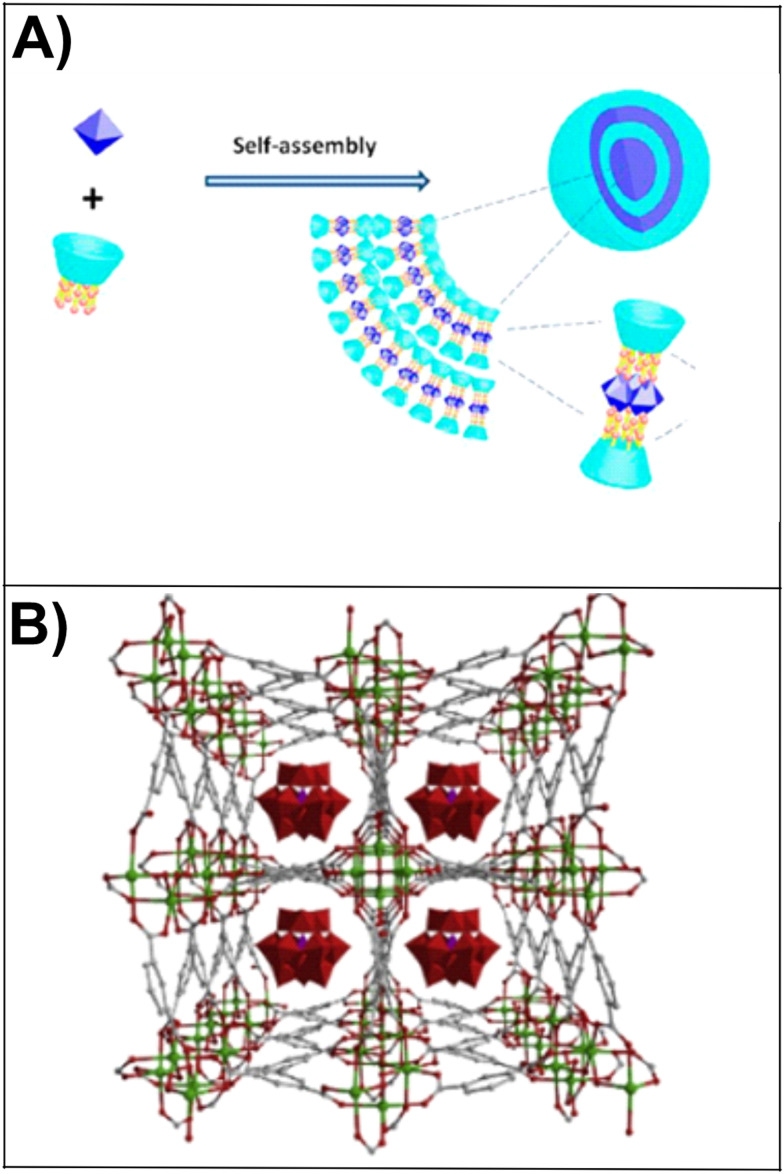
A) Multivalent supramolecular self-assembly between β-cyclodextrin derivatives and polyoxometalate for photodegradation of dyes and antibiotics; reproduced from ref. 256 with permission from The American Chemical Society, copyright 2019. B) Encapsulate polyoxometalate into metal–organic frameworks as efficient and recyclable photocatalyst for drugs degradation; reproduced from ref. 257 with permission from Elsevier, copyright 2019.

Moreover, Cheng *et al.*^252^ have utilized the isopolyoxotungstate, decatungstate [W^VI^_10_O_32_]^4−^ (Fig. 2D) as a photocatalyst for the oxidation of sulfasalazine (SZZ),^253^ an antibiotic commonly found in wastewater, and its human metabolite sulfapyridine (SPD). After 120 min in the presence of H_2_O_2_ and under UV irradiation, the metabolite SPD was more efficiently removed (75%) by decatungstate than was the SZZ antibiotic (25%). The proposed photocatalytic mechanism (Fig. 12), which involves the generation and utilization of hydroxyl radicals (˙OH) in the photocatalytic degradation of sulfasalazine,^252^ has attracted increasing attention over the past decades. This mechanism has been extensively studied in the ongoing research and development of novel pollution removal technologies.^254,255^ Therefore, a similar strategy has been employed for the photodegradation of antibiotics such as nitrofurazone, tetracyclines and berberine under UV or visible light irradiation. This process utilizes H_2_O_2_ and the photoactive POM-based composite [H_3_PW^VI^_12_O_40_]@β-EDA-CD, as shown in Fig. 13A.^256^

Li *et al.* prepared a POM-based photocatalyst, PW_12_@MFM-300(In) (Fig. 13B), by using an environmentally friendly solvent-free method for the encapsulation of the POM [H_3_PW^VI^_12_O_40_] into the metal–organic framework MFM-300(In). The PW_12_@MFM-300(In) composite displayed its activity for room temperature visible-light-driven catalytic degradation of the pharmaceutically active compound SMT with a 98% removal efficiency within 2 h.^257^

### 3.2 Removal of pesticides, microbes and microplastic

POM-based catalysts have been used for decades in pesticide degradation. The decatungstate [W^VI^_10_O_32_]^4−^, mentioned in the context of the removal of pharmaceutical pollutants (section 3.1), also showed photocatalytic activity in the degradation of two common pesticides, 2-(1-naphthyl)acetamide (NAD) and 2-mercaptobenzothiazole (MBT). In the study of da Silva *et al.*, it was shown that [W^VI^_10_O_32_]^4−^ could promote UV-light-driven degradation of NAD with an efficiency of 89% within 8 h.^258^ Additionally, Allaoui *et al.* described the photodegradation of the pesticide MBT using Na_4_W^VI^_10_O_32_ as a catalyst with an efficiency of 90% within 8 h.^259^ It has been proposed that the photodegradation of MBT occurs *via* e^−^ transfer and H-atom abstraction processes with W^VI^_10_O_32_^4−^* excited species. The main products of such photodegradation when using decatungstate as a catalyst are monohydroxylated products, sulfoxide derivatives, and dimers of MBT. The whole process was shown to be O_2_ dependent because photodegradation was restricted by W^VI^_10_O_32_^5−^ reoxidation.^259^ The Keggin-type POM [PW^VI^_12_O_40_]^3−^ showed activity for the complete photocatalytic degradation of the pesticide lindane to CO_2_, H_2_O, and Cl^−^ in an aqueous solution.^260^ Photocatalysis of lindane by [PW^VI^_12_O_40_]^3−^ follows the same principle as that of TiO_2_ catalysis, *i.e.* processes involving both oxidation and reduction pathways such as chlorination, dechlorination, hydroxylation, hydrogenation, dehydrogenation, which lead to the C–C bond cleavage and complete mineralization to the final products.^260^ Recently, a POM-IL^261^ has also been used for the extraction of triazole pesticides (*e.g.*, penconazole, hexaconazole, diniconazole, tebuconazole, triticonazole, and difenconazole) from aqueous samples.^262^ In that article, the prepared POM-IL nanomaterial ([3-(1-methylimidazolium-3-yl)propane-1-sulfonate]_3_PW^VI^_12_O_40_) was utilized as a coating for a new solid-phase microextraction (SPME) device that was then successfully applied for the extraction of the six triazole pesticides from real aqueous samples. The longevity experiments (at least 50 extractions) of POM-IL coated SPME devices compared with commercially available PDMS-coated SPME devices (PDMS = polydimethylsiloxane) showed that the newly prepared device offers higher extraction efficiency and better longevity.^262^ Moreover, the type of POM-IL material (Fig. 6), already described in section 2.2, was shown to efficiently remove previously mentioned inorganic and organic contaminants from wastewater, as well as various microbial pollutants, *E. coli* and *B. subtilis*.^63^ Recent developments in these organic/inorganic hybrid materials, POM-based ionic liquid crystals and POM-ILs, and their applications, mainly in pollutants degradation, including microplastics, have been reported.^263^

**Fig. 14:**
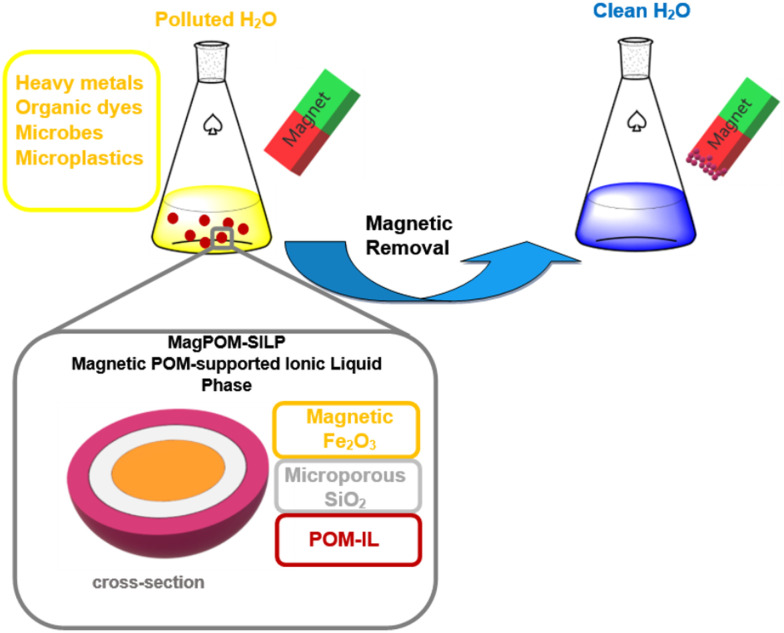
Magnetic polyoxometalate-supported ionic liquid (magPOM-SILPs) for heavy metals, organic dyes, microbes and microplastics water removal.^263^

Microplastics (MPs) are among the newly emergent health pollutants of worldwide concern, and their impact on human health and the environment is not yet completely understood.^264^ The first reported example of magnetic polyoxometalate-based ionic liquid phases (magPOM-SILPs) for the removal of MPs was designed by anchoring a POM-IL composite (POM = [α-SiW^VI^_11_O_39_]^8−^ (Fig. 2F); IL = (*n*-C_7_H_15_)_4_N^+^) to an Si-enclosed Fe_2_O_3_ supermagnetic core, Fe_2_O_3_@SiO_2_ (Fig. 14). The magPOM-SILPs composite showed remarkable effectiveness (90%) for removing microplastic by binding MPs particles *via* the formation of hydrophobic interactions with the MPs surface and then removing MPs pollutants from water samples by magnetic recovery (Fig. 14).^263^

Cobalt-based POMs, Na_10_[Co_4_(H_2_O)_2_(V^V^W^VI^_9_O_34_)_2_]·34H_2_O were also examined for dye degradation. MB and RhB dyes were chosen as the subject dyes for the degradation test because of their carcinogenic properties and wide use in the textile industry. A 10 mg L^−1^ dosage of this POM removed 87.8% of MB in 30 min. The time required for the complete decomposition of RhB was almost twice as long as that of MB. In this study, in addition to the excellent dye catalytic activity, these CoV-POMs also showed anticancer activities.^265^ However, POMs anticancer, antibacterial studies, and other biomedical studies are described elsewhere.^85,266–268^ Another recent study, described the synthesis of two Keggin-type polyoxometalates ammonium phosphomolybdate (NH_4_)_3_PMo^VI^_12_O_40_ (PMo) and ammonium phosphotungstate (NH_4_)_3_PW^VI^_12_O_40_ (PW) that were used as adsorbents for the removal of various antibiotics and heavy metals from water systems. The adsorption efficiency of PMo for dyes and heavy metals was higher than that of PW for various antibiotics such as tetracycline. It was suggested that the more negative surface charges induced by Mo atoms with more electronegativity and higher specific surface area contributed to the superior adsorption efficiency of PMo for antibiotics and heavy metals.^269^

Table 1 summarizes the recent examples of POMs applications in removal of EPs covered in section 3.

**Table 1:** Examples of recent polyoxometalates studies in pollutants degradation: antibiotics (A), dyes (D), plastics (P), industrial chemicals (IC) and pesticides (Pest)

Formula	POM archetype	Pollutant	Conditions	Efficiency	Number of cycles	Ref.
Na_4_W^VI^_10_O_32_	Decatungstate	Sulfasalazine	*c*(catalyst) = 40 μM; under UV irradiation	25% removal within 120 min	1	252
		(A) sulfapyridine		75% removal within 120 min		
g-C_3_N_4_-POMs	Keggin	(A) ciprofloxacin	*m*(catalyst) = 0.01–0.1 g; under visible light	93% removal within 5 min	1	250
POMs: [PMo^VI^_12_O_40_]^3−^, [PW^VI^_12_O_40_]^3−^, [SiW^VI^_12_O_40_]^4−^						
g-C_3_N_4_/H_3_PW^VI^_12_O_40_/TiO_2_	Keggin	(A) tetracycline	*m*(catalyst) = 20 mg	>70% removal within 50 min (*k* = 0.03443 min^−1^)	1	251
		(P) bisphenol A	*m*(catalyst) = 20 mg	>38% removal within 3 hours (*k* = 0.00712 min^−1^)	1	
		(IC) Cr(vi)	*m*(catalyst) = 20 mg	>65% removal within 60 min (*k* = 0.025 min^−1^)	1	
POM-IL, [3-(1-methylimidazolium-3-yl) propane-1-sulfonate]_3_PW^VI^_12_O_40_	Keggin	(Pest) diniconazole	nsp	nsp	1	262
		(Pest) hexaconazole	nsp	nsp	1	
		(Pest) tebuconazole	nsp	nsp	1	
		(Pest) penconazole	nsp	nsp	1	
		(Pest) diniconazole	nsp	nsp	1	
		(Pest) triticonazole	nsp	nsp	1	
Biochar-doped g-C_3_N_4_-Co_2_PMo_11_VO_40_	Keggin	(A) sulfamethoxazole	*m*(catalyst) = 0.2 g L^−1^; under visible light	98.5% within 20 min (*k* = 0.215 min^−1^)	1	273
Ag-L-SiW_12_@BiVO_4_ (L = thiacalix[4]arene)	Keggin	(A) ciprofloxacin	pH = 4; *v*(catalyst) = 30 μL; under simulated solar light	95% within 240 min (*k* = 0.0118 min^−1^)	1	274
H_3_PW_12_O_40_–Fe_3_O_4_-biocar	Keggin	(A) metronidazole	pH = 1; *c*(catalyst) = 0.6 g L^−1^	>94% removal within 60 min	1	275
α-K_8_SiW_11_O_39_-MIL-101(Cr)-CoFe_2_O_4_	Lacunary Keggin	(D) methylene blue	*m*(catalyst) = 30 mg	Methylene blue = 100% within 25 min	1	276
		(D) rhodamine B		Rhodamine B = 84% within 50 min		
		(D) methyl orange		Methyl orange = 37% within 20 min		
		(A) ciprofloxacin		Ciprofloxacin = 100% within 15 min		
EDA-CD-[H_3_PW^VI^_12_O_40_], (EDA-CD = per-6-deoxy-6-ethylenediamine-β-cyclodextrine)	Keggin	(A) nitrofurazone	*c*(catalyst) = 0.055 mM; under UV irradiation or sunlight; H_2_O_2_	*k* = 0.163 min^−1^	1	256
		(A) tetracyclines	With H_2_O_2_	*k* = 0.152 min^−1^	1	
		(A) berberine	With H_2_O_2_	*k* = 0.115 min^−1^	1	
		(D) rhodamine B	With H_2_O_2_	*k* = 0.868 min^−1^	1	
		(D) xylenol Orange	With H_2_O_2_	*k* = 0.214 min^−1^	1	
		(D) methyl Orange	With H_2_O_2_	*k* = 0.164 min^−1^	1	
		(D) methylene blue	With H_2_O_2_	*k* = 0.119 min^−1^	1	
		(D) crystal violet	With H_2_O_2_	*k* = 0.084 min^−1^	1	
[H_3_PW^VI^_12_O_40_],@MFM-300(In)	Keggin	(A) sulfamethazine (SMT)	nsp	98% removal within 60 min	1	257
MFM-300(In) = indium-based metal–organic framework						
LnTiO_2_/P_2_W^VI^_18_Sn_3_	Keggin	(D) methyl orange	nsp	100% removal within 5 min	1	270
Na_4_W^VI^_10_O_32_	Decatungstate	(Pest) 2-(1-naphthyl)acetamide (NAD)	*c*(catalyst) = 300 μM	89% removal within 8 hours (*k* = 0.032 min^−1^)	1	258
K_2_[V^V^_10_O_16_(OH)_6_(CH_3_CH_2_CO_2_)_6_]	Decavanadate	(D) methylene blue	*m*(catalyst) = 5 mg	93% removal within 45 min	1	271
[Cu(OH_2_)_3_(2-amp)]_2_(trisH)_2_[V^V^_10_O_28_]	Decavanadate	(D) methylene blue	*m*(catalyst) = 2–10 mg; with H_2_O_2_	93% removal within 2 min	1	272
2-amp = 2-aminopyridine						
Tris = tris(hydroxymethyl)aminomethane						
Na_10_[Co_4_(H_2_O)_2_(V^V^W^VI^_9_O_34_)_2_]·34H_2_O	Keggin	(D) methylene blue	*c*(catalyst) = 10 mg L^−1^	88% removal within 30 min	1	265
		(D) rhodamine B		88% removal within 60 min		
NH_4_PW^VI^_12_O_40_ (PW)	Keggin	(IC) Ni^2+^	*m*(catalyst) = 30 mg	72% removal within 1 min (PW)	1	269
NH_4_PMo^VI^_12_O_40_ (PMo)				90% removal within 1 min (PMo)		
		(D) tetracycline	*m*(catalyst) = 30 mg	71% removal within 30 min (PW)		
				92% removal within 30 min (PMo)		
α-H_3_PW^VI^_12_O_40_·6H_2_O	Keggin	(D) methylene blue	*m*(catalyst) = 5 mg	>90% removal for all dyes within 30 min	1	277
α-H_3_PMo^VI^_12_O_40_·14H_2_O		(D) rhodamine B				
		(D) crystal violet				
		(D) methyl orange				
		(D) sunset yellow				

### 3.3 Summary of POM-based technologies in removal of emerging health pollutants

Section 3 highlights emerging pollutants in the 21st century environment, such as drugs, pesticides, and microplastics, and emphasizes their dangers and consequences for human health. Several examples illustrate the use of pure POMs, nanoparticles, composites, or MOFs for removing organic and inorganic pollutants. The processes involving POMs in pollutant degradation are also discussed, many of which employ photocatalysis by UV and/or visible irradiation, in addition to adsorption or magnetic removal. In short, the different types of POMs mentioned in this section reveal their essential role in removing emerging pollutants from the environment, proving to be efficient and selective.

## 4 Polyoxometalates in air pollution

Various POMs alone and in combination with other compounds,^112,278^ such as MOFs, CNTs and mesoporous silica supports, have shown promising results in the removal of air pollutants, such as refractory sulfur compounds^279^ from fossil fuels (section 4.1), toxic gases such as hydrogen sulfide^116^ (section 4.2.1), nitrogen oxides and sulfur dioxide^280^ (section 4.2.2) and carcinogenic volatile organic compounds (VOCs; section 4.3) present in indoor and outdoor air.^281,282^

Among POM archetypes, Keggin-type structures dominate air purification applications due to their high catalytic activity, particularly in the oxidative desulfurization of refractory sulfur compounds from fossil fuels under mild conditions^61^ (∼85% of the reported literature; Table S1). Anderson–Evans POMs also contribute effectively to the desulfurization of fossil fuels by showing promising desulfurization performance through alkyl peroxide formation mechanisms with extended catalyst lifetimes.^283,284^ Wells–Dawson-type POMs, especially when doped with lanthanide ions, exhibit enhanced regeneration and stability, making them effective for toxic gas removal (section 4.2; Table 2), such as H_2_S, NO_*x*_, and SO_2_. Their tunable redox states and structural differences tailor their catalytic behavior, with rare-earth-doped Wells–Dawson POMs^116^ showing superior H_2_S oxidation and the photocatalytic activity of Keggin/g-C_3_N_4_ composites enabling efficient VOC removal under visible light.^285,286^ These reported examples of using different POM structures highlight the unique functions and advantages that structural diversity in POM chemistry provides for air pollutant remediation.^116,248,282,283,285^

**Table 2:** List of polyoxometalates and POM-based materials utilized in air purification. All POMs are ordered chronologically from the most recent to the oldest published paper

Formula	POM archetype	Conditions	Efficiency	Number of cycles	Ref.
Removal of H_2_S					
PMo_12_@RH-MCM-14	Keggin (Fig. 2F)	*T* = rt; *t* = 120 min; *m*(catalyst) = 0.3 g; *c*_0_(H_2_S) = 1000 mg m^−3^; flow rate = 100 mL min^−1^ (N_2_/H_2_S gas mixture)	61.3% yield of H_2_S transformation to S	More than 8	327
PMo_12_ = [H_3_PMo^VI^_12_O_40_]					
(Himi)_2_[S^VI^Mo^VI^_12_O_40_]·(imi)_2_·H_2_O	Keggin (Fig. 2F)	*T* = 0–50 °C; pH = 4–9; *c*(POM) = 1 mmol L^−1^; *c*(H_2_S) = 2 g m^−3^; flow rate = 100 mL min^−1^ (N_2_/H_2_S gas mixture)	H_2_S capacity in water: 627 mg g^−1^; after electro treatment up to 2174 mg g^−1^	4 cycles	328
imi = imidazole					
(*n*-Bu_4_N)_3_[VMo^VI^_12_O_40_]/[Bmim]Oac	Keggin (Fig. 2F)	*T* = 150 °C; *c*(POM) = 0.005 mol L^−1^; flow rate = 100 mL min^−1^ (N_2_/H_2_S gas mixture); *t* = 10 h	98.6% within 10 h	At least 4 cycles	329
[Bmim] = 1-butyl-3-methylimidazolium					
(NH_4_)_11_[Ln^III^(PMo^VI^_11_O_39_)_2_]	Lacunary Keggin (Fig. 2G)	*T* = rt; pH = 5; *t* = 360 min; *c*(catalyst) = 0.002 M; *c*_0_(H_2_S) = 2900 mg m^−3^	94.8% within 360 min	At least 4	330
Ln = Sm, Ce, Dy and Gd					
K_17_[Pr^III^(P_2_Mo^VI^_17_O_61_)_2_]	Wells–Dawson (Fig. 2H)	*T* = 25 °C; pH = 6.8; *t* = 400 min; *c*(catalyst) = 0.015 M; *c*_0_(H_2_S) = 2200 mg m^−3^	90% within 400 min	nsp^a^	116
K_17_[Gd^III^(P_2_Mo^VI^_17_O_61_)_2_]					
K_17_[Sm^III^(P_2_Mo^VI^_17_O_61_)_2_]					
K_17_[Eu^III^(P_2_Mo^VI^_17_O_61_)_2_]					
[C_4_mim]_3_[PMo^VI^_12_O_40_]-[C_4_mim]Cl	Keggin (Fig. 2F)	*T* = 80–180 °C; *t* = 60 min; H_2_S flow rate 100 mL min^−1^; *c*(catalyst) = 0.001 M	100% within 60 min	More than 6	299
[C_4_mim] = 1-butyl-3-methylimidazolium					
TM-salts of [H_4_PMo^VI^_11_V^V^O_40_],	Keggin (Fig. 2F)	*T* = 25 °C; *t* = 300 min; H_2_S gas flow = 200 mL min^−1^; *c*_0_(H_2_S) = 1241 mg m^−3^; *c*(catalyst) = 0.01 M, H_2_O_2_ – oxidant	98% within 300 min	nsp^a^	54
(TM = Cu^II^, Fe^III^, Zn^II^, Mn^IV^ and Cr^VI^)					
PyBs-PW, PhPyBs-PW and QBs-PW	Keggin (Fig. 2F)	*T* = 70 °C; *t* = 10 min; *n*(H_2_S)_0_ = 1 mmol, 30% H_2_O_2_ (*n* = 1 mmol); solvent mixture H_2_O/EtOH (v : v = 7 : 3); *m*(catalyst) = 80 mg	98% within 10 min	At least 5	331
PW = H_3_PW^VI^_12_O_40_					
[{(CH_3_)_4_N}_4_Cu^II^PW^VI^_11_O_39_H]	Lacunary Keggin (Fig. 2G)	*T* = rt.; *t* = 20 h; *m*(cat.) = 10 mg; *c*(H_2_S)_0_ = 0.1 M	95.0% within 20 h	At least 2	300
[Na_2_HPMo^VI^_12_O_40_]	Keggin (Fig. 2F)	*T* = 20 °C; *c*(catalyst) = 1.25 × 10^−2^ M; *c*_0_(H_2_S) = 240.72 mg m^−3^; H_2_S gas flow = 0.5 L min^−1^	Sulfur loading capacity of 1.14 mol of H_2_S per mol of POM	nsp^a^	332
[Na_3_PMo^VI^_12_O_40_]	Keggin (Fig. 2F)	*T* = rt; *t* = 47 min; *c*(adsorbent) = 5 × 10^−3^ M; *c*_0_(H_2_S) = 500.863 mg m^−3^; H_2_S gas flow = 3.931 L min^−1^	Up to 99.67% within 35–50 min	nsp^a^	333
([Na_3_PMo^VI^_12_O_40_] : NaVO_3_ : Na_2_CO_3_ : NaCl = 1 : 1 : 0.377 : 5.472)					
PCDES@3C_14_-2Im	Keggin (Fig. 2F)	*T* = 25–200 °C; *c*(adsorbent) = 0.01 mol L^−1^; *t* = 150 min, H_2_O_2_; 12.93 mg H_2_S per g adsorbent	Up to 100% within 150 min	At least 5	334
PCDES = long-chain ionic liquid hybrid POM deep eutectic solvent, POM present as [C_14_mim]_3_PMo^VI^_12_O_40_					
[C_14_mim] = 1-tetradecyl-3-methylimidazolium					
PPILs@IBuPN-9	Keggin (Fig. 2F)	*T* = 100–200 °C; *t* = 2 h; *c*(PPILs@IBuPN-9) = 0.015 mol L^−1^; 21.88 mg H_2_S per g PPILs@IBuPN-9	Up to 100% fpr 120 min	At least 4 cycles	335
PPILs = phosphazene POM ionic liquid, 1-bityl-3-methylimidazolium chloride with phosphazenes and H_3_PMo^VI^_12_O_40_					
PMo_12_@UiO-66@H_2_S-MIP-β-CDs	Keggin (Fig. 2F)	*T* = room temperature; 31.67 mg H_2_S per g; *m*(adsorbent) = 0.3 g, H_2_S 1000 mg m^−3^	Up to 31.67 mg g^−1^ H_2_S within 150 min	5 cycles	336
CD = β-cyclodextrin, MIP = molecular imprinted polymers; UiO-66 = metal–organic framework					
PMo_12_-BmimCl@SiO_2_-0.05%	Keggin (Fig. 2F)	*m*(PMo_12_-BmimCl@SiO_2_–0.05%) = 5 g; H_2_S 1000 mg m^−3^, flow rate = 100 mL min^−1^; *T* = 100–200 °C	97% desulfurization for 480 min	3 cycles	337

BmimCl = 1-butyl-3-methylimidazolium chloride					

Removal of NO_*x*_ and SO_2_					
PW_12_@Bi_2_O_3−*x*_/Bi	Keggin (Fig. 2F)	LED lamp (*λ* > 420 nm); *m*(catalyst) = 0.3 mg; *c*(NO) = 600 ppb (in air mixture), flow rate (NO) = 500 mL min^−1^	83.3% within 30 min (in gas phase)	nsp^a^	338
PW_12_ = H_3_PW^VI^_12_O_40_					
*x* = nsp					
[H_4_GeW^VI^_12_O_40_](HGeW), [H_5_GeW^VI^_11_V^V^O_40_] (HGeWV)	Keggin (Fig. 2F)	*T* = 100–350 °C; rate = 4 °C min^−1^; *t* = 90 min; *c*(NO_*x*_) = 1696 mg m^−3^; *c*(O_2_) = 8 vol%; *c*(H_2_O vapor) = 5 vol%	81.5% NO_*x*_ removal with N_2_ selectivity of 68.3% within 90 min	At least 3	314
[H_5_GeMo^VI^_11_V^V^O_40_] (HGeMoV)					
[H_5_GeW^VI^_9_Mo^VI^_2_V^V^O_40_] (HGeWMoV)					
H_6_P_2_W^VI^_18_O_62_·28H_2_O	Wells–Dawson (Fig. 2H)	*T* = 50–200 °C; *t* = 60 min; *c*_0_(NO_*x*_) = 1696 mg m^−3^; *c*(O_2_) = 8 vol%; *c*(vapor) = 4.5 vol%	Up to 90% of NO_*x*_ adsorption within 60 min	At least 2	339
[Fe^III^(C_4_H_5_NO_4_)]_3_[PW^VI^_12_O_40_]·14H_2_O	Keggin (Fig. 2F)	*T* = 50 °C; *t* = 15 min; *c*(H_2_O_2_) = 4 mol L^−1^; pH = 5.5; *c*_0_(NO) = 603 mg m^−3^	94.6% within 15 min	3	312
(Fe^III^AspPW)					
Ce^IV^O_2_/H_3_PW^VI^_12_O_40_	Keggin (Fig. 2F)	*T* = 160–220 °C; *t* = 30 min; *c*_0_(NO) = 600 mg m^−3^; *c*(NH_3_) = 600 mg m^−3^	90% NO removal within 30 min	nsp^a^	340
H_4_[(Cu_4_Cl)_3_(BTC)_8_]_2_[SiW^VI^_12_O_40_]·(C_4_H_12_N)_6_·3H_2_O (NENU-15)	Keggin (Fig. 2F)	*T* = 20–300 °C; *c*(NO) = 1.74 mmol g^−1^; *m*(cat.) = 0.2 g; gas mixture NO (5%) and He (95%), gas flow rate = 30 mL min^−1^	NO adsorption efficiency of 1.74 mmol g^−1^ of NO at rt, and 64% efficiency at 300 °C	nsp^a^	341
[Fe^III^(C_4_H_5_NO_4_)]_3_[PW^VI^_12_O_40_]·14H_2_O	Keggin (Fig. 2F)	*T* = 65–80 °C; *t* = 15 min; *c*(NO)_inlet_ = 614 mg m^−3^; *c*(SO_2_)_inlet_ = 2094 mg m^−3^; *c*(catalyst) = 0.5 g L^−1^	84.27% (NO) and 100% (SO_2_) within 15 min	3	280
(Fe^III^AspPW)					
HPW^VI^–M/Ce^IV^_*x*_Zr^IV^_4−*x*_O_8_ and HPW^VI^–M/Ti^IV^_*x*_Zr^IV^_1−*x*_O_4_	Keggin (Fig. 2F)	*T* = 170–250 °C; *t* = 31–32 min; *m*(catalyst) = 300 mg, gas mixture: NO = NO_2_ = 500 ppm, O_2_ = 10%, CO_2_ = 5%, H_2_O = 5%	48% NO_*x*_ reduction efficiency and 84% NO_*x*_ storage efficiency within 31–32 min	12	342
(M = Pt^IV^, Pd^II^ or Rh^III^ (1 wt%); Zr^IV^/Ce^IV^ = 0.5; Zr^IV^/Ti^IV^ = 0.5)					
H_3_PW^VI^_12_O_40_·6H_2_O (HPW)	Keggin (Fig. 2F)	*T* = 80–170 °C; *m*(HPW) = 330 mg; gas mixture: NO = NO_2_ = 500 ppm, O_2_ = 10%, CO_2_ = 5%, H_2_O = 5%	NO_*x*_ adsorption amount is equal to 38 mg g^−1^ of HPW	6	343
[(NH_4_)_3_PW^VI^_12_O_40_]	Keggin (Fig. 2F)	*T* = 150 °C; *t* = 60 min; He gas flow = 15 mL min^−1^; *n*(NO_2_) = 17.0 μmol	68% NO_2_ removal within 60 min	3	344
MnCeO_*x*_–SiW, where SiW = H_4_[SiW^VI^_12_O_40_]	Keggin (Fig. 2F)	Gas mixture: 100 ppm chlorobenzene, 500 ppm NO and 500 ppm NH_3_, 11 vol% O_2_; *T* = 120–180 °C; *t* = 30 min; *m*(catalyst) = 200 mg	100% NO and chlorobenzene conversion at 180 °C	nsp^a^	345
10HPW-CS-Ce_0.3_–TiO_2_,	Keggin (Fig. 2F)	Gas mixture: 50 ppm chlorobenzene, 500 ppm NO, 500 ppm NH_3_, 5 vol% O_2_, and N_2_ as balance gas; *m*(catalyst) = 100 mg; *T* = 167–291 °C	100% conversion of NO at 167–288 °C, 90% conversion of chlorobenzene at 291 °C	nsp^a^	346
HPW = H_3_PW^VI^_12_O_40_, CS = chitosan					

Removal of aldehydes					
[SiW^VI^_9_O_37_Ru^III^_3_(H_2_O)_3_Cl_3_]^7−^/CSH	Keggin (Fig. 2F)	*T* = rt; *c*(CH_2_O) = 833 ppm ± 10%; CH_2_O gas flow rate = 0.25 dm^3^ min^−1^; *m*(catalyst) = 110 mg	44% for 1st cycle	5	327
CSH = cellulose propylamine-modified silica					
[*n*-Bu_4_N]_4_H_5_PW^VI^_6_V^V^_6_O_40_·20H_2_O (PW_6_V_6_)	Keggin (Fig. 2F)	*T* = rt; *t* = 144 h; *c*(CH_2_O) = 0.52 mol L^−1^; *P*(air) = 1 atm; *c*(catalyst) = 3.8 mmol L^−1^; solvent–DMA : H_2_O (v/v = 20/1); *v*(solvent) = 2 mL	Up to 42% of CH_2_O conversion within 144 h	At least 3	347
[*n*-Bu_4_N]_6_[PW^VI^_9_V^V^_3_O_40_] (PW_9_V_3_)					
[*n*-Bu_4_N]_5_H_2_PW^VI^_8_V^V^_4_O_40_ (PW_8_V_4_)					
H_5_PMo^VI^_10_V^V^_2_O_40_/APTS/SBA-15	Keggin (Fig. 2F)	*T* = 20 °C; *t* = 24 h; *m*(catalyst) = 0.1 g; *v*(O_2_) = 500 mL; O_2_ – oxidant	Up to 73% acetaldehyde conversion after 24 h	5	348
H_6_PMo^VI^_9_V^V^_3_O_40_/APTS/SBA-15					
H_4_PMo^VI^_11_V^V^O_40_/APTS/SBA-15					
APTS = γ-aminopropyltriethoxysilane					
SBA-15 = aminosilylated silica					
NaH_3_[SiW^VI^_11_Ce^IV^O_39_]	Keggin (Fig. 2F)	*T* = 20–60 °C; *t* = 5 h; *P* = 1 atm; *c*(CH_2_O) = 4 mM; *c*(catalyst) = 5.2 mM; solvent H_2_O	85% CH_2_O conversion within 5 h	30	278
TBA_4_HPW^VI^_11_Co^III^O_39_	Keggin (Fig. 2F)	*T* = 20–40 °C; *t* = 6 h; *P* = 1 atm; *m*(catalyst) = 100 mg; solvents: MeCN or H_2_O	92% conversion of isobutyraldehyde	At least 3	282

a nsp – not specified by authors.

### 4.1 Removal of refractory sulfur compounds from fossil fuels

The governments worldwide have introduced stricter regulations and restrictions on the amount of sulfur in fuels to ultra-low levels (<10 ppm).^53^ Therefore, the main goal of industry and science is to find a way to make the fuel desulfurization method efficient, inexpensive, clean, and safe.^52,53^ Currently, the established industrial standard for fossil fuel desulfurization is hydrodesulfurization (HDS). The HDS method has proven itself to be very effective in removing thiols, inorganic sulfides, and disulfides. However, due to new regulations requiring ultra-low sulfur fuels,^53^ HDS is insufficiently effective for removing the more difficult-to-remove refractory sulfur compounds. Moreover, HDS is a very expensive method and operates under harsh reaction conditions of 300–400 °C and 30–100 bar H_2_ pressure. In contrast, POM-based oxidative desulfurization (ODS) operates under mild conditions (rt −100 °C, atmospheric pressure, H_2_O_2_/O_2_ (Table S1)). POMs provide competitive advantages for the needed ultra-low sulfur fuels (<10 ppm)^53^ through their reversible multi-electron redox capability, oxygen-rich surfaces, and high catalytic stability. This eliminates high-pressure H_2_ handling and reduces energy demands for heating and compression.^52,61^ ODS-based systems achieve 84–98% sulfur conversion from 3.5 wt% to <0.5 wt% with 55.57% energy efficiency, demonstrating superior energy utilization for refractory sulfur compounds like DBTs.^287^ Electrochemical regeneration (H_2_O_2_/O_2_) further enhances POM recyclability (in most reported literature: >95% recovery, and 10+ cycles; Table S1). These data show that the ODS system is more energy cost-efficient for deep desulfurization than HDS.^287,288^

**Fig. 15:**
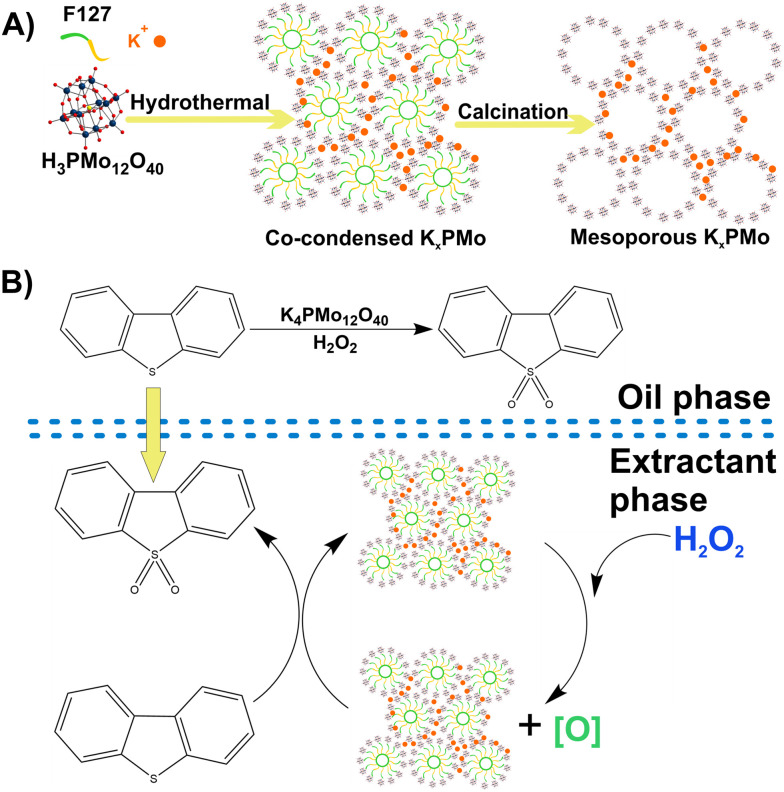
A) Illustration of one-pot hydrothermal synthesis of mesoporous K_*x*_PMo material. The resulting K_*x*_PMo material was highly crystalline with uniform and spherical morphology. It is denoted as K_*x*_PMo, where *x* denotes the amount of HPMo added to the initial mixture. B) A schematic representation of the DBT oxidation mechanism in the presence of H_2_O_2_ catalysed by K_*x*_PMo. DBT preferentially resides in the biphasic system's oil phase (*n*-octane), whereas the H_2_O_2_ oxidant and K_*x*_PMo catalyst primarily reside in the extractant phase (methanol). Therefore, the first step is to extract into the extractant phase to react with H_2_O_2_ in the presence of K_*x*_PMo.^289^

He *et al.* reported a series of Keggin-type K_*x*_[PMo^VI^_12_O_40_] (K_*x*_PMo, *x* = 1, 2, 3, 4) polyoxometalate salts prepared by hydrothermal synthesis using commercial F127 templates (Pluronic F127). The prepared K_*x*_PMo salts (Fig. 15A) were mesoporous with a high surface area (>40 m^2^ g^−1^) and could be successfully utilized for complete ODS of model oil in 1 h. By comparing the catalytic activity of the prepared POM salts, K_4_PMo showed the highest activity in the ODS process with a DBT removal rate of 99.5% within 60 minutes (Table S1 in SI, *k* = 0.076 min^−1^). A reaction mechanism of DBT oxidation by the K_4_PMo/H_2_O_2_ catalytic system has been proposed (Fig. 15B).^289^ In addition, the K_4_PMo catalyst also showed activity for the removal of other refractory sulfur compounds, DMDBT and BT, with removal efficiencies of 99.0% and 60.3%, respectively. The authors concluded that the ODS activity of K_*x*_PMo catalysts has a linear correlation with their electrochemically active surface area (ECSA). The higher activity of the K_4_Mo catalyst can therefore be attributed to its largest ECSA value, which shows that K_4_PMo exposes the largest number of anions [PMo^VI^_12_O_40_]^3−^ among all prepared catalysts. XRD structural analysis confirmed the good structural stability and successful recovery of the K_4_PMo catalyst that was used.^289^

**Fig. 16:**
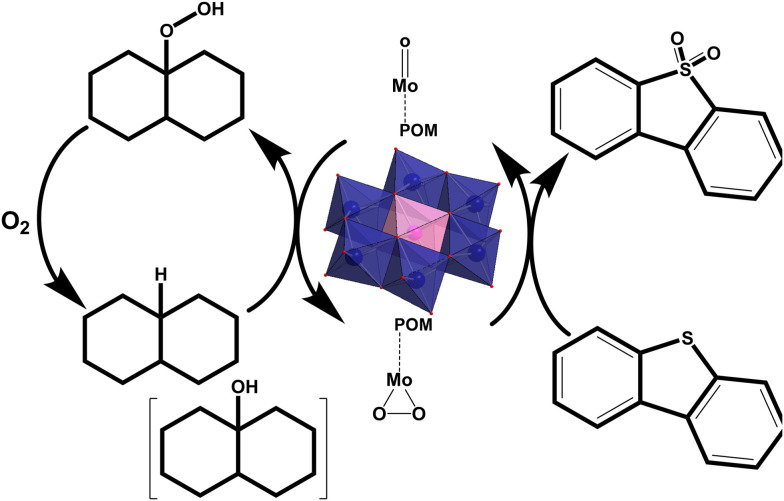
A schematic representation of DBT oxidation mechanism catalyzed by Anderson-type polyoxometalates ((NR_4_)_3_[X^III^Mo^VI^_6_O_24_H_6_] (X = Cr, Fe, Co; R = H or alkyl)) in the presence of O_2_ from air.^283^

Besides commonly utilized Keggin-type POMs, other archetypes, especially Anderson–Evans and Wells–Dawson, have also been used in the ODS process. Eseva *et al.* prepared a series of Anderson-type polyoxometalates (Fig. 16), (NR_4_)_3_[X^III^Mo^VI^_6_O_24_H_6_] (X^III^ = Cr, Fe, Co; R = H or alkyl), and tested their catalytic properties in the ODS process of model fuel. The Co(iii)-based Anderson type POM exhibited the highest catalytic activity in the desulfurization of model diesel with a 100% conversion rate of DBT within 60 minutes with a molar ratio of *n*(S) : *n*(cat.) = 50 : 1 (Table S1 in SI). By prolonging the reaction time to 120 min, 100% conversion was also achieved for BT. However, for 3-methylbenzene, only 59% conversion was achieved in 4 h.^283^ A reaction mechanism for DBT oxidation by the Co(iii)-POM has been proposed (Fig. 16). The crucial oxidation step in the catalytic system is based on the oxidation of a solvent (decalin), with the formation of an alkyl peroxide as the active species. Alkyl peroxide formation occurs by the reaction with an O_2_ molecule from the air in the presence of a Co(iii)-POM to form alkyl peroxides and the subsequent formation of the polyoxometalate's metal-dioxo species, as the source of active oxygen in the further oxidation of DBT. The quaternary ammonium cation in the (NR_4_)_3_[X^III^Mo^VI^_6_O_24_H_6_] catalyst structure allows the catalyst to adsorb the substrate molecules (DBT) and coordinate with the sulfur atom, after which the coordinated DBT is oxidized to a sulfone, thus simultaneously reducing (NR_4_)_3_[Co^III^Mo^VI^_6_O_24_H_6_] POM. The reduced form of (NR_4_)_3_[Co^III^Mo^VI^_6_O_24_H_6_] POM is re-oxidized with a new peroxide molecule, and a new catalytic cycle is started.^283^

**Fig. 17:**
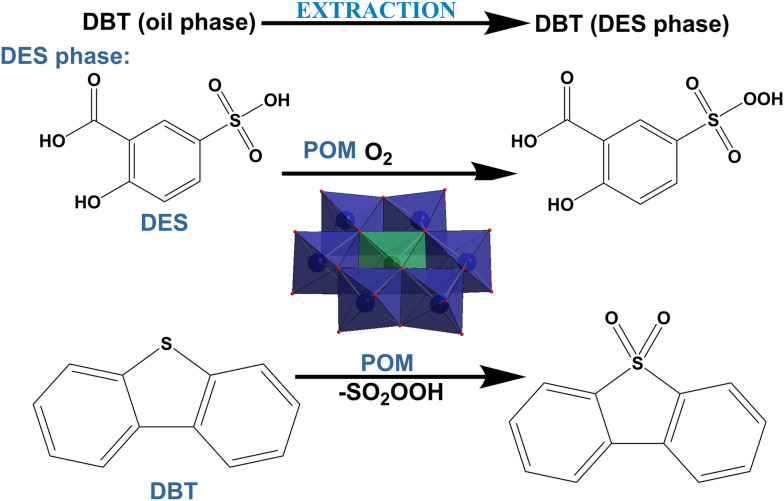
Schematic representation of the reaction mechanism for the oxidation desulfurization of DBT catalysed by coupling CrMo_6_ polyoxometalate with DESs under mild conditions (*T* = 60 °C).^284^

Hybrid POM-based materials have also been researched and have shown promising results as catalysts in ODS processes. Chi *et al.* reported the preparation of a new biomimetic catalytic system consisting of an Anderson-type POM ([Na_3_H_6_Cr^III^Mo^VI^_6_O_24_]) and deep eutectic solvents (DESs) and its successful application as a catalyst for the removal of sulfur compounds from both model and commercial diesel.^284^ Six different DESs (PEG/PAS, PEG/SSA, PEG/SA, PEG/DHBA, PEG/PXA and PEG/DL-MA) were combined with CrMo_6_ (Fig. 17), and their activity was tested. Only the addition of PEG/SSA, DES, containing an –SO_3_H group, resulted in 100% sulfur removal, while utilizing other DESs resulted in no higher than 30% sulfur removal.^284^ The desulfurization process followed the extraction–oxidation mechanism in which the POM and the DES acted as the electron transfer mediators and were both crucial for the process (Fig. 17).^284^

Ye *et al.* designed a new porous POM-based hybrid material by encapsulating a Keggin-type polyoxometalate [H_3_PW^VI^_12_O_40_] (PW) in the metal–organic framework UiO-66(Zr) and employed it as a catalyst in the ODS reaction of BT, DBT, and DMDBT at room temperature, with 98.2% DBT removal efficiency.^290^ A proposed reaction mechanism includes the extraction of DBT molecules from the model oil into the acetonitrile phase by the POM catalyst and H_2_O_2_. After extraction, DBT and H_2_O_2_ can be adsorbed into the catalyst pores, leading to the formation of ˙OH radicals *via* electron transfer from Zr–OH_2_ active centers in UiO-66 (Zr). Another H_2_O_2_ molecule can react with a W(vi) metal ion in the [H_3_PW^VI^_12_O_40_] POM to form the W(vi)-peroxo species that lead to the formation of O_2_˙^−^ radicals. Both O_2_˙^−^ and ˙OH radicals can oxidize DBT to DBTO_2_. The existence of two types of active centers in the catalyst, W(vi) in [H_3_PW^VI^_12_O_40_] and Zr–OH_2_ in UiO-66 (Zr), which forms two different active species, is probably responsible for the high efficiency of the catalyst in the ODS process.^290^

For the desulfurization of fossil fuels, Gao *et al.* prepared a series of Wells–Dawson-type POMs [H_6+*n*_P_2_Mo^VI^_18−*n*_V^V^_*n*_O_62_·*m*H_2_O] (*n* = 1–5; Mo_17_V_1_, Mo_16_V_2_, Mo_15_V_3_, Mo_14_V_4_, and Mo_13_V_5_), immobilized them on CNT carriers, and thereby prepared two different types of catalysts, CNT@PDDA@POM and POM@CNT.^291^ All prepared POM-based materials have shown to be catalytically active in the ODS process. CNT@PDDA@Mo_16_V_2_ showed the highest catalytic activity with 99.4% desulfurization efficiency. The better efficiency of this type of catalyst was due to a different POM position in CNT@PDDA@POM (on the surface of CNT@PDDA) compared to POM@CNT (deep in the CNTs' channel). Moreover, it was observed that the number of Mo centers replaced with V centers affects the efficiency, with a 16 : 2 ratio being the optimal Mo : V ratio for obtaining a high desulfurization activity of both catalysts. By combining CNT carriers with high mechanical properties, high thermal stability, and a high specific surface area, Gao *et al.* overcame disadvantages such as a low specific surface area and the difficulty of reclamation for pure POMs.^291^ More literature-known POM-based catalysts and their efficiency in the removal of refractory compounds from fossil fuels are summarized in Table S1 in the SI.

### 4.2 Removal of toxic gases – H_2_S, NO_*x*_ and SO_2_

#### 4.2.1 Hydrogen sulfide (H_2_S) in air pollution

Hydrogen sulfide is naturally present in crude petroleum, natural gas, volcanic gases, and geothermal sources. It is also a common by-product of many human activities, such as wastewater treatment,^292^ fossil fuel combustion,^54^ sewage treatment facilities,^55^ paper factories,^56^ food processing factories, and agriculture.^57^ Hydrogen sulfide is an odorous toxic gas with a corrosive nature and an adverse effect on human health and directly affects industrial production by reducing industrial catalysts' efficiency and causing equipment failure. It can also easily oxidize and form SO_2_ gas (eqn (1)), one of the leading causes of acid rain:^58^

1 H_2_S + 3/2O_2_ → SO_2_ + H_2_O

Furthermore, hydrogen sulfide readily reacts with metals, such as copper, and forms the corresponding sulfides (Cu_2_S) on the surface of electrical devices, causing electrical failures. H_2_S can also cause corrosion on surfaces, which can cause damage to buildings, for example, sewage plant facilities.^293^ In addition to SO_2_ (section 4.2.2), H_2_S can react with different compounds present in the atmosphere and form many other toxic by-products, such as carbonyl sulfides (eqn (2)), carbon disulfides (eqn (3)), sulfurous acid (eqn (4)), and PMs, that have been linked to ozone layer depletion:^294^

2 H_2_S + CO_2_ → COS + H_2_O

3 2H_2_S + CH_4_ → CS_2_ + 4H_2_

4 2H_2_S + 3O_2_ → 2H_2_SO_3_

Scientists and engineers have developed different methods for removing H_2_S from the environment, such as metal oxide oxidation,^295^ adsorption using different adsorbents (activated carbon or wet scrubbing),^296^ the Claus process,^297^ biofiltration, oxidative desulfurization, and the LRSR process.^298^ The latter two methods are recently the most commonly used methods with a very high desulfurization capacity and efficient production of elemental sulfur using various redox mediators (*e.g.*, Fe(iii)/Fe(ii)).^298^ Such mediators have shown outstanding results, but they are still mostly chemically unstable and require low pH, which is unfavorable for H_2_S removal processes.

POMs and different POM-based hybrid materials have shown high efficiency in H_2_S removal due to their redox properties and structural stability. For the regeneration of these POM-based catalysts, a redox-mediated electrochemical regeneration method using oxidants such as H_2_O_2_ or O_2_ has recently been shown to be effective.^280^

A purely inorganic POM was applied by Pei *et al.* who successfully synthesized a set of rare-earth Dawson-type polyoxometalates (K_17_[Pr^III^(P_2_Mo^VI^_17_O_61_)_2_] (PrPMo), K_17_[Gd^III^(P_2_Mo^VI^_17_O_61_)_2_] (GdPMo), K_17_[Sm^III^(P_2_Mo^VI^_17_O_61_)_2_] (SmPMo) and K_17_[Eu^III^(P_2_Mo^VI^_17_O_61_)_2_] (EuPMo)) and utilized them in the removal of H_2_S. Due to the excellent redox properties of Ln(iii)-doped POMs, the influence of different Ln(iii) species on H_2_S removal was investigated. From the experimental results, the prepared compounds were ranked according to their efficiency for the removal of H_2_S in the following order: PrPMo (90%) > EuPMo (88%) > SmPMo (87%) > GdPMo (85%). The PrPMo polyoxometalate showed the best desulfurization and regeneration properties with 90% efficiency at 25 °C within 400 min. The XPS spectral analysis showed that H_2_S is first oxidized to S by a redox reaction with PrPMo, in which Mo(vi) is simultaneously reduced to Mo(iv). During the electrochemical regeneration of PrPMo, S is further oxidized to SO_4_^2−^ as the main desulfurization product, and Pr(iv) is reduced to Pr(iii) during the regeneration process. The results of repeated XPS measurements confirmed the successful regeneration of PrPMo.^116^

Ma *et al.* described a new approach for an H_2_S oxidation and sulfur recovery system using the hybrid POM-based hybrid materials, [C_4_mim]_3_PMo^VI^_12_O_40_-ILs ([C_4_mim]^+^ = 1-butyl-3-methylimidazolium cation), where they investigated the influence of several different [C_4_mim]^+^-based ionic liquids (ILs), [C_4_mim]Cl, [C_4_mim]BF_4_, [C_4_mim]PF_6_ and [C_4_mim]NTf_2_. Of all the POM-IL systems tested, the [C_4_mim]_3_PMo^VI^_12_O_40_-[C_4_mim]Cl system has shown to be the most effective for removing H_2_S, with 100% efficiency. The adsorption mechanism of H_2_S desulfurization is explained by the theory of cavities and the strong interaction between H_2_S and Cl^−^. Additionally, they confirmed that the POM-IL material could be successfully recovered more than six times without losing its efficiency.^299^

**Fig. 18:**
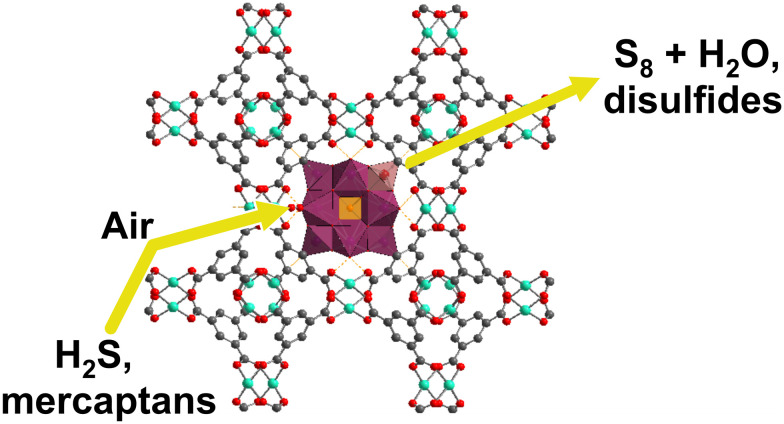
Crystal structure of POM–MOF ([{(CH_3_)_4_N}_4_CuPW_11_O_39_H]) material. The [CuPW_11_O_39_]^5−^ polyhedra are orientationally disordered into the pores. It was concluded that the catalytic decomposition of H_2_S was taking place inside the pores.^300^

Song *et al.* prepared a POM-based metal–organic framework [{(CH_3_)_4_N}_4_CuPW^VI^_11_O_39_H] (POM–MOF) hybrid material (Fig. 18) by combining a Keggin-type polyoxometalate [CuPW^VI^_11_O_39_]^5−^ and MOF-199.^300^ The POM–MOF/O_2_ catalytic system effectively oxidizes H_2_S to solid S_8_ with up to 95% H_2_S removal efficiency. Additionally, it has been shown that the POM–MOF system can successfully oxidize mercaptans to disulfides. The POM–MOF catalyst can be successfully reused in the oxidation process after simple filtration, washing, and drying. The UV-vis and FT-IR spectra showed that the [CuPW^VI^_11_O_39_]^5−^ structure was preserved in the POM–MOF catalyst at pH 11 for at least 12 h. The POM–MOF hybrid material showed better stability and pH resistance than the [CuPW^VI^_11_O_39_]^5−^ POM alone.^300^

A summary of literature-reported POMs and POM-based hybrid materials and their efficiencies in H_2_S removal are given in Table 2 at the end of section 4.

#### 4.2.2 Nitrogen oxides (NO_*x*_) and sulfur dioxide SO_2_ in air pollution

Interest in NO_*x*_ emissions and their regulation began after 1952 with the confirmation of their role in the formation of photochemical smog.^301^ Several different nitrogen oxides are present in the atmosphere, *e.g.*, N_2_O, NO, NO_2_, N_2_O_3_, N_2_O_4_, NO_3_, and N_2_O_5_. However, NO_*x*_ mainly refers to NO and NO_2_ oxides because nitrogen oxides are primarily released into the environment in these forms, and NOx emissions contain 95% NO and 5% NO_2_.^302^ NO is considered less toxic than NO_2_ and can cause eye irritation, but NO_2_, even at low concentrations, can cause acute lung injury with pneumonitis^303^ and fulminant pulmonary edema.^304^ In urban areas where a higher concentration of NO_2_ gas present, many respiratory and cardiovascular diseases and even increased mortality among the exposed population have been observed.^303,305^

Moreover, H_2_S and NO_*x*_ gases are considered to be among the major air pollutants because they are thought to be responsible for various environmental issues, such as photochemical smog, acid rain,^306^ tropospheric ozone,^307^ ozone layer depletion, and even global warming, as a result of N_2_O.^308,309^ NO_*x*_ gases are also associated with the greenhouse effect, and in the higher layers of the atmosphere, they can react with various compounds present there (O_3_, VOCs, *etc.*), leading to ozone depletion. Most air pollution occurs and remains within the lowest layer of the atmosphere, the troposphere. NO_*x*_ gases can lead to the formation of tropospheric ozone after photochemical degradation to NO (eqn (5)):

5 NO_2_ + *hv* (*λ* < 440 nm) → NO + O

6 O + O_2_ → O_3_ (troposphere)

With NO not absorbing radiation above 230 nm and thus not acting as an inhibitor in the lower atmosphere, the resulting atomic oxygen reacts with O_2_ in the troposphere to form ozone (eqn (6)), leading to the tropospheric ozone formation.^301,307^ Great efforts have been made to develop methods for removing NO_*x*_ from the atmosphere in the last few decades.^306,310,311^ Adsorptive–desorption methods^307,309^ and Fenton-like reactions,^285^ as examples of AOPs, have been extensively studied for the removal of NO_*x*_ and SO_2_ gases. The Fenton-like oxidation process consists of oxidation and degradation of different pollutants in the presence of a catalyst and H_2_O_2_ as an oxidant activated by UV-light irradiation.^280,311^

In the oxidation process, the generated reactive ˙OH radicals (eqn (7)) oxidize a wide range of different substrates. Such radical-assisted oxidation processes have been shown to be particularly effective in removing organic dyes, phenols, antibiotics, and insecticides from wastewater and are a popular research topic for pollution removal applications.^212^

7 H_2_O_2_ + *hv*(cat.) → 2˙OH

Zhao *et al.*^312^ reported the synthesis of an iron-substituted Keggin-type polyoxometalate-based catalyst Fe^III^AspPW from ferric chloride (FeCl_3_), aspartic acid (Asp), and phosphotungstic acid ([H_3_PW^VI^_12_O_40_]). The Fe^III^AspPW was used to activate H_2_O_2_ to form active ˙OH species, which are crucial for the removal of NO from flue gas. The proposed catalytic mechanism consists of two redox cycles that occur on the surface of the Fe^III^AspPW catalyst: the redox cycles of Fe^III^ ↔ Fe^II^ and POM ↔ POM^−^. In the Fenton-like process, first, in the redox cycle of Fe^III^ ↔ Fe^II^, Fe^3+^ reacts with H_2_O_2_ to first form HOO˙ (eqn (8)) and then ˙OH (eqn (9)) active species:

8 Fe^3+^ + H_2_O_2_ → Fe^2+^ + H^+^ + HOO˙

9 Fe^2+^ + H_2_O_2_ → Fe^3+^ + OH^−^ + ˙OH

10 Fe^3+^ + HOO˙ → Fe^2+^ + H^+^ + O_2_

11 Fe^2+^ + HOO˙ + H^+^ → Fe^3+^ + H_2_O_2_ (closing the cycle)

In the POM ↔ POM^−^ redox cycle, the POM component is firstly reduced to the POM^−^ form in a reversible reaction, and then the reduced POM^−^ form further reacts with H_2_O_2_ to form active ˙OH species (eqn (13)). In addition, to close the redox cycle, POM^−^ is oxidized by O_2_ or O_2_˙:

12 Fe^2+^ + POM ⇄ POM^−^ + Fe^3+^

13 POM^−^ + H_2_O_2_ + H^+^ → POM + H_2_O + ˙OH

14 POM^−^ + O_2_ → POM + O_2_˙^−^

15 POM^−^ + O_2_˙^−^ + 2H^+^ → POM + H_2_O_2_

This catalytic system showed great activity for removing NO with 94.6% efficiency.^312^ Moreover, Liu *et al.* showed that the Fe^III^AspPW/H_2_O_2_ catalytic system could also be used to simultaneously remove SO_2_ and NO from flue gas in a UV-Fenton-like process with efficiencies of the Fe^III^AspPW catalyst of 100% for SO_2_ removal and 84.27% for NO removal.^313^ Wang *et al.* presented a series of Ge(iv)-based Keggin-type polyoxometalates (([H_4_GeW^VI^_12_O_40_] (HGeW), [H_5_GeW^VI^_11_V^V^O_40_] (HGeWV), [H_5_GeMo^VI^_11_V^V^O_40_] (HGeMoV), [H_5_GeW^VI^_9_Mo^VI^_2_V^V^O_40_] (HGeWMoV)) and utilized them in the removal of NO_*x*_ pollutants.^314^ The adsorption–desorption experiments showed the following adsorption efficiencies for the removal of NO_*x*_ gases: HGeW 81.5%) > HGeWV (74%) > HGeWMoV (67%) > HGeMoV (52%). The Keggin-type polyoxometalate HGeW (Fig. 2E) showed the highest NO_*x*_ removal activity with 81.5% removal and 68.3% N_2_ selectivity, of which 65% was from fractionated NO and 35% NO_2_ gas. Additionally, the H_2_S removal efficiency of HGeW was compared with that of the parent Keggin [H_3_PW^VI^_12_O_40_] (HPW) polyoxometalate (54.1% efficiency). The FT-IR studies revealed that NO_*x*_ is adsorbed on HGeW mainly in the form of NOH^+^ and NO˙ species, but on the HPW, only NOH^+^ is observed as the main form during adsorption. Moreover, TPD-MS experiments were carried out to investigate the further decomposition mechanism of NO_*x*_ over HGeW and HPW. The TPD-MS analysis showed that while the decomposition products (NO, N_2_O, N_2_, and O_2_) appear in the same order for both HPW and HGeW, they appear at different temperatures, lower in the case of HPW. The NO species appeared at the lowest temperature for both NO_*x*_ decomposition experiments. It is believed that a significant part of the NO_*x*_ is physically adsorbed onto HPW and HGeW in the form of NO at a lower temperature. Meanwhile, the later appearing N_2_O could be a product of the disproportionation reaction of NO in which N_2_ is formed because of the bonding effect of N-atom, which comes from N–O bond breakage. The difference in NO_*x*_ removal efficiency and N_2_ selectivity between HPW and HGeW could be due to the HGeW's ability to intensively loosen the N–O bond, resulting in easier NO_*x*_ decomposition, and by better NO_*x*_ adsorption for HGeW in the form of both NO˙ and NOH^+^. It is believed that the presence of the Ge(iv) atom instead of P as the central atom plays a significant role in the processes described above.^314^

### 4.3 Volatile organic compounds in air pollution (VOCs)

#### 4.3.1 Removal of volatile organic compounds – refractory BETX compounds (benzene, ethylbenzene, toluene, and xylenes)

VOCs are a group of liquid organic compounds that can easily evaporate at room temperature. In addition to their volatility, this group of compounds has variable lipophilicity, small molecular size, and are uncharged, resulting in inhalation as the primary route of human exposure.^315^ VOCs are classified according to molecular structure and functional groups and include aliphatic hydrocarbons, aromatic hydrocarbons, alcohols, ethers, esters, aldehydes, *etc.* Due to their properties and wide application in different areas of everyday life, they are common indoor and outdoor air pollutants.^285,315^ As outdoor pollutants, they result from the development of industry and urbanization, which involves the increased use of fossil fuels in transport, industrial production, and wastewater treatment plants. As indoor air pollutants, VOCs are found in tobacco smoke, various air fresheners and perfumes, paints and coatings, cleaning products, *etc.*, and can be harmful to human health at excessive concentrations.^285,286,315^ Especially, the group of so-called refractory BETX compounds, which stands for benzene, ethylbenzene, toluene, and xylenes, is problematic due to their high toxicity and confirmed carcinogenic nature.^285,315^ Besides being confirmed carcinogens, depending on the concentration and length of exposure, various consequences of VOCs exposure have been reported: eye and respiratory tract irritation, headache, dizziness, allergic skin reaction, fatigue, memory impairment, loss of consciousness, and even death.^286,315,316^

Various methods^285^ have been studied in search of an efficient and affordable method for removing volatile organic compounds (VOCs) from the air, such as condensation, adsorption,^317,318^ and (photo)catalytic oxidation.^316^ Photocatalytic oxidation (PCO) is a promising method for removing VOCs from the air, and so far, TiO_2_-based photocatalytic oxidation^319^ has mainly been investigated. Due to the tendency to develop a sunlight/visible-light-driven method, TiO_2_ has been shown to be a non-ideal photocatalyst due to its poor solar energy utilization.^320^ Therefore, there is a need to design new materials that could be successfully applied as photocatalysts for VOCs' photocatalytic oxidation.^315,321^

**Fig. 19:**
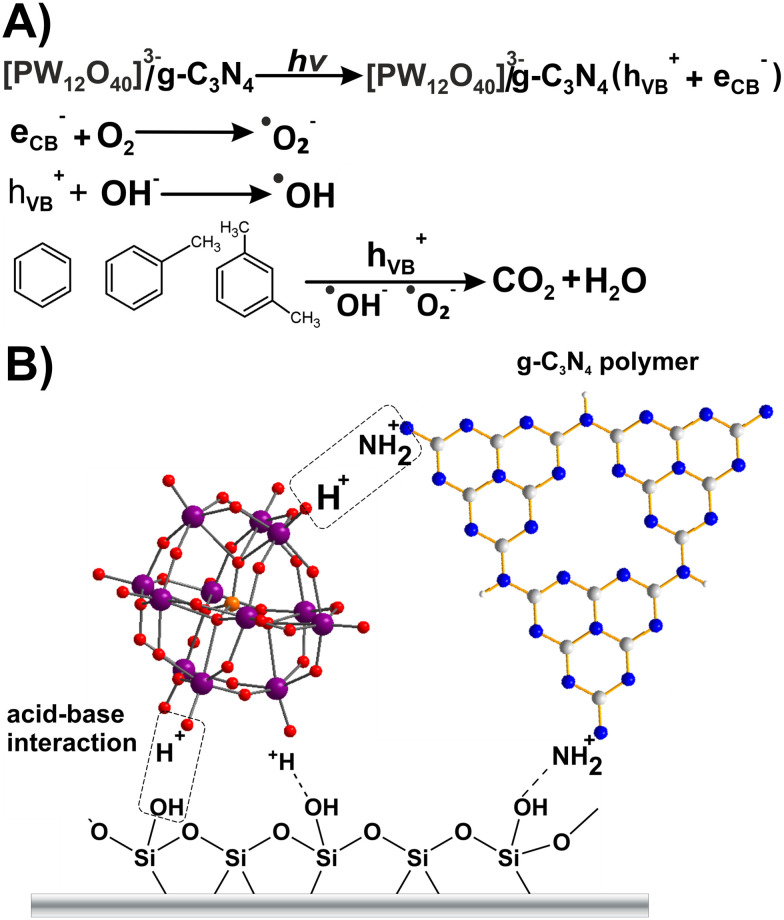
A) Reaction mechanism of photocatalytic oxidation of VOCs catalysed by PW_12_/g-C_3_N_4_ films. B) Schematic representation of the preparation of PW_12_/g-C_3_N_4_ catalyst and its framework structure.^286^

Meng *et al.* have shown that photoactive PW_12_/g-C_3_N_4_ optical films (Fig. 19B) can be obtained by combining the Keggin-type POM, [H_3_PW^VI^_12_O_40_], with polymeric graphitic carbon nitride (g-C_3_N_4_) and then successfully utilized them as photocatalysts for the efficient removal of benzene, toluene, and *m*-xylene. The PW_12_/g-C_3_N_4_ optical films showed excellent removal efficiencies for benzene (90.3%), toluene (100%) and *m*-xylene (97.5%). They also demonstrated excellent stability and reusability for up to 30 cycles without signs of activity loss. The results of DMPO spin-trapping ESR measurements indicated that the PW_12_/g-C_3_N_4_ films follow a simulated sunlight-driven direct Z-scheme-dictated charge carrier transformation mechanism that accelerates interfacial charge carrier separation and the formation of O_2_^−^˙ and HO˙ radicals that are involved in VOCs oxidation. In the suggested mechanism (Fig. 19A), charge separation and formation of e^−^_CB_–h^+^_VB_ pair occur (photocurrent), resulting in the formation of O_2_^−^˙ and HO˙ active species that directly participate in the complete mineralization of VOCs to CO_2_ and H_2_O (Fig. 19A).^286^ Also, Gamelas *et al.* presented a series of new cellulose/silica hybrid composites functionalized with different Keggin-type POMs ([PV^V^_2_Mo^VI^_10_O_40_]^5−^, [PV^V^Mo^VI^_11_O_40_]^4−^, [PMo^VI^_12_O_40_]^3−^ and [PW^VI^_12_O_40_]^3−^) and investigated their potential application in the catalytic oxidation of VOCs present in urban air.^281^ The new cellulose/silica hybrid materials were composed of approximately 56 wt% of polysaccharides, *ca.* 37 wt% of propylamine-modified silica, 2 wt% of POM, and 5 wt% of hydration water. Catalytic activity experiments were performed by pumping polluted air through Teflon tubes filled with the catalysts and then analyzing the treated air by GC-chromatography.

The catalytic activity of the new POM-based hybrid material for VOCs oxidation was visible as a change in the color of the material from yellow to green, indicating the occurrence of V(v) → V(iv) reduction in the POM. The GC-chromatography of a real air sample treated with the new hybrid material indicated complete oxidation of most C_5_–C_11_ volatile organic compounds. The successful recovery of the used catalyst was achieved by passing purified air through the Teflon tubes filled with used catalyst, which was noticeable by the color change of the material from green to yellow.^281^

POMs have also proven as suitable adsorbents for adsorption techniques to remove VOCs from the air. Ma *et al.* reported a newly synthesized POM/MOF hybrid material, K_2_[Cu_12_(BTC)_8_·12H_2_O][HPW^VI^_12_O_40_]·28H_2_O or NENU-28 and its possible application as an adsorbent for the adsorption of VOCs, including short-chain alcohols (MeOH and EtOH), cyclohexane, benzene, and toluene.^322^ The adsorption capacity of NENU-28 for methanol, ethanol, 1-propanol, 2-propanol, cyclohexane, benzene and toluene was tested in VOCs adsorption experiments. The adsorption amount of MeOH for NENU-28 is 6.70 mmol g^−1^ which corresponds to the adsorption of 37.52 molecules of MeOH per catalyst formula unit. Comparison with the initial MOF (Cu_3_(BTC)_2_), which can adsorb 5.14 mmol g^−1^ methanol (14.36 MeOH molecules per formula unit), shows that POM-functionalized MOFs bring a significant improvement in the adsorption capacity for MeOH. The NENU-28 hybrid material also showed an increase in the amount of adsorbed EtOH (4.78 mmol g^−1^ or 26.77 molecules of EtOH per formula unit) compared to Cu_3_(BTC)_2_ (3.54 mmol g^−1^ or 9.89 molecules of EtOH per formula unit). Although the mechanistic details are not fully understood yet, the results indicate that the presence of the Keggin-type POM [HPW_12_O_40_] in the NENU-28 has a favorable effect on the adsorption properties of the POM–MOF material.^322^

#### 4.3.2 Removal of aldehydes

Aldehydes, especially formaldehyde and acetaldehyde, are the most common VOCs present in the air as indoor air pollutants.^323^ The primary sources of these air pollutants come from building materials, varnishes, and paints, flooring, and furniture materials. Formaldehyde and acetaldehyde are classified as group 1 carcinogens and are therefore proven harmful to human health.^323,324^ Several approaches have been developed to reduce their concentration. They can be divided into passive (*e.g.*, better ventilation, using formaldehyde-free materials) and active (*e.g.*, removal techniques – adsorption and catalytic oxidation) approaches.^323,324^ In this section, the focus will be on the development of different active approaches for the removal of aldehydes.

**Fig. 20:**
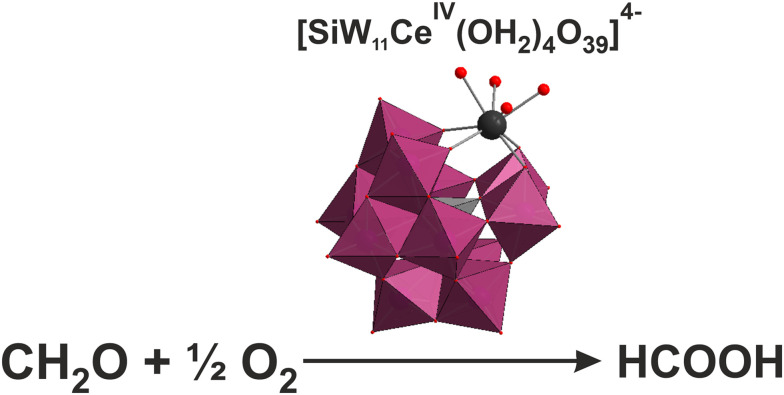
Aerobic oxidation of formaldehyde to formic acid catalysed by Ce-containing Keggin-type POM (NaH_3_[SiW_11_Ce^IV^O_39_]) under mild conditions (air, *T* = 25 °C).^278^

[H_4_SiW^VI^_12_O_40_] and [K_8_SiW^VI^_11_O_39_] (0% efficiency). Kholdeeva *et al.* developed a new Ce-containing polyoxometalate NaH_3_[SiW^VI^_11_Ce^IV^O_39_] (Ce-POM; Fig. 20)^278^ and its dimer in the solid-state, and tested their promising efficiency in the removal of formaldehyde (CH_2_O) under mild conditions (20–40 °C). Although the reaction mechanism itself is complex and involves CH_2_O autooxidation, the Haber–Weiss radical-chain process,^325^ and product formation inhibition, the reaction stoichiometry itself satisfies the equation in Fig. 20. The efficiency of an unoptimized oxidation process of CH_2_O in the presence of Ce-POM/O_2_ (efficiency 25%) was compared to the oxidation of CH_2_O in the presence of Ce(SO_4_)_2_ (efficiency 9%) and in the presence of two POMs without Ce(iv) metal atom. The results of these efficiency comparisons suggested that the activity of the Ce-POM catalyst could be attributed to the synergistic action of the POM and Ce(iv). By optimizing the reaction conditions (adding a small amount of H_2_O_2_), the conversion efficiency of CH_2_O increased from 25% to 85% with a yield of 66% HCOOH in the presence of NaH_3_[SiW^VI^_11_Ce^IV^O_39_].^278^

Gamelas *et al.* successfully immobilized the α-isomer of the polyoxometalate [SiW^VI^_9_O_37_Ru^III^_3_(H_2_O)_3_Cl_3_]^7−^ (Ru-POM) onto a CSH support, obtaining a heterogeneous catalyst Ru-POM-CSH that was active in formaldehyde oxidation.^326^ Oxidation of CH_2_O was performed at room temperature by flushing an air/formaldehyde gas mixture through a Teflon tube filled with Ru-POM-CSH catalyst or only the CSH carrier without POM. Initially, the CH_2_O degradation results for the first two cycles did not differ significantly between CSH and Ru-POM-CSH. This lack of degradation increase could be explained by chemisorption and the reaction between the amino groups of the CSH carrier and CH_2_O:

16 Si(O)_3_–(CH_2_)_3_–NH_2_ + CH_2_O → Si(O)_3_–(CH_2_)_3_–N=CH_2_ + H_2_O

After the second cycle, the efficiency of CSH in the removal of CH_2_O dropped sharply. By the 4th cycle, it was 0%, which indicates the simple saturation of the CSH carrier. When Ru-POM-CSH was used as a catalyst, efficiency decreased more slowly, with about an 8% decrease between cycles after the 5th cycle. No catalyst saturation was observed, which can be attributed to the oxidation of CH_2_O catalyzed by Ru-POM. After passing purified air through a Teflon tube containing Ru-POM-CSH material, unlike CSH alone, the material was successfully regenerated. Product analysis revealed that CO_2_ and H_2_O were the main reaction products formed by catalytic oxidation of CH_2_O in the presence of Ru-POM-CSH. These results indicate that the reaction undergoes a predominantly non-radical mechanism because the final product would be formic acid and carbon monoxide in the case of a radical mechanism.^326^

The following mechanism of a CH_2_O oxidation reaction in the presence of Ru-POM-CSH was proposed:

17 POM–Ru^III^_3_(H_2_O) + CH_2_O ⇄ POM–Ru^III^_3_(CH_2_O) + H_2_O

18 POM–Ru^III^_3_(CH_2_O) → HPOM–Ru^III^_2_Ru^II^ + ˙CHO

The initial step probably involves oxidation of the substrate (CH_2_O) by a catalyst through ligand replacement, binding of O_2_ to the partially reduced catalyst (eqn (19)), and its activation and further reaction with ˙CHO:

19 HPOM–Ru^III^_2_Ru^II^ + O_2_ ↔ HPOM–Ru^III^_2_Ru^II^–O_2_ ↔ HPOM–Ru^III^_2_Ru^III^–O_2_˙

20 HPOM–Ru^III^_2_Ru^III^–O_2_˙ + ˙CHO → POM–Ru^III^_3_ + CO_2_ + H_2_O

The oxidation reaction of CH_2_O with Ru-POM-CSH can be summarized as follows:^325^

21 CH_2_O + O_2_ → CO_2_ + H_2_O

Kholdeeva *et al.* also synthesized tetra-*n*-butylammonium (TBA) salts of Co-substituted Keggin-type polyoxometalates [TBA_4_HPW^VI^_11_CoO_39_] (I) and [TBA_5_PW^VI^_11_CoO_39_] (II) (Co-POM) and immobilized them onto both NH_2_^−^ and NH_3_^+^ modified mesoporous silica surfaces.^282^

The catalytic activity of the solid Co-POM materials (I) and (II) was tested for the oxidation of isobutyraldehyde (IBA) and compared with the activity of the homogeneous Co-POM salts (I) and (II). The results showed that the IBA conversion rate in MeCN under mild conditions (1 atm of air, *T* = 20–40 °C) without a catalyst was 28%. In the presence of only the NH_2_-modified mesoporous silica support, the IBA conversion rate was only 6%, indicating that the NH_2_^−^ silica support is an inhibitor of the IBA oxidation. When one of the solid Co-POM catalysts, [TBA_4_HPW^VI^_11_CoO_39_] (I) or the non-protonated [TBA_5_PW^VI^_11_CoO_39_] (II), (immobilized on NH_2_^−^ or NH_3_^+^-silica support) was added to the reaction mixture, the IBA oxidation to IBAc continued at room temperature. The protonated salt [TBA_4_HPW^VI^_11_CoO_39_] (I) had a higher redox potential and better catalytic activity for IBA oxidation than the non-protonated salt (II). The catalytic activity of the immobilized Co-POM (I) and the homogeneous salt (I) exhibited similar catalytic performance (92% IBA conversion) for the first two cycles. However, after the third cycle, the immobilized Co-POM (I) catalyst lost up to 15% of its activity due to Co-POM leaching, showing that the homogeneous Co-POM (I) salt had better long-term stability.^282^

All literature-known polyoxometalates and their applications in removing aldehydes are summarized in Table 2.

### 4.4 Summary of POM-based technologies in air purification

Various POMs alone and combined with MOFs, CNTs, and mesoporous silica supports show promising results for removing air pollutants including refractory sulfur compounds from fossil fuels (section 4.1), toxic gases like H_2_S (section 4.2.1), NO_*x*_/SO_2_ (section 4.2.2), and carcinogenic VOCs (section 4.3) in indoor/outdoor air. Keggin-type POM structures dominate oxidative desulfurization of fossil fuels under mild conditions (∼85% of literature; Table S1), outperforming traditional HDS processes and avoiding high pressures/temperatures while meeting ultra-low sulfur regulations. Anderson–Evans POMs enable efficient desulfurization through alkyl peroxide mechanisms with extended lifetimes, while lanthanide-doped Wells–Dawson POMs exhibit superior H_2_S oxidation and stability for NO_*x*_/SO_2_ removal (Table 2).

POM-based hybrid materials further enhance performance, such as K_4_PMo mesoporous salts for rapid DBT removal (Table S1), PW_12_/g-C_3_N_4_ films mineralizing BETX VOCs under visible light *via* Z-scheme mechanism, and POM–MOFs like NENU-28 boosting VOC adsorption (section 4.3). Ce- and Ru-containing Keggin POMs catalyze aldehyde oxidation to CO_2_/H_2_O at room temperature, with Ru-POM-CSH showing sustained activity over cycles without saturation (Table 2). Structural diversity tailors redox properties and active oxygen species (˙OH, O_2_˙^−^), addressing key air pollutants effectively.

## 5 Polyoxometalates in sensor applications

**Fig. 21:**
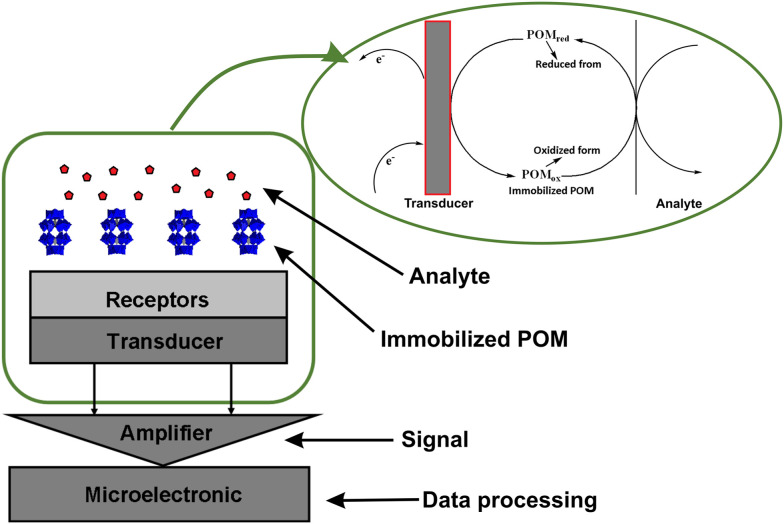
Schematic representation of POM-based electrochemical sensors.^349^

Immobilization of POMs on the different supporting surfaces facilitates their electrochemical properties for sensor applications.^349^ Numerous methods, such as chemical adsorption,^350,351^ electrodeposition,^352,353^ encapsulation,^354^ the Langmuir–Blodgett process,^355,356^ and layer-by-layer deposition,^357,358^ have been used to deposit POMs on electrodes to form monolayer or multilayer structures.^351^ As can be seen in Fig. 21, POM-based sensors are used as the analytical unit, in which the POM is immobilized onto a solid substrate utilized as a transducer. If the POM has been successfully immobilized onto the transducer while preserving its structural integrity, the POM part of the sensor should be able to recognize and catalyze the analyte *via* an induced chemical reaction followed by the transformation of the chemical reaction energy into an electrical signal. The electrical signal is later amplified and converted by signal processing equipment into a display.^349^ The POM-based sensors, like other sensors, show all main characteristics such as sensitivity, selectivity, linear range, response time, detection limit, and stability.^359^ The most critical properties of most POM-based sensors are selectivity and response rate, and often, they are not addressed by authors. For sensors to have high selectivity, the sensor should have a heightened response to a substrate but an inadequate response to interferences. Recently, it has been shown that these issues could be solved by combining the POMs with organic moieties or CNTs with the addition of noble metal NPs. Generally, the POM-based sensors showed good selectivity and low response time while being stable and active at neutral pH.^360,361^

POM-based sensors operate through a synergistic mechanism that involves redox-driven signal transduction, coordination-induced structural alterations, and catalytic amplification processes. This enables the highly sensitive detection of various chemical and biological analytes. The multi-electron redox functionality of POM clusters allows them to undergo reversible changes in oxidation state upon interaction with target species, resulting in measurable outputs that can be electrochemical, optical, or conductometric. In the realm of electrochemical sensing, POMs facilitate rapid electron transfer at the electrode–analyte interface, a process that can be enhanced through their incorporation into conductive matrices or nanostructured supports, thereby optimizing charge-transfer kinetics and reducing detection limits.^362^ Optical sensors utilize intervalence charge-transfer transitions or ligand-to-metal charge-transfer phenomena that occur when analytes interact with or reduce the POM framework, resulting in observable shifts in absorbance or luminescence.^363^ Furthermore, the catalytic sensing mechanisms exploit the inherent oxidative or reductive catalytic properties of POMs, where reactions initiated by the analyte generate amplified signals under controlled conditions such as specific pH levels, ionic strength adjustments, or the presence of co-substrates.^364^ The overall performance of these sensors is heavily influenced by various experimental factors, including the speciation of POMs, electrode modification strategies, solvent polarity, and the stability range of the POM in the working environment. Consequently, methodological optimization becomes vital for achieving selectivity, reproducibility, and reliability in practical applications.^365^

### 5.1 POM-based sensors in the detection of water pollution

The POM-based sensors have already explored various analyte classes dispersed in either the gas or liquid phase. The electrocatalytic reduction of nitrate, iodate, bromate, nitrite, and hydrogen peroxide by POMs immobilized on a substrate was carried out for sensing applications. Starting with stable Keggin and Dawson type POMs ([H_3_PMo^VI^_12_O_40_], [H_6_P_2_Mo^VI^_18_O_62_·*n*H_2_O], [H_3_PW^VI^_12_O_40_], [P_2_W^VI^_18_O_62_]^6−^, and α-[H_4_SiMo^VI^_12_O_40_]) are extensively explored as electrochemical sensors.^361,366–369^ Though the sensors showed prominent sensitivity and wide linear range, they operated at a low pH (pH < 2) to stabilize the POM architecture.^370^ In 2012, Ma *et al.*^371^ synthesized a layer-by-layer composite film using palladium nanoparticles and a Dawson-type POM ([K_7_P_2_W^VI^_17_O_61_(FeOH_2_)·8H_2_O], (P_2_W^VI^_17_Fe)) to determine the electrolytic behavior towards the oxidation of hydrazine sulfate (N_2_H_4_SO_4_) and reduction of hydrogen peroxide. The H_2_O_2_ exhibits sensitivity, detection limit, and linear concentration in the range of 66.7 μA mM^−1^, 1 μM (S/N = 3), 1.5 μM to 3.9 mM, respectively. Likewise, N_2_H_4_SO_4_ displays the same parameter in the range of 0.2 μA mM^−1^, 1.5 μM (S/N = 3), 2 μM to 3.4 mM, respectively, with sensing response time around 4 s.^371^ Furthermore, Zhu *et al.*^372^ synthesized four Preyssler-type POM-based organic–inorganic crystals to effectively detect non-enzymatic H_2_O_2_. The compounds exhibit the lowest detection limit of 0.13 mM with a high sensitivity of 4.35 μA mM^−1^ and a response time of 1 s.^372^ Ag-doped MoO_3_ immobilized on the graphene-like carbon nitride (C_3_N_4_) was first prepared and employed as an electrochemical sensor by Zhao *et al.*^373^ to detect H_2_O_2_. Herein, [Ag_6_Mo^VI^_7_O_24_]/Ag-MOF precursor was used to synthesize the nanoporous structure resulting in a linear detection range of 0.25 μM–0.43 mM towards H_2_O_2_ owing to its efficient electrocatalytic property.^373^ Additionally, isopolymolybdate-based compounds are explored as photoelectric sensors for detecting inorganic ions (*e.g.*, Cr(vi), Hg^2+^, NO_2_^−^).^374^ Additionally, complex POM structures (*e.g.*, pyrazole derivative Keggin ions,^375^ 3D coordination polymers doped with Keggin POM^376^ or hourglass-type POM crystals^377^) have been explored as the active electrode for the acute and faster sensing of bromate, nitrate, and heavy, metal ions.

### 5.2 POM-based sensors in the detection of air pollution

Krutovertsev *et al.* first addressed POM-based gas sensors by employing various Wells–Dawson type POMs doped with polyaniline to detect ammonia gas.^378^ POM-doped conducting polymer film is ideal for gas sensing as POMs react with the gas, and conducting polymer substrate converts that into an electrical signal. The recognition of other hazardous gases, such as NO_*x*_, CO, and the vapors of organic solvents, can also be determined because the proton-conducting POMs enhance the material's selectivity and sensitivity.^379,380^ Ammam *et al.*^381^ recently reported a sensitive and selective NO_*x*_ gas sensor using the [K_6_P_2_Mo^VI^_18_O_62_·H_2_O] POM and polypyrrole (PPy), exhibiting extended linearities (up to 5500 ppm NO_*x*_). Although all so far mentioned POM-modified electrodes shows catalytic properties and can recognize the analyte, not all can be employed as sensors. In order to achieve a high-performance sensor, the modified electrode should fulfill the conditions of molecular recognition between POMs and specific analytes.^381^

A high-performance gas sensor was developed by Wang *et al.*^382^ by using heteropolytungstate (HPT) doped SnO_2_ nanorods [HPT abbreviation as (C_4_H_10_ON)_23_[HN(CH_2_CH_2_OH)_3_]_10_H_2_[Fe^III^(CN)_6_(α_2_-P_2_W^VI^_17_O_61_Co^II^)_4_]·27H_2_O]·SnO_2_/HPT composite film, which demonstrated higher photoconductivity than pristine SnO_2_ and revealed improved gas sensing for the methylbenzene and formaldehyde at room temperature (25 °C). Electron–hole recombination in the composite was retarded due to the photo-induced transfer of an electron from SnO_2_ to HPT. An n-type semiconductor material BiVO_4_ loaded with different POMs, was exploited as a photo-anode for photoelectrochemical gas sensing capability for NO_2_.^382^ Among different Keggin type POMs ([Na_7_PW^VI^_11_O_39_], [H_3_PW^VI^_12_O_40_], [H_3_PMo^VI^_12_O_40_], [Na_10_SiW^VI^_9_O_34_]), [H_3_PW^VI^_12_O_40_] displayed the highest photocurrent response intensity. In addition, BiVO_4_/[H_3_PMo^VI^_12_O_40_] demonstrates an enhanced response of 32.8% toward 50 ppm of NO_2_.^383^ In similarity with the previous discussion, herein, the electron–hole recombination was slowed down as the POM facilitates charge separation and photogenerated electron transfer to the semiconductor. Shi *et al.*^384^ made an interface modification on the grain boundary by integrating TiO_2_, and Ti^IV^ substituted POMs (K_5_[PW^VI^_11_Ti^IV^O_40_] and K_5_[PW^VI^_10_Ti^IV^_2_O_40_]). The resultant nanocomposite exhibited improved photoconductivity and elevated gas sensing properties towards acetone gas.^384^ Tian *et al.*^385^ investigated the effect of [H_3_PW^VI^_12_O_40_] doped In_2_O_3_ compound for gas sensing at room temperature toward formaldehyde. The doping of the POM successfully suppressed the recombination of photo-induced carriers in the system resulting in a 35% enhancement in photoconductivity alongside a 26% gas sensing response compared with pristine In_2_O_3_.^385^ Similarly, Wang *et al.*^386^ also incorporated [PW^VI^_12_O_40_]^3−^ with Cu_2_ZnSnS_4_ for high-performance NO_2_ gas sensors. The composite exhibited 88.83% enhanced gas sensing properties compared with pristine Cu_2_ZnSnS_4_ due to the restriction of electron–hole recombination and effective charge transfer through the POM.^386^ Furthermore, Sun *et al.*^387^ developed dye-sensitized TiO_2_–PW_12_ using a simple, economical sol–gel method followed by a screen-printing technique for faster NO_2_ gas sensing at room temperature under visible light irradiation. The heterostructure enabled faster separation and transportation of the photogenerated carriers as the POM acted as the electron acceptors. The effective increase in sensitivity (233.1–1 ppm) over a wide range of NO_2_ concentration (50 ppb–5 ppm) for POM decorated dye/TiO_2_ film occurred due to the expansion of the narrow bandgap of the POM doped dye under visible light without loss in thermal energy.^387^ An inorganic–organic hybrid film was fabricated by Kida *et al.* for selective H_2_ (50–500 ppm) and NH_3_ (10–100 ppm) sensing using yttrium-stabilized zirconia with Mo^VI^_7_O_24_^6−^/hexylamine hybrid film. Calcination of the POM alkylamine hybrid film resulted in porous MoO_3_ particles, making them an effective precursor for synthesizing nanosized metal oxide.^388^ POM-based supramolecular chemosensors were developed for the acute gas sensing of toxic gases. Wei *et al.* demonstrated a CO_2_ sensor using Na_9_DyW^VI^_10_O_36_ and block copolymer poly(ethylene oxide-*b-N*,*N*-dimethyl aminoethyl methacrylate).^389^ Likewise, Guo *et al.* developed POM-based supramolecular chemosensors for H_2_S detection (detection limit 1.25 μM) with dual signals (*via* absorption spectra and fluorescence).^390^ In the field, rapid detection of acutely corrosive and toxic gases like H_2_S at room temperature is important. Bezdek *et al.* developed enhanced chemiresistive gas sensors to detect H_2_S using highly oxidized Pt-doped POM with single-walled CNT. They have also demonstrated ppb level detection with high stability and a wide range of selectivity.^391^ Furthermore, Liu *et al.*^392^ immobilized POMs on a polyelectrolyte matrix and then used them for the sensitive detection of NO. The ability to electrocatalyze the reduction of NO resulted in a wide range of selectivity (1 nM to 10 μM).^392^

Triethylamine gas sensors developed by Cai *et al.*^393^ exhibited ultra-sensitive selectivity and stability over repeated use. One-dimensional heterostructure nanofibers of ZnO and ZnWO_4_ were synthesized *via* POM (varying the molar ratio of H_3_PW^VI^_12_O_40_) assisted electrospinning methods. The highly porous structure of the nanofibers and the synergistic effect between the ZnO and ZnWO_4_ resulted in an enhanced relative response of 108.5 for 50 ppm triethylamine. The barrier-control electron transfer at the interface was attributed to remarkable selectivity with a low detection level of 150 ppb.^393^ The recent advances led Tian *et al.*^394^ to fabricate POM–semiconductor heterojunctions *via* a one-step coaxial electrospinning technique for the effective sensing of ethanol gas. One-dimensional tandem heterojunctions SnO_2_/POM/WO_3_ significantly increased the sensing characteristics compared with the SnO_2_/WO_3_ nanofibers. The sensitivity was optimized to 100 ppm of ethanol. The construction of the interface allowed the POM to act as the electron acceptor, promoting faster carrier separation and exhibiting enhanced sensing behavior.^394^ Next, a bottom-up POM-assisted *in situ* growth of 1D nanofilament architecture was achieved by electrospinning, followed by the thermal oxidation method for the detection of acetone. A broad range of concentration, *i.e.*, 50 ppb–50 ppm, was detected with enhanced selectivity and sensitivity owing to the charge transfer to the interface of the ZnO–ZnMoO_4_ nanofilament.^395^ A unique nanostructure was developed by Ren *et al.*^396^ using Pt-draped Si-doped WO_3_ nanowires interwoven into a three-dimensional mesoporous superstructure for low-temperature ethanol gas sensing (with a detection limit of 0.5 ppm).^396^ Selective and ultrasensitive dual detection (Raman and photochromic) of ethylenediamine gas was demonstrated by Zhang *et al.* using POM/viologen hybrid crystal. It exhibits a very low detection limit of 0.1 ppb *via* Raman signal output.^397^

### 5.3 POM-based sensors in the detection of emerging health pollutants

Very recently, Wang *et al.*^398^ synthesized isostructural Anderson-type POM-based compounds and fabricated photoelectric sensors to detect inorganic ions. Three different transition metal ions (M^II^ = Co^II^, Cd^II^, Zn^II^) were incorporated for the preparation of the [M^II^_2_(H_3_bdpm)_2_TeMo^VI^_6_O_24_·6H_2_O] (H_3_bdpm = 1,1′-bis(3,5-dimethyl-1*H*-pyrazolatemethane)) compounds which contain a 2D supramolecular layer and 1D chain structures. All prepared [M^II^_2_(H_3_bdpm)_2_TeMo^VI^_6_O_24_·6H_2_O] compounds have been successfully utilized as fluorescence sensors toward Cr_2_O_7_^2−^ at different concentrations. Furthermore, the compounds with Co^II^ and Cd^II^ also exhibited electrochemical sensing behavior for detecting NO_2_^−^ (Cd-containing compound possesses a response time of 2.16 s at a detection limit of 5.11 × 10^−5^ M alongside a sensitivity of 43.10 μA mM^−1^).^398^

POM and Zn-based complexes derived from pyrazole were reported by Tian *et al.* for photocatalysis and electrochemical sensors to detect hydrogen peroxide, bromate, and nitrite by tuning pH.^399^ Likewise, Zhang *et al.* tuned the N and O coordination donors in morpholine and piperazine derivatives to derive various POM-based compounds for photocatalysis, electrochemical, and fluorescent sensor applications (towards Hg^2+^).^400^ Furthermore, researchers explored POM-modified MOFs for various sensing applications, *e.g.*, photocatalytic, electrochemical (towards the detection of inorganic ions, H_2_O_2_, Cr(vi), bromate, *etc.*).^401–405^

All literature known polyoxometalates and their applications in sensing are summarized in Table 3.

**Table 3:** Summarization of the reported POM-based sensors

POM-based composite	POM archetype	Type of sensor	Significant results	Ref.
(P_2_W^VI^_17_Fe) and palladium NPs; NPs = nanoparticles	Wells–Dawson (Fig. 2H)	Electrochemical sensor towards H_2_O_2_ and N_2_H_4_SO_4_	The H_2_O_2_ and N_2_H_4_SO_4_ exhibit sensitivity, detection limit, and linear concentration in the range of 66.7 μA mM^−1^, 1 μM (S/N = 3), 1.5 μM to 3.9 mM, and 0.2 μA mM^−1^, 1.5 μM (S/N = 3), 2 μM to 3.4 mM, respectively	371
[M^*n*+^(H_2_O)P_5_W_30_O_110_]^(15−*n*)−^	Preyssler-type	Electrochemical sensor towards H_2_O_2_	Exhibit the lowest detection limit of 0.13 mM with a high sensitivity of 4.35 μA mM^−1^ and response time of 1 s	372
K_6_P_2_Mo^VI^_18_O_62_·H_2_O with polypyrrole	Wells–Dawson (Fig. 2H)	NO_*x*_ gas sensor	Exhibits extended linearities up to 5500 ppm NO_*x*_	381
SnO_2_/HPT composite film	Keggin (Fig. 2F)	Gas sensor for the formaldehyde and methylbenzene	Higher photoconductivity compared with pristine SnO_2_	382
BiVO_4_/H_3_PW^VI^_12_O_40_	Keggin (Fig. 2F)	NO_2_ gas sensor	Enhanced response of 32.8% towards the 50 ppm of NO_2_	383
[M^II^_2_(H_3_bdpm)_2_TeMo^VI^_6_O_24_.6H_2_O]; H_3_bdpm = 1,1′-bis(3,5-dimethyl-1*H*-pyrazolatemethane)	Anderson–Evans (Fig. 2I)	Photoelectric sensors for the detection of inorganic ions	Cd-based compound possesses a response time of 2.16 s at a detection limit of 5.11 × 10^−5^ M with a sensitivity of 43.10 μA mM^−1^	398
BiVO_4_/(H_3_PW^VI^_12_O_40_ or H_3_PMo^VI^_12_O_40_ or Na_7_PW^VI^_11_O_39_ or Na_10_SiW^VI^_9_O_34_)	Keggin (Fig. 2F)	NO_2_ gas sensor	BiVO_4_/PW_12_ exhibits highest response of 32.8% towards 50 ppm of NO_2_	383
TiO_2_/[PW^VI^_11_TiO_40_]^5−^ and TiO_2_/[PW^VI^_10_Ti_2_O_40_]^7−^	Keggin (Fig. 2F)	Acetone gas sensor	Low detection concentration level of acetone is 50 and 80 ppm forTiO_2_/[PW^VI^_11_TiO_40_]^5−^ and TiO_2_/[PW^VI^_10_Ti_2_O_40_]^7−^, respectively	384
H_3_PW^VI^_12_O_40_ doped In_2_O_3_ compound	Keggin (Fig. 2F)	Gas sensor for the formaldehyde at room temperature	35% enhancement in photoconductivity alongside a 26% of gas sensing response compared with pristine In_2_O_3_	385
H_3_PW^VI^_12_O_40_ with Cu_2_ZnSnS_4_	Keggin (Fig. 2F)	NO_2_ gas sensor	Exhibits 88.83% enhanced gas sensing property compared with pristine Cu_2_ZnSnS_4_	386

### 5.4 Summary of POM-based sensors

POM-based sensors for water pollution, air pollution, and emerging health pollutants are discussed thoroughly. In aqueous sensing, Keggin, Dawson, Preyssler, and isopolymolybdate POMs exhibit strong electrocatalytic activity toward species such as hydrogen peroxide, nitrate, bromate, nitrite, and heavy metal ions, often achieving low detection limits and quick response times. For gas sensing, POM–polymer, POM–metal oxide, and POM–semiconductor heterostructures enable the sensitive and selective detection of gases, including NO_2_, NH_3_, H_2_S, formaldehyde, acetone, ethanol, and volatile amines, mainly by promoting charge separation and reducing electron–hole recombination. Lastly, emerging health-related pollutants are addressed through advanced POM-based supramolecular systems, MOFs, and hybrid complexes that offer electrochemical, photoelectrochemical, and fluorescent sensing modes. Overall, the manuscript highlights the versatility of POMs as functional building blocks for high-performance, multifunctional sensors that operate under mild and environmentally friendly conditions.

## 6 Polyoxometalate based battery and supercapacitors

POMs emerge as an exceptional electrode component for supercapacitors (SCs) or batteries due to their high proton mobility and extraordinary redox chemistry.^406–408^ POM's variable redox activities and outstanding electron/proton transport capacities apply POM-based composite materials in electrochemical fields. As a powerful electron reservoir in the multi-electron reduction process, POM enables high proton conductivity even in the composite. This interesting behavior has led to various applications of POM-based composites such as green catalysis, sensors, and electrochemical energy storage devices (batteries and SCs). However, POMs are pH-sensitive; therefore, a well-known strategy of coordination chemistry has been used to enhance the mechanical and electrochemical properties of the electrode material for better performance.^407–411^

### 6.1 POM-based battery electrodes

#### 6.1.1 POM as the electrode for lithium-ion batteries (LiBs)

Transition metal oxides are used as the cathode/anode material for LiBs as they are oxidized to their highest oxidation state when the Li has been released.^412^ The first reported POMs for LiB are focused on polyoxomolybdates.^413^ Further improvements of the electrode material have been made by modifying the structural and electronic states of POMs, altering the reversible faradaic reaction associated with them. Vanadium-based POMs are being explored as cathode materials for rechargeable batteries to achieve high energy and power density by multi-electron redox processes *via* fast transfer of Li ions. Chen *et al.*^414^ reported Li_7_[V^V^_15_O_36_(CO_3_)] as a cathode material with a specific capacity of 250 mA h g^−1^ alongside energy and power densities of 1.5 kW h L^−1^ and 55 kW L^−1^, respectively. Additionally, Li_7_[V^V^_15_O_36_(CO_3_)] exhibits a very high potential window (1.9 to 4.0 V) for reversible redox reactions. The theoretical calculation for the specific capacity for the oxometalate mentioned above at the same potential window (by considering *n* is 14, which is the next nearest integer no. of electrons) shows the specific capacity of 259 mA h g^−1^, which is in corroboration with the experimental data.^414^ Further, the vanadium-based K_7_[NiV^V^_13_O_38_] structure is explored by Ni *et al.*^415^ The maximum discharge capacity of 218.2 mA h g^−1^ was recorded at a discharge current density of 17 mA g^−1^ with 93.2% coulombic efficiency.^415^ Thus, the nano-sized polyoxovanadates can be utilized as cathode materials for LiBs for moderate capacity and rate capability.

Furthermore, POMs are combined with carbonaceous nanostructures for better cycle and rate performance. Ma *et al.*^416^ synthesized covalent functional pyrene (Py) with [H_4_SiW^VI^_12_O_40_] (SiW_12_) and attached it to the surface of SWCNTs *via* spontaneous adsorption. SWCNT/Py-SiW_11_ exhibited an initial discharge capacity of 1569.8 mA h g^−1^ at a current density of 0.5 mA cm^−2^. However, the capacity decreased to 580 mA h g^−1^ after 100 cycles at the same current density.^416^ Graphene sheets are represented by single-layer two-dimensional sp^2^-bonded carbon atoms, having a high affinity towards POMs. Wang *et al.*^417^ synthesized environmentally friendly nanomaterials by incorporating reduced graphene oxide (rGO) with Keggin type [H_4_SiW^VI^_12_O_40_] (SiW_12_) clusters. rGO/SiW_12_ exhibits a discharge capacity of 275 mA h g^−1^ with an increased potential of 4 V at a current density of 50 mA g^−1^. The nanocomposite can hold a capacity of 120 mA h g^−1^ at 1.5 V operating potential even at a high current density of 2000 mA g^−1^.^417^ Besides carbonaceous nanostructures, POMs are often synthesized with silver nanoparticles due to their chemical structure, elevated surface area, and high electrical conductivity.^418,419^

In recent years, the POM-based composite structure has been further modified by including MXenes, *e.g.*, i) POM@PANI/Mo_2_TiC_2_T_*x*_MXene/CNTs delivers lithium storage capacity of 621 mA h g^−1^ at 0.1 A g^−1^ and promising cyclic stability (445 mA h g^−1^ after 1000 periods at 1.0 A g^−1^);^390^ and ii) PMo_12_@PPy/Ti_3_C_2_T_*x*_ delivers high capacity of 764 mA h g^−1^ at 0.1 Ag^−1^ with long cycling stability of 2000 cycles at 3 A g^−1^.^420^ Additionally, the hybridization of various POMs with different supports such as porphyrins,^420^ CoS_2_/MoS_2_/functionalized rGO,^421^ and various MOFs^422–425^ results in enhanced lithium capacity and overall stability as an anode.

#### 6.1.2 POM as the electrode for sodium-ion batteries

Besides LiBs, POM-based composites are applied as cathode/anode material for Na-ion batteries. Liu *et al.*^426^ prepared a robust composite by coating Na_2_H_8_[MnV^V^_13_O_38_] (POM) clusters on the graphene nanoflakes. The discharge process of the composite demonstrates a two-phase reaction due to the presence of V(v)/V(iv) redox couple related to Na-ion insertion, and a high capacity of 202 mA h g^−1^ is recorded at 1.5 V (at the end of the discharge). Furthermore, the composite can retain 81% of its initial capacity over 100 cycles at 0.2 C with 95% coulombic efficiency.^426^ Hartung *et al.*^427^ reported that the sodium salt of decavanadate, Na_6_[V^V^_10_O_28_], acts as a high-performance cathode material for rechargeable Na-ion batteries. The potential discharge range observed from the CV graph is within the range of 0.01–3.0 V. The capacitive process associated with the Na_6_[V^V^_10_O_28_] ion is completed by the insertion of the Na ion in the voids of [V^V^_10_O_28_]^6−^ cluster.^427^

Additionally, MOFs are proven to be effective supporting materials for POMs. Using a simple impregnation strategy, Cao *et al.*^428^ demonstrated that PMo_12_/MIL-88B/GO composite delivers an excellent specific capacity of 214.2 mA h g^−1^ for 600 cycles at 2 A g^−1^. Another example is a layer-by-layer arrangement of vanadium-based POM immobilized on Co-based MOF resulted in a capacity of 413 mA h g^−1^ due to accommodating the larger Na^+^ ions efficiently.^428^

### 6.2 POM-based supercapacitor electrodes

Electrochemical capacitors or SCs, on the other hand, are promising energy storage devices that meet a significant performance gap between batteries and electrostatic capacitors. They supply high-power electric pulses over a short time scale, exhibiting a high dynamic of charge propagation with elevated charge and discharge rates.^429^ In the maximum reported SC, high capacitance and energy are achieved by incorporating a pseudocapacitive or faradaic type of active material with a double-layer capacitive component. Mostly, metal oxides and sulfides show promising results for SC electrodes as they generate a large number of charges at the electrode interface *via* multi-step reversible redox reactions.

#### 6.2.1 Composite-type hybrid electrode

**Fig. 22:**
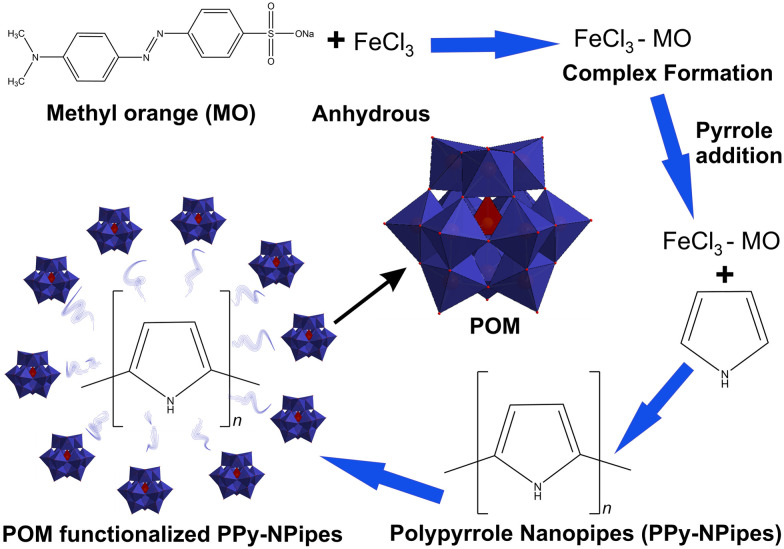
Schematic illustration of steps involved in synthesizing polypyrrole nanopipes and polyoxometalates (POMs, PMo_12_, or PW_12_) hybrid material with the simple chemical method.^408^

Early in 2005, Gómez-Romero *et al.*^407^ established the POM-based composite hybrid electrode for SC as they dispersed three different POMs, namely, [H_3_PW^VI^_12_O_40_], [H_4_SiW^VI^_12_O_40_], and [H_3_PMo^VI^_12_O_40_], in the conducting polymer PANI. The highest specific capacitance of 120 F g^−1^ with cycle stability over 1000 cycles was observed for PANI/[H_3_PMo^VI^_12_O_40_], which is higher than the other two POM ([H_3_PW^VI^_12_O_40_], [H_4_SiW^VI^_12_O_40_]) composite, due to the higher proton conductivity of the [H_3_PMo^VI^_12_O_40_] in 1 M HClO_4_ electrolyte.^407^ In the later years, the same group deposited [H_3_PMo^VI^_12_O_40_] on different conducting polymers (*e.g.*, poly(3,4-ethylenedioxythiophene) (PEDOT)) with an external oxidizing agent (H_2_O_2_) for further electrochemical improvement (Fig. 22).^408^ Later, the Freund's^430^ group used the same Keggin POM, [H_3_PMo^VI^_12_O_40_], incorporated into the porous PPy, exhibiting a specific capacitance of 210 F g^−1^ in 0.5 M H_2_SO_4_ electrolyte in three-electrode configuration.^430^ Recently, Vannathan *et al.*^431^ reported high-performance pseudocapacitors of vanadium substituted Keggin POMs and combined with a conducting polymer for enhancement of electrochemical activity.^431^

**Fig. 23:**
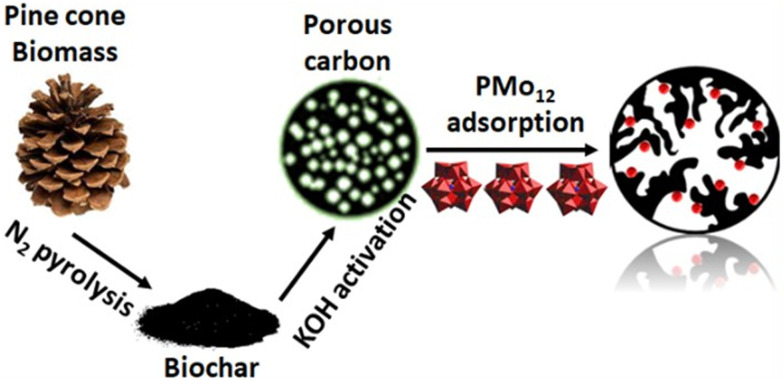
Synthesis schematic for porous pinecone biomass carbon and fabrication of pinecone – polyoxometalate hybrid material.^437^

Carbonaceous nanostructures (*e.g.*, CNT, GO/rGO, AC) come into play as the supporting elements to the POMs as they provide better mechanical and electrochemical stability.^429^ To replace the conducting polymer as a supporting element for POM, inventors need a high electrical conducting substrate like the former. CNTs exhibit higher electrical conductivity due to their hierarchical architecture among all the carbonaceous nanostructures. At first, Cuentas-Gallegos *et al.*^432^ prepared a single-wall CNT and POM composite using Cs substituted phosphomolybdate (Cs-[PMo^VI^_12_O_40_]^3−^). The composite material presented a specific capacitance of 285 F g^−1^ and an energy density of 57 W h kg^−1^.^432^ Later Skunik *et al.*^433^ further developed this concept using multi-walled CNT instead of a single wall. Phosphomolybdic acid-modified multi-walled CNT revealed a specific capacitance of 40 F g^−1^ at a discharged current of 7 mA.^433^ Furthermore, to achieve a higher surface area substrate without compromising electrical conductivity, the researchers employed AC as a supporting material because it possesses a larger surface area (up to 3000 m^2^ g^−1^) with different pore distribution (micro, meso, or macropores). Ruiz *et al.*^434^ prepared a hybrid electrode by integrating activated carbon with Keggin-type phosphomolybdate [H_3_PMo^VI^_12_O_40_] (PMo_12_). The highest specific capacitance was generated due to the faradaic component, around 183 F g^−1^ at 2 A g^−1^ current density.^434^ In 2014, the same group used molybdenum-based POMs instead of phosphotungstate [H_3_PW^VI^_12_O_40_] for an electrochemical study and observed an enhancement of the capacitance to 254 F g^−1^ in an operating potential of 1.6 V. Moreover, the composite can possess 98% capacitance over 30 000 cycles.^435^ Besides Keggin-type POMs, Mu *et al.*^436^ for the first time embedded a Dawson-type POM, (NH_4_)_6_[P_2_Mo^VI^_18_O_62_] on AC and achieved the highest capacitance of 308 F g^−1^ at 2 A g^−1^ current density due to the high proton conductivity and unique redox behavior of the faradaic component.^436^ Besides commercially available activated carbon, Lian *et al.* used biomass-derived pinecone activated carbon, in which POMs (PMo^VI^_12_O_40_^3−^) contributed to a high specific capacitance of 361 F g^−1^, showing the trend of proton-coupled electron transfer (Fig. 23).^437^ Recently, Maity *et al.*^438^ developed vanadium-substituted Keggin structures (PMo^VI^_11_VO_40_ and PMo^VI^_10_V^V^_2_O_40_) impregnated into the surface of AC. The vanadium concentration in the polyanion plays a vital role as it decides the morphology and microstructure of the nanocomposite.^438^

Graphene or its oxide derivatives (GO and rGO) are used mainly as substrate components other than CNTs and AC because of their high surface area with sizeable electrical conductivity. Additionally, the presence of oxygen-containing functional groups in GO and rGO enables many active sites for the physisorption of a faradaic component. Gómez-Romero and his team did permutation and combined possible routes to achieve high-performance SC using POM and graphene offshoots.^439,440^ In this course, they have found a new route to synthesize the hybrid PMo_12_–rGO nanoelectrode with a hydroquinone-doped hybrid gel hybrid electrolyte. The double hybridization enhances cell potential (1.6 V) and electrochemical properties by increasing the volumetric capacitance to 3.18 F cm^−3^. Similarly, for the phosphotungstate composite (rGO–PW_12_), the areal capacitance is calculated as 2.95 F cm^−3^.^439,440^

**Fig. 24:**
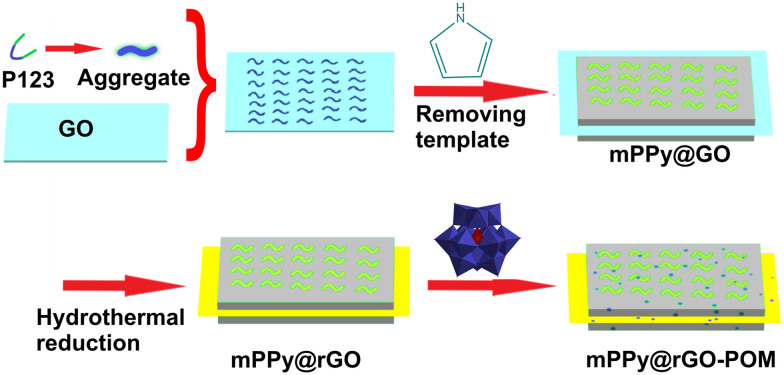
Scheme illustration of fabrication procedure of mPPy@rGO-POM nanosheets.^441^

Instead of a single supporting medium for POMs, Qin *et al.*^441^ (Fig. 24) prepared a new type of composite by anchoring PMo_12_ to PPy/rGO by layer-by-layer deposition for high-performance micro-SC in solid gel electrolyte medium (PVA/H_2_SO_4_; PVA = polyvinyl alcohol). The resultant composite exhibited high energy and power densities of 4.8 mW h cc^−1^ and 645.1 mW cc^−1^, respectively. Also, due to the presence of a solid electrolyte, it presents excellent mechanical flexibility (96% capacitance retention at a highly bending angle of 180°).^441^ Furthermore, surface modifications of graphene derivatives were made using various POM structures, demonstrating enhanced electrochemical performances.^442–445^ To achieve seamless ion transportation to the electrode/electrolyte interface Maity *et al.*^446^ designed and tailored a facile bottom-up approach in which vanadium-substituted Keggin POMs (PMo_11_VO_40_) were used to oxidize pyrrole monomer followed by the deposition on the GO surface. The resultant nanohybrid not only exhibits unique architecture but displays high-performance supercapacitive behavior.^446^ The designing and construction of polyoxometalates-based metal–organic frameworks composites further expands the search for promising high-performance electrode materials for SCs. A Dawson type^447^ the basket-shaped heteropoly blue,^448^ Keggin type,^449^ and Anderson type^450,451^ POMs hybridized in metal or covalent organic frameworks overcome the limitations of POMs, *e.g.*, high solubility in common electrolytes and results in better stability over longer cycles with improved capacitance.

#### 6.2.2 Asymmetric type hybrid electrode

Asymmetric type hybrid enhances electrochemical performances in two ways; for instance, incorporating two types of material in a single device enables different charge storage mechanisms simultaneously. Secondly, the cell voltage is tuneable (mainly can be enhanced) due to the presence of various active materials in electrodes. Chen *et al.*^452^ studied the electrochemical properties of vanadium-based iso-polyanion, sodium decavanadate ([Na_6_V^V^_10_O_28_]) in 1 M LiClO_4_ organic solution, exhibiting an excellent electrochemical behavior in a 3-electrode configuration. Furthermore, an asymmetric SC configuration was developed using activated carbon as the positive and [Na_6_V^V^_10_O_28_] as the negative electrode, exhibiting a maximum specific capacitance of 269 F g^−1^, with energy and power densities of 73 W h kg^−1^ and 312 W kg^−1^, respectively, in a 2.8 V operating potential.^452^ Hu *et al.*^453^ studied a composite type of electrode using regular PMo_12_ anchored on AC in a protic ionic liquid electrolyte. Later, the nanocomposite was assembled as an asymmetric SC device with commercially available AC. The asymmetric cell operates in an elevated potential window of 0–0.85 V, even at a high current density (10 A g^−1^).^453^

Dubal *et al.*^454^ developed a high-performance symmetric SC based on PMo^VI^_12_ and PMo^VI^_12_–rGO. They assembled an asymmetrical SC device using rGO–PMo^VI^_12_ and rGO–PW^VI^_12_ electrodes for higher energy density. The SC cell also operates at 1.6 V potential and elevated energy density of 39 Wh kg^−1^ at a power density of 658 W kg^−1^.^454^ Maity *et al.*^455^ optimized the effective loading of POM (NiV^V^_14_O_40_)^7−^ on the AC surface for the first time and employed the nanocomposite as the cathode in an asymmetric configuration with AC as the anode. The resultant device exhibited an enhanced specific energy of 90 W h kg^−1^ and specific power of 2400 W kg^−1^. Moreover, the nanocomposite-based asymmetric configuration with pristine POM as the positive electrode showed supercapattery behavior.^455^

All literature-known POM-based batteries and supercapacitors are summarized in Table 4.

**Table 4:** Summarization of the reported POM-based battery and supercapacitors

POM-based composite	POM archetype	Type of energy storage	Significant results	Ref.
Li_7_[V^V^_15_O_36_(CO_3_)]	Spherical isopolyvanadate	Li-ion battery	Specific capacity of 250 mA h g^−1^ alongside energy and power density of 1.5 kW h L^−1^ and 55 kW L^−1^, respectively	414
SWCNT/Py-SiW^VI^_11_; SWCNT = single-walled carbon nanotubes	Lacunary Keggin	Li-ion battery	Exhibits an initial discharge capacity of 1569.8 mA h g^−1^ at a current density of 0.5 mA cm^−2^	416
Na_2_H_8_[MnV^V^_13_O_38_] cluster on the graphene nanoflakes	Trimeric polyoxovanadate	Na ion battery	High capacity of 202 mA h g^−1^ is recorded at 1.5 V with 81% of its initial capacity retention over 100 cycles	426
PANI/H_3_PMo^VI^_12_O_40_; PANI = polyaniline	Keggin	Composite type SC	Highest specific capacitance of 120 F g^−1^ with cycle stability over 1000 cycles	407
([PV^V^Mo^VI^_11_O_40_]^4−^, [PV^V^_2_Mo^VI^_10_O_40_]^5−^) with AC	Keggin	Composite type SC	AC–VMo_11_ composite displayed an enhanced capacitance of 450 F g^−1^ with an improved energy density of 59.7 W h kg^−1^ alongside 99.99% capacitance retention of over 5000 cycles	438
PMo^VI^_12_ to PPy/rGO by layer-by-layer deposition; PPy = polypyrrole; rGO = reduced graphene oxide	Keggin	Composite type SC	Composite possesses high energy and power densities of 4.8 mW h cc^−1^ and 645.1 mW cc^−1^, respectively	441
[MnV^V^_14_O_40_]^6−^ on the AC and GO; AC = activated carbon; GO = graphene oxide	Lindqvist	Composite type SC	AC/MnV_14_ nanohybrid exhibits a specific capacitance of 547 F g^−1^ with specific energy and power of 76 W h kg^−1^ and 1600 W kg^−1^, respectively, at 0.8 Ag^−1^ current density. GO/MnV_14_ shows a specific capacitance of 330 F g^−1^ with specific energy and power of 30 W h kg^−1^ and 1276 W kg^−1^, respectively, at the same current density	445
PMo^VI^_12_ anchored on AC in a protic ionic liquid; AC = activated carbon	Keggin	Asymmetric SC	Asymmetric cell operates in a potential window of 0–0.85 V at 10 A g^−1^ of current density	453
rGO–PMo^VI^_12_ and rGO–PW^VI^_12_; rGO = reduced graphene oxide	Keggin	Asymmetric SC	The cell operates at 1.6 V potential and elevated energy density to 39 W h kg^−1^ with a power density of 658 W kg^−1^	454
AC//AC-K_2_H_5_[NiV^V^_14_O_40_]; AC = activated carbon	Lindqvist	Asymmetric SC	Increased the potential window up to 1.5 V and enhanced the specific energy and power values (90.1 W h kg^−1^ and 2400 W kg^−1^, respectively), with 98% coulombic efficiency	455

### 6.3 Summary of POM-based batteries and supercapacitors

The use of polyoxometalates (POMs) as advanced electrode materials for electrochemical energy storage highlights their remarkable redox activity, high proton mobility, and fast electron/proton transport. These inherent qualities make POMs appealing for use in batteries and supercapacitors, although their sensitivity to pH and solubility issues necessitate structural modifications and hybridization *via* coordination chemistry to develop mechanically durable and electrochemically stable electrodes. In batteries, especially those based on vanadium- and molybdenum-based clusters, POMs serve as active materials in lithium- and sodium-ion batteries. Their multi-electron redox processes allow for moderate to high specific capacities and a wide range of operating potentials. Hybridizing POMs with conductive supports, such as carbon nanotubes, graphene, MXenes, metal nanoparticles, MOFs, and polymer matrices, significantly improves capacity retention, rate performance, and long-term cycling stability. These approaches effectively overcome the limitations of pure POMs and facilitate efficient ion accommodation. In supercapacitors, POM-based composite and asymmetric electrodes bridge the performance gap with batteries by combining faradaic pseudocapacitance and electric double-layer storage. Key supports such as conducting polymers, carbon materials, graphene derivatives, and porous carbons enhance electrical conductivity, surface area, and mechanical strength. Advanced hybrid structures—including layer-by-layer assemblies, POM–graphene gels, MOF-supported POMs, and asymmetric devices—offer high specific capacitance, broader voltage ranges, excellent energy and power densities, and long cycle life. Overall, this manuscript presents POM-based composites as versatile, high-performance electrode platforms for future energy storage solutions.

## 7 Conclusions and outlook

It is almost impossible to overemphasize the applications of POMs in environmental remediation. By looking at the number of environmental studies mentioning POMs in the removal of various pollutants from water, soil or air, it seems that POMs are involved everywhere. This increasing number of environmental degradation studies (Fig. 3) involving POMs could be mostly explained by the versatility of the structural chemistry of POMs (Fig. 2) and the catalytic features specific to transition metals.

POMs in water column filters and/or in porous organic–inorganic composites proved to be effective in the removal of toxic heavy metals, aromatic organic pollutants, and bacteria (Fig. 4, 5 and 7). POMs in porous nanosheets are capable of the photocatalytic degradation of emergent pollutants, particularly antibiotics (Fig. 8, Tables 1 and S2), with enhanced photocatalytic performance under visible light (Fig. 9), but also dyes, plastics, industrial chemicals, and pesticides (Tables 1 and S2). Moreover, a magnetic core enclosed by polyoxometalate-based ionic liquid phases (Fig. 12) was used to remove dyes, heavy metals, microbes, and microplastics (MPs). MPs are not only one of the new emergent health pollutants but also a major one of worldwide concern, in addition to being associated with joint contamination with heavy metals.

POMs, alone and/or in combination with other compounds, such as metal–organic frameworks (MOFs), carbon nanotubes (CNTs) and mesoporous silica supports, have shown promising results in the removal of air pollutants from fossil fuels due to their selective catalytic properties for the oxidation of sulfur compounds (Fig. 13–15, Table S1). In addition, toxic gases such as hydrogen sulfide, nitrogen oxides and sulfur dioxide are efficiently removed by POMs (Fig. 16, Table 2), whereas the volatile organic compounds' reaction mechanism involves a photocatalytic oxidation catalyzed by the PW_12_/g-C_3_N_4_ hybrid material (Fig. 17).

The immobilization of POMs on different supporting surfaces facilitates their electrochemical properties for sensor application (Fig. 19, Table 3). Conversely, their variable redox activities and outstanding electron/proton transport capacities make POM-based composite materials suitable for use in electrochemical fields as an exceptional electrode component for supercapacitors and batteries (Table 4). A high-performance pseudocapacitor was obtained by replacing multiple Mo centers in [H_3_PMo^VI^_12_O_40_] with vanadium and incorporating modified a phosphomolybdate with a conducting polymer for improved electrochemical activity (Fig. 20), whereas a biomass-derived pinecone activated carbon, that includes POMs contributed to a high specific capacitance (Fig. 21). Carbon nanostructures, graphene oxide/reduced graphene oxide, and activated carbon composites come into play as supporting elements for the POMs as they provide better mechanical and electrochemical stability for broader electrochemical applications (Fig. 22 and 23). Although this review does not reveal everything, it may help to get closer to viable solutions for the effective use of the POM-based materials for the removal of the environmental pollutants. The future is bright for POM applications in environmental treatments!

## Conflicts of interest

There are no conflicts to declare.

## Abbreviations

Ac: Acetic acid
AC: Activated carbon
AOP: Advanced oxidation process
APTMS: 3-Aminopropyltrimethoxysilane
APTS: γ-Aminopropyltriethoxysilane
Asp: Aspartic acid
Bbi: 1,1′-(1,4-Butanediyl)bis(imidazole)
BE: Berberine
bimb: 1,4-Bis(1-imidazolyl)benzene
bipy: Bipyridine
BMIM or bmim: 1-Butyl-3-methylimidazolium
BPA: Bisphenol A
BPA-Br: Bromobisphenol-A
BPy: 1-Butylpyridinium or *N*-butylpyridinium
BR46: Basic red 46
BT: Benzothiophene
BTC: 1,3,5-Benzenetricarboxylate
CCNF: Carbonized cellulose nanofiber
CNTs: Carbon nanotubes
CP: Chlorphenole
4-CP: 4-Chlorphenole
CPF: Ciprofloxacin
CPBPY: *N*-(3-Carboxyphenyl)-4,4′-bipyridinium
cpt: 4-(4′-Carboxyphenyl)-1,2,4-triazolate
CSH: Cellulose propylamine-modified silica
CTS: Chitosan
CV: Crystal violet
DBP: Di-*n*-butyl phthalate
DBT: Dibenzothiophene
DESs: Deep eutectic solvents
DMDBT: 4,6-Dimethyldibenzotiophene
DODA·Br: Dimethyldioctadecylammonium bromide
DODMAC: Dimethyldioctadecylammonium chloride
ECSA: Electrochemically active surface area
EDA-CD: Per-(6-deoxy-6-iodo)-β-cyclodextrin
ELSA: Electrochemically active surface area
en: Ethylenediamine
EPs: Emergent pollutants
EtOH: Ethanol
etpy: 4-Ethylpyridine
EY: Eosin Y
g-BN: Graphene-like hexagonal boron nitride
GA: Graphene aerogel
GO: Graphene oxide
Gr: Graphene
HOMO: Highest occupied molecular orbital
HPW or PW_12_: [H_3_PW^VI^_12_O_40_·6H_2_O]
H_2_pyttz-I: 3-(Pyrid-2-yl)-5-(1*H*-1,2,4-triazol-3-yl)-1,2,4-triazolyl
H_2_pyttz-II: 3-(Pyrid-4-yl)-5-(1*H*-1,2,4-triazol-3-yl)-1,2,4-triazolyl
H_3_bdpm: 1,1′-Bis(3,5-dimethyl-1*H*-pyrazolate)methane
IBA: Isobutyraldehyde
IBAc: Isobutyric acid
IBP: Ibuprofen
IL: Ionic liquid
imi: Imidazole
iPAF-1: Porous aromatic framework
LiBs: Lithium-ion batteries
LDH: Layered double hydroxide
LMCT: Ligand to metal charge transfer
LPMS: Large-pore mesoporous silica
LRSR: Liquid-redox sulfur recovery
LUMO: Lowest unoccupied molecular orbital
MB: Methylene blue
MBT: 2-Mercaptobenzothiazole
MCM-41: Conventional molecular sieve MCM-41
MeCN: Acetonitrile
MeOH: Methanol
mepy: 4-Methylpyridine
MO: Methyl orange
MOFs: Metal–organic frameworks
MOG: Metal–organic gel
MPs: Microplastics
MR: Methyl red
M-TCS: Methyl triclosan
NAD: 2-(1-Naphthyl)acetamide
NBZ: Nitrobenzene
NFZ: Nitrofurazone
NPs: (Metal) nanoparticles
ODS: Oxidative desulfurization
PANI: Polyaniline
PBV: Patent blue V
pca: Pyridine-2-carboxylic acid
PDDA: Poly(diallyldimethylammonium chloride)
PEI: Polyetherimide
phen: 1,10-Phenanthroline
PIL: Protic ionic liquid
PMIn: Polyionene
PMOE: (Ethylene-bridged) periodic mesoporous organosilica
PMs: Particulate matters
POM: Polyoxometalate
POMCP: POM-based coordination polymer
POM-IL: Polyoxometalate-based ionic liquid
POMos: Polyoxomolybdates
POM-SILP: Polyoxometalate-supported ionic liquid phase
POT: Polyoxotungstate
PPI: Proton pump inhibitor
PPy: Polypyrrole
PS: Ponceau S
PTMS: 3-Aminopropyl trimethoxysilane
PVA: Polyvinyl alcohol
PVDF: Polyvinylidene fluoride
py: Pyrene
PyPS: 3-(Pyridine-1-ium-1-yl)propane-1-sulfonate
PZC: Point-of-zero charge
RB: Rose bengal
RB5: Reactive black 5
RhB: Rhodamine B
RH: Rice husk
rGO: Reduced graphene oxide
SAB: Sodium-activated bentonite
SBA-15: Aminosilylated silica
SC: Supercapacitor
SCR: Selective catalytic reduction
SDV: Sodium decavanadate
SMT: Sulfamethazine
SPD: Sulfapyridine
SPME: Solid-phase microextraction
SSA: 5-Sulfosalicylic acid
SSZ: Sulfasalazine
SWCNTs: Single-walled carbon nanotubes
TB: Toluidine blue
TBA: Tetra-*n*-butylammonium ion
TBBA: Tetrabromobisphenol-A
TC: Tetracycline
TCS: Triclosan
TCY: Tetracycline
TMA: *N*-Trimethoxysilypropyl-*N*,*N*,*N*-trimethylammonium
TMR4A: Resorcin[4]arene-based ligand
TOA: Tetraoctylammonium
TPD-MS: Temperature-programmed desorption-mass spectroscopy
VOCs: Volatile organic compounds
4,6-DMDBT: 4,6-Dimethyl dibenzothiophene
[mim(CH_2_)_3_COO]: 1-Carboxypropyl-3-methyl imidazole
[C_4_mim]^+^: 1-Butyl-3-methylimidazolium ion
β-EDA-CD: Per-6-deoxy-6-ethylenediamine-β-cyclodextrin

## Supplementary Material

EN-013-D5EN00964B-s001

## Data Availability

No primary research results, software or code have been included and no new data were generated or analyzed as part of this review. Supplementary information (SI): the SI includes summary tables of recently published studies on POM-based water treatment technologies (section 2) and POM-based catalysts for the removal of refractory sulfur compounds from fossil fuels (section 4.1). See DOI: https://doi.org/10.1039/d5en00964b.
