# Supplementary material for: Polyoxometalates in environmental remediation and energy storage
Source: Environ Sci Nano. 2026 Feb 2;13(3):1295–346. doi: 10.1039/d5en00964b (PMC12962238; doi:10.1039/d5en00964b)
Supplement: EN-013-D5EN00964B-s001 [file EN-013-D5EN00964B-s001.pdf]

Supplementary Information (SI) for Environmental Science: Nano.
This journal is © The Royal Society of Chemistry 2026

Supplementary Materials for

Polyoxometalates in Environmental Remediation and Energy Storage

Ingrid Gregorovic, Nahid Lotfian, Ruhollah Khajavian, Sukanya Maity, Masoud Mirzaei*, Sib Sankar Mal*, Manuel Aureliano* and Annette Rompel*

*Corresponding authors. E-mail: mirzaeesh@um.ac.ir, malss@nitk.edu.in, maalves@ualg.pt and annette.rompel@univie.ac.at

This PDF file includes:

Tables S1 to S2

1. POM-based catalysts in removal of refractory sulfur compounds from fossil fuels

Table S1 The summary of literature known POM-based catalysts and their efficiency in removal of refractory sulfur compounds from fossil fuels.

Formula	POM archetype	Conditions	Efficiency	Number of cycles	Ref.
$[H_3PW^{VI}_{12}O_{40}\cdot 20H_2O]$; $[Na_3PW^{VI}_{12}O_{40}\cdot 14H_2O]$; $[H_3PMo^{VI}_{12}O_{40}\cdot 15H_2O]$; $[Na_3PMo^{VI}_{12}O_{40}\cdot 20H_2O]$; $[H_4SiW^{VI}_{12}O_{40}\cdot 25H_2O]$; $[H_4SiMo^{VI}_{12}O_{40}\cdot xH_2O]$, x = not specified by authors (nsp)	Keggin	T = 50-70°C; t = 90 min; solvent – toluene; 30 % H_2O_2 as oxidant; n(catalyst) = 0.063 mmol; n(DBT) = 5.43 mmol; DBT = dibenzothiophene	DBT removal efficiency of 100 % within 90 min (k = 0.064 min^{-1})	nsp	[1]
POM/PEG/SSA; POM = $Na_3H_6Cr^{III}Mo^{VI}_6O_{24}$, PEG = polyethylene glycol, SSA = 5-sulfosalicylic acid	Anderson-Evans	T = 60 °C; t = 2 h; $p(O_2)$ = 1 atm, 60 mL/min O_2 flow rate; V_{oil}/V_{DES} = 5; O_2 as oxidant; model diesel ([S] = 500 ppm in decalin); V(model diesel) = 20 mL; m(POM) = 0.02 g; V(DES)= 4 mL; DES = deep eutectic solvent; DBT = dibenzothiophene	DBT removal efficiency of 100 % within 120 min (k = 0.028 min^{-1})	5	[2]
$H_8PV^V_5Mo^{VI}_7O_{40}$ (HPA-5)	Keggin	T = 140 °C; t = 6 h; p = 20 bar; n(HPA-5) = 2.50 mmol; V_{H2O}/V_{oil} = 10; O_2 – oxidant; model oil (3.35 g of BT in 100 mL isooctane; [S] = 11483 ppm); BT = benzothiophene	BT removal efficiency of 99 % within 6 h	at least 3	[3]
CNTs@PDDA@$Mo_{16}V_2$; $Mo_{16}V_2$ = $H_8P_2Mo^{VI}_{16}V^V_2O_{62}\cdot mH_2O$, m = not specified by authors (nsp), CNTs = carbon nanotubes, PDDA = poly(diallyldimethylammonium chloride)	Wells-Dawson	T = 70 °C; t= 3 h; O_2 flow 1.5 L/min; O_2 as oxidant; [catalyst] = 1.0 g/L; model fuel (2.87 g DBT in 250 mL n-octane; [S] = 2000 ppm); DBT = dibenzothiophene	DBT removal efficiency of 99.4 % within 3 h	8	[4]
$[C_4VIM]PMoV_2$; $PMoV_2$ = $H_5PMo^{VI}_{10}V^V_2O_{40}$, $[C_4VIM]$ = 1-butyl-3-vinylimidazolium cation	Keggin	T = 120 °C; t = 5 h; V(air) = 100 mL/min; m(catalyst) = 0.05 g; model oil ([S] = 200 ppm; DBT, 4-MDBT or 4,6-DMDBT in dodecane); V(model oil) = 20 mL; DBT = dibenzothiophene; 4-MDBT = 4-methyldibenzothiophene; 4,6-DMDBT = 4,6-dimethyldibenzothiophene	desulfurization efficiency of 98.9 % within 5 h	7	[5]
Co-Pc/PMoV; PMoV = $[(NH_4)_5H_6PV^V_8Mo^{VI}_4O_{40}\cdot 27H_2O]$, Co-Pc = cobalt phthalocyanine	Keggin	T = 100 °C; t = 5 h; p = 1 atm; m(catalyst) = 12 mg; O_2 – oxidant; model oil ([DBT] = 500 ppm in 6 mL decalin); DBT = dibenzothiophene	DBT removal efficiency of 97.6 % within 5 h (k = 24.21 h^{-1}) and for inorganic sulfur S^{2-} (k = 29.12 h^{-1})	at least 9	[6]
$Tb(PW_{11})_2$@MIL-101; $Tb(PW_{11})_2$ = $[Tb(PW^{VI}_{11}O_{39})_2]^{11-}$, MIL-101 = metal-organic framework	lacunary Keggin	T = 50 °C; t = 2-5 h; solvent – MeCN; DBT = dibenzothiophene	DBT removal efficiency of 95 % after 2 h, 100 % efficiency after 5 h	3	[7]
0,1-C_{16}SiW–TiO_2; SiW = $[Si^{IV}W^{VI}_{12}O_{40}]^{4-}$	Keggin	T = 50 °C; t = 60 min; m(catalyst) = 10 mg; $n(H_2O_2)$ = 2 mol; n(O/S) = 2; model oil (DBT, 3-MBT, 4-MDBT in n-octane, [S] = 500 ppm); V(model oil) = 5 mL; DBT = dibenzothiophene; 3-MBT = 3-methylbenzothiophene; 4-MDBT = 4-methyldibenzothiophene	DBT removal efficiency of 95.3 % within 60 min	8	[8]
PIL/$H_2W^{VI}_{12}O_{42}^{10-}$; PIL = Polymeric Ionic Liquid	paratungstate	T = 30 °C; t = 90 min; m(catalyst) = 25 mg; m(model oil) = 10 g; model oil (BT and DBT in n-dodecane, $[S]_0$ = 1000 ppm); real diesel ([S] = 559.7 ppm); H_2O_2 as oxidant; $n(H_2O_2/S)$ = 4; DBT = dibenzothiophene; BT = benzothiophene	DBT removal efficiency of 92.1 % and BT removal efficiency of 58.3 % within 90 min	8	[9]
		T = 60 °C; t = 3-5 h; solvent – hexane; V(hexane) = 5 mL; extraction solvent – DMF or MeCN; V(DMF or MeCN) = 5 mL or 50 mL;	DBT removal efficiency of 99 %, 4,6-DMDBT removal efficiency of 80 %		

		$[AcOH]/[H_2O_2]$ = 1; [substrate] = 0.01 M; [catalyst] = 1.25 x 10^{-4} M; substrate/catalyst = 80-100; DBT = dibenzothiophene; 4,6-DMDBT = 4,6-dimethyldibenzothiophene	(*k*(BT) = 0.0103 min^{-1}; *k*(DBT) = 0.0171 min^{-1} and *k*(4,6-DMDBT) = 0.0152 min^{-1})		
$Na_3Fe^{III}(OH)_6Mo^{VI}_6O_{18}$/PEG2000/BSA; PEG2000 = Polyethylene Glycol 2000, BSA = Bovine Serum Albumin	Anderson-Evans	T = 60 °C; t = 60 min; p= 1 atm; 2000 PEG/2.5 BSA (n(PEG2000)/n(BSA) = 2.5); O_2 as oxidant; m(catalyst) =10 mg; m(DESs) = 4 g; model oil ($[S]_0$ = 500 ppm); V(model oil) = 20 mL; DESs= deep eutectic solvents; DBT = dibenzothiophene; 4,6-DMDBT = 4,6-dimethyldibenzothiophene	DBT and 4,6-DMDBT removal efficiency of 99 % in 180 min, BT 95 % in 240 min	5	[11]
$[Bmim]_3[PW^{VI}_{12}O_{40}]$; $[Bmim]^+$ = 1-butyl-3-methylimidazolium cation	Keggin	T = 80 °C; p =1 atm; H_2O_2 – oxidant; $[Bmim]BF_4$ – solvent; m(catalyst) = 0.5 g; V(solvent) = 5 ml; m(petcoke) = 0,5 g; 5 mL of 30 % H_2O_2; t = 5 h + drying for 24 h at 100 °C; petcoke pretreated by tetrabutylammonium chloride	sulfur removal efficiency of 36.10 % within 5 h	nsp	[12]
$K_x[PMo^{VI}_{12}O_{40}]$; x = 1, 2, 3, 4	Keggin	T = 60 °C; t = 60 min; V(model oil) = 15 mL; $V(CH_3OH)$ = 15 mL; m(catalyst) = 0.2 g; H_2O_2 – oxidant; $n(H_2O_2)$/n(DBT) = 4; V(model oil)/$V(CH_3OH)$ =1.5:1; DBT = dibenzothiophene	DBT removal efficiency of 99 % within 60 min for x = 4 (*k* = 0.076 min^{-1})	5	[13]
$P[C_2V]Mo^{VI}V^V$/AC; MoV = $H_8P_2Mo^{VI}_{16}V^V_2O_{62}$; AC = activated carbon	Wells-Dawson	T = 70 °C; t = 180 min; O_2 – oxidant; model oil (DBT, 4-MDBT or 4,6-DMDBT in n-octane; [S] = 2000 ppm); V(model oil) = 50 mL; O_2 flow rate 1.5 L/min; n(POM) = 15 µmol; DBT = dibenzothiophene; 4,6-DMDBT = 4,6-dimethyldibenzothiophene; 4-MDBT = 4-methyldibenzothiophene	DBT removal efficiency of 99.2 % within 180 min	8	[14]
n-[Pmim]PMo/HAP; *n* = 10, 25; PMo = $H_3PMo^{VI}_{12}O_{40}$, *n*-$[Pmim]^+$ = 1-propyl-3-methylimidazolium cation, HAP = hydroxyapatite	Keggin	T = 40 °C; t = 60 min; n(O/S) = 6; m(catalyst) = 0.1 g; model oil (DBT in n-octane; [S] = 500 ppm); V(model oil) = 5 mL; V(IL) = 1 mL; H_2O_2 – oxidant; DBT = dibenzothiophene; IL = ionic liquid	DBT removal efficiency of 97.2 % in 60 min	6	[15]
POM–PAF-1; POM = $[Mo^{VI}_8O_{26}]^{4-}$, PAF-1 = Porous Aromatic Framework-1	octamolybdate	T = 30 °C; t = 30 min; H_2O_2 as oxidant; $V(H_2O_2)$ = 50 µL; m(catalyst) = 40 mg; 75 µL of glacial acetic acid; V(MeCN) = 1.0 mL; $n(H_2O_2)$/n(DBT) = 6; model oil (TP, BT, DBT or 4,6-DMDBT in n-octane; [S] = 500 ppm); V(model oil) = 5 mL; TP = thiophene; BT = benzothiophene; DBT = dibenzothiophene; 4,6-DMDBT = 4,6-dimethyldibenzothiophene	DBT removal efficiency of 98.5 % within 30 min (*k* = 0.125 min^{-1})	5 (2 % activity loss)	[16]
PW/UiO-66(Zr); PW = $H_3PW^{VI}_{12}O_{40}$, UiO-66(Zr) = zirconium-based metal-organic framework	Keggin	T = rt; t = 25 min; m(catalyst) = 50 mg, m(model fuel) = 10 g; m(MeCN) = 10 g; O/S = 6; model fuel (BT, DBT or 4,6-DMDBT in n-octane; [S] =1000, 1000 or 500 ppmw); BT = benzothiophene; DBT = dibenzothiophene; 4,6-DMDBT = 4,6-dimethyldibenzothiophene	DBT removal efficiency of 98.2 % within 25 min (TOF = 293 h^{-1})	at least 4	[17]
$PW_{11}Zn$@aptesPMOE; $PW_{11}Zn$ = $[PW^{VI}_{11}Zn^{II}(H_2O)O_{39}]^{5-}$, aptesPMOE = (3-aminopropyl)triethoxysilane (APTES) grafted onto poly(methyl oxazoline) (PMOE)	lacunary Keggin	T = 70 °C; H_2O_2 – oxidant; n(catalyst) = 3 µmol; MeCN – extraction solvent; in model oil: model oil/MeCN = 1:1; $n(H_2O_2/S)$ = 4 (solvent free) and (with MeCN) = 8; t = 1 h in real diesel: in MeCN/diesel = 1:1, t = 120 min; $n(H_2O_2/S)$ = 8	complete desulfurization after 4 h for a biphasic system, 1.5 h for solvent-free system	10	[18]
CNC@PIL@POM; POM = $[Co(OH)_6Mo^{VI}_6O_{18}]^{3-}$, CNC = cellulose nanocrystals, PIL = polymeric ionic liquid	Anderson-Evans	T = 100 °C; t = 3 h; O_2 from air as oxidant; m(catalyst) = 20 mg; model diesel (BT, DBT or 4,6-DMDBT in decahydronaphthalene; [S] = 500 ppm); V(diesel) = 20 mL; BT = benzothiophene; DBT = dibenzothiophene; 4,6-DMDBT = 4,6-dimethyldibenzothiophene	Complete desulfurization within 3 h (*k* = 1.277 h^{-1})	at least 5	[19]
		T = 60 °C; t = 120 min; m(catalyst) = 50 mg; model diesel (DBT in n-hexane; [S] = 500			

		ppmw); V(model diesel) = 12.5 mL; V(TBHP) = 0.9 mL;<br>DBT = dibenzothiophene; TBHP = tert-butyl hydroperoxide; ppmw = parts per million by weight			
TBA-$PW_{11}NiO_{39}$@PANI;<br>TBA-$PW_{11}NiO_{39}$ = $((n\text{-}C_4H_9)_4N)_4H[PW^{VI}_{11}Ni^{II}O_{39}]$,<br>PANI = polyaniline	lacunary Keggin	T = 35 °C; t = 60 min; V(H_2O_2/HOAc) = 6 mL as oxidant v/v 2:1; m(catalyst) = 0.1 g; model oil (TH, BT and DBT in n-heptane; [S] = 500 ppm); V(model oil) = 50 mL; MeCN – extraction solvent; V(MeCN) = 10 mL;<br>TH = tetrahydrothiophene; BT = benzothiophene; DBT = dibenzothiophene; 4,6-DMDBT = 4,6-dimethyldibenzothiophene	DBT removal efficiency of 97 %, BT and TH removal efficiency of 96 % within 60 min<br>(*k*(DBT) = 0.936 min^{-1}, *k*(BT) = 0.9477 min^{-1}, *k*(TH) = 0.91 min^{-1})	5	[21]
$PMo_{11}Cd$@$MnFe_2O_4$;<br>$PMo_{11}Cd$ = $PMo^{VI}_{11}Cd^{II}O_{39}$	lacunary Keggin	T = 35 °C; t = 1 h; V(H_2O_2/HOAc) = 3 mL oxidant (v/v 2:1); m(catalyst) = 0.1 g; model fuel (Th, BT, DBT in n-heptane; [S] = 500 ppm); V(model fuel) = 50 mL;<br>Th = thiophene; BT = benzothiophene; DBT = dibenzothiophene	DBT removal efficiency of 98 % and BT removal efficiency of 97 % within 1h<br>(*k*(BT) = 0.091 min^{-1}, *k*(DBT) = 0.111 min^{-1})	5 (3 % of activity loss)	[22]
$(NR_4)_3[X^{III}Mo^{VI}_6O_{24}H_6]$;<br>X = Cr, Fe, Co; R = H or alkyl	Anderson-Evans	T = 120 °C; t = 1 h; O_2 as oxidant; air flow rate 6 L/h; n(S)/n(catalyst) = 50:1; model fuel (DBT in decalin, [S] = 500 ppm); V(model fuel) = 30 mL;<br>DBT = dibenzothiophene	Complete removal of DBT within 1 h (CoMo-POM)	2	[23]
$La^{III}W^{VI}_{10}O_{36}$@MIL-101(Cr);<br>$LaW_{10}O_{36}$ = $Na_7[H_2LaW_{10}O_{36}]$,<br>MIL-101(Cr) = chromium (III)-based metal-organic framework	Weakley	T = 60 °C; t = 120 min; H_2O_2 – oxidant; n-octane/MeCN biphasic solvent system; m(catalyst) = 40 mg; n(O/S) = 6; V(model gasoline) = 5 mL; V(MeCN) = 5 mL;<br>DBT = dibenzothiophene	99.1 % DBT conversion in 180 min	at least 7	[24]
PMo_{12}@UiO-67;<br>PMo_{12} = $H_3PMo^{VI}_{12}O_{40}$,<br>UiO-67 = zirconium-based metal-organic framework	Keggin	T = 50 °C; t = 30 min; H_2O_2 – oxidant; n(H_2O_2/S) = 3, m(POM@MOF) = 0.015 g; V(model fuel) = 0.5 mL; V(MeCN) = 0.5 mL; model fuel (Th, BT, DBT, 4-MDBT and 4,6-DMDBT in n-octane; [S] = 500 ppm);<br>MOF = metal-organic framework; Th = thiophene; BT = benzothiophene; DBT = dibenzothiophene; 4-MDBT = 4-methlydibenzothiophene; 4,6-DMDBT = 4,6-dimethyldibenzothiophene	95.5 % DBT conversion in 30 min	6	[25]
$Fe_2W_{18}Fe_4$@$FeTiO_3$;<br>$Fe_2W_{18}Fe_4$ = $Na_9K[(Fe^{III}W^{VI}_9O_{34})_2Fe^{III}_4(H_2O)_2]\cdot 32H_2O$	Sandwich-type	T = 35 °C; t = 60 min; H_2O_2/HOAc – oxidant (v:v 2/1, 3 mL); MeCN – extraction solvent; V(solvent) = 10 mL; m(catalyst) = 0.1 g; model fuel (Th, BT and DBT in n-heptane; [S] = 500 ppmw); V(model fuel) = 50 mL;<br>Th = thiophene; BT = benzothiophene; DBT = dibenzothiophene; ppmw = parts per million by weight	DBT removal efficiency of 99 %, BT removal of 98 % and TH removal of 96 % within 60 min<br>(*k*(Th) = 0.046 min^{-1}, *k*(BT) = 0.050min^{-1}, *k*(DBT) = 0.055 min^{-1})	5	[26]
ZIF-8@{Mo_{132}};<br>ZIF-8 = zeolitic imidazolate framework	Keplerate (nanoball)	T = 80 °C; t = 12 h; V(THBP) = 4 µL as oxidant; n(O/S) = 1; m(catalyst) = 150 mg; model fuel (500 mg DBT in 1 L of toluene); V(model fuel) = 15 mL;<br>DBT = dibenzothiophene; TBHP = tert-butyl hydroperoxide	DBT removal efficiency of 92 % within 12 h<br>(*k* = 0.0016 h^{-1})	5	[27]
$H_{11}P_2W^{VI}_{13}V^{V}_5O_{52}$/TMA-Si;<br>TMA-Si = tetramethylammonium-functionalized silica	Wells-Dawson	T = 70 °C; t = 30 min; p = 1 atm; H_2O_2 – oxidant; [catalyst] = 7.5 g/L; V(solvent)/V(model oil) = 1:6; MeCN – extraction solvent; model oil ([S] = 500 ppmw in 2,2,4-trimethyl pentane and 20 v% toluene);<br>ppmw = parts per million by weight	95 % efficiency for model oil and 83 % for real diesel in 30 min	at least 5	[28]
PW_{12}@TiO_2;<br>PW_{12} = $H_3PW^{VI}_{12}O_{40}$	Keggin	T = 60 °C; t = 60 min; H_2O_2/HOAc – oxidant; V(MeCN) = 6 mL; model oil ([S] = 500 ppm); V(model oil) = 5 mL; n(O/S) = 6; m(catalyst) = 0.09 mol;<br>DBT = dibenzothiophene	DBT removal efficiency of 99.9 % within 60 min	at least 7	[29]
(n-$C_4H_9)_4N]_7H_5[Si^{IV}_2W^{VI}_{18}Cd^{II}_4O_{68}]$	Sandwich	T = 60 °C; t = 2 h; m(catalyst) = 0.1 g; V(MeCN) = 10 mL; V(H_2O_2/HOAc, v/v 1:1) = 6 mL as oxidant; MeCN – extraction solvent; model fuel (thiophenic compounds in n-	DBT removal efficiency of 98 % within 2 h for model fuel	at least 5	[30]

		heptane; [S] = 500 ppmw); V(model fuel) = 50 mL;<br>DBT = dibenzothiophene; ppmw = parts per million by weight			
$\{Mo_{132}\}$/GO;<br>GO = graphene oxide	Keplerate (nanoball)	T = 60 °C; t = 150 min; H_2O_2 – oxidant; MeCN – extraction solvent; model oil (DBT in n-dodecane, [S] = 500 ppm); $n(H_2O_2/DBT) = 6$; [catalyst] = 10 g/L;<br>DBT = dibenzothiophene	DBT removal efficiency of 99 % for $\{Mo_{132}\}$/GO and 96 % for $\{Mo_{132}\}$ within 150 min<br>($\{Mo_{132}\}$: $k = 0.0196$ h^{-1}, $\{Mo_{132}\}$/GO: $k = 0.0311$ h^{-1})	10	[31]
$(NH_4)_5H_6PV^{V}_8Mo^{VI}_4O_{40}$	Keggin	T = 100 °C; t = 6 h; m(catalyst) = 20 mg; O_2 – oxidant; p = 1 atm; model oil (DBT in decalin; [S] = 500 ppm); V(model oil) = 6 mL;<br>DBT = dibenzothiophene; Th = thiophene	100 % conversion of DBT and 97 % of Th within 6 h	10	[32]
PhPyBs-PW;<br>PW = $H_3PW^{VI}_{12}O_{40}$,<br>PhPyBs = ionic liquids containing 4-phenyl-pyridine (PhPy) and 1,4-butane sultone (Bs)	Keggin	T = rt or 70 °C; t = 10 min; m(catalyst) = 40 mg; H_2O_2 30 % aq, n = 1 mmol; H_2O_2/S = 1:1; 4 mL of H_2O or H_2O:EtOH (5 mL, v/v = 7:3); model fuel (DBT in 4 mL n-hexane; [S] = 100, 700 and 1000 ppm);<br>DBT = dibenzothiophene	sulfur removal efficiency of 90 % within 10 min in model oil	5	[33]
$H_3PW^{VI}_6Mo^{VI}_6O_{40}$	Keggin	T = 60 °C; t = 90 min; solvent – MeCN; V(MeCN) = 60 mL; model fuel (sulfur compound in 60 mL octane; sulfur content of 0.050 mass %)	90.26 % thiophene removal efficiency within 90 min	at least 2	[34]
$[PyPS]_3Co^{III}(OH)_6Mo^{VI}_6O_{18}$/PEG2000/BSA;<br>[PyPS] = pyridylphenylsulfonate,<br>PEG2000 = polyethylene glycol 2000,<br>BSA = bovine serum albumin	Anderson-Evans	T = 60 °C; p = 1 atm; O_2 as oxidant; m(DES) = 4 g; n(HBA):n(HBD) = 1:2; V(model oil) = 20 mL; m(POM) = 20 mg; O_2 flow 60 mL/min; t = 4 h for model oil; t = 8 h for commercial diesel; T = 80 °C for commercial diesel;<br>HBA = hydrogen bond acceptor; HBD = hydrogen bond donor; DBT = dibenzothiophene	98 % DBT removal efficiency within 3 h	5	[35]
$[(NH_4)_5(CTA)_6PMo^{VI}_4V^{V}_8O_{40}]$;<br>CTA = cetyltrimethylammonium	Keggin	T = 80-100 °C; t = 4-8 h; p = 1 atm; O_2 – oxidant; model oil ([DBT] = 500 ppm, V(decalin) = 6 mL); n(catalyst) = 0.05 mmol;<br>DBT = dibenzothiophene	DBT removal efficiency of 100 % within 8 h at 100 °C	10	[36]
$[C_2(MIM)_2]PW^{VI}_{12}O_{40}$;<br>$[C_2(MIM)_2]$ = 1-ethyl-3-methylimidazolium	Keggin	T = 50 °C; t = 60 min; n(catalyst)/n(S) = 0.025; $n(H_2O_2)/n(S) = 6$; H_2O_2 – oxidant; V(model oil) = 5 mL (DBT in n-octane); V(MeCN) = 0.5 mL;<br>DBT = dibenzothiophene	DBT removal efficiency of 98.4 % within 60 min	at least 7	[37]
$([n\text{-}C_4N_9]_4N)_4H[PW^{VI}_{11}FeO_{39}]$/NiO	Keggin	T = 35 °C; t = 60 min; model oil ([S] = 500 ppm in n-heptane); V(model oil) = 50 mL; $V(H_2O_2/AcOH)$ = 3 mL (v/v = 1:2); extraction solvent – MeCN; m(catalyst) = 0.1 g;<br>DBT = dibenzothiophene	97 % of total sulfur (wt%) removed in real gasoline within 60 min<br>(DBT rate constant $k = 0.049$ min^{-1})	at least 5	[38]
Al_2O_3-$P_2W^{VI}_{15}$-C_n;<br>P_2W_{15} = $Na_{12}[\alpha\text{-}P_2W^{VI}_{15}O_{56}]\cdot 24H_2O$,<br>$C_n$, where n = 8, 12 or 18	Wells-Dawson	T = 60 °C; t = 9 min; H_2O_2 – oxidant; model oil (DBT, BT and 4,6-DMDBT in n-octane, [S] = 1000 ppm); V(model oil) = 5 mL; m(catalyst) = 116.0 mg; $V(H_2O_2)$ = 48.0 µL; real diesel sample ([S] = 425 ppm); $n(H_2O_2)/n(S) = 3:1$;<br>BT = benzothiophene; DBT = dibenzothiophene; 4,6-DMDBT = 4,6-dimethyldibenzothiophene	100 % of sulfur removal within 9 min at 60 °C	at least 10	[39]
$PW^{VI}_{12}O_{40}$@$MnFe_2O_{40}$	Keggin	T = 35 °C; t = 60 min; model fuel (500 ppm of Th, BT and DBT in n-heptane); V(model fuel) = 50 mL; V(oxidant) = 3 mL; oxidant system: H_2O_2/acetic acid v/v = 2:1; p = 1 atm; m(catalyst) = 0.1 g;<br>Th = thiophene; BT = benzothiophene; DBT = dibenzothiophene	DBT removal efficiency of 98 % in model oil and 96 % in real fuel within 60 min<br>(rate constant for DBT, $k = 0.053$ min^{-1})	5	[40]
$K_{10}[\alpha\text{-}P_2W^{VI}_{17}O_{61}]\cdot 20H_2O$/3D GO;<br>3D GO = three-dimensional graphene oxide	Wells-Dawson	for model oil: T = 60 °C; t = 30-120 min; model oil (sulfur-containing compounds: DBT, BT and 4,6-DMDBT in n-octane, [S] = 500 ppm); V(model oil) = 20 mL; m(catalyst) = 0.08 g; $V(H_2O_2$, 30 % aq) = 100 µL; H_2O_2 – oxidant; V(MeCN) = 20 mL;<br>for THT: m(catalyst) = 0.02 g; V(MeCN) = 5 mL; $V(H_2O_2$, 30% aq) = 100 µL; H_2O_2 – oxidant; T = 25 °C;	complete removal of DBT and THT within 120 min and 30 min, respectively	5	[41]

		BT = benzothiophene; DBT = dibenzothiophene; 4,6-DMDBT = 4,6-dimethyldibenzothiophene; THT = tetrahydrothiophene			
P[Vim]POM/GO; P[Vim] = 1-ethyl-3-methylimidazolium cation, POM = $H_8P_2Mo^{VI}_{16}V^{V}_2O_{62}\cdot 14H_2O$, GO = graphene oxide	Wells-Dawson	T = 60 °C; t = 60-120 min; m(catalyst) = 0.13 g; $n(H_2O_2)/n(S)$ = 9; model fuel ([S] = 2000 ppm in octane); V(model fuel) = 10 mL; extraction solvent – DMF; V(DMF) = 10 mL; DBT = dibenzothiophene; 4,6-DMDBT = 4,6-dimethyldibenzothiophene; 4-MDBT = 4-methyldibenzothiophene; BT = benzothiophene	DBT, 4,6-DMDBT and 4-MDBT removal efficiency of 100 % within 60 min; Th and BT removal efficiency of 91.8 % and 96 % within 120 min, respectively; *k*(DBT) = 0.1071 min^{-1}	7	[42]
$[Cu_{12}(BTC)_8(H_2O)_{12}][H_5PMo^{VI}_{10}V^{V}_2O_{40}]@(H_2O)_{49}$ (**1**); $[Cu_{12}(BTC)_8(H_2O)_{12}][H_4PMo^{VI}_{11}V^{V}O_4]@(H_2O)_{30}$ (**2**); BTC = benzene-1,3,5-tricarboxylic acid or trimesic acid	Keggin	T = 65 °C; t = 5 h; m(catalyst) = 0.03 g; n(substrate) = 0.5 mmol; n(O)/n(S) = 6; $V(CH_2Cl_2)$ = 5 mL; DBT = dibenzothiophene	DBT removal efficiency of 89.6 % (**1**) and 85.8 % (**2**) within 5 h	4	[43]
$[PMo^{VI}_{12}O_{40}]^{3-}$/Ti-TUD-1; Ti-TUD-1 = titanium-containing mesoporous silica material	Keggin	T = 70 °C; t = 2 h; m(gas oil) = 40 g; $m(H_2O_2$ 30 % aq) = 14 g; H_2O_2 – oxidant; m(catalyst) = 2 g; solvent – toluene; extraction solvent – methanol; V(MeOH) = 40 mL; $n(H_2O_2)/n(S)$ = 10	68 wt % of sulfur content removed at 70 °C within 2 h	3	[44]
Fe_3O_4@CS@POM; POM = PMo^{VI}_{12} > PW^{VI}_{12} > $P_2W^{VI}_{17}$ > $P_2W^{VI}_{18}$ > SiW^{VI}_{12}, CS = chitosan	Keggin	T = 60 °C; t = 60-90 min; m(catalyst) = 100 mg; model oil ([DBT] = 500 ppm in octane); V(model oil) = 20 mL; $V(H_2O_2$ 30%) = 100 µL; H_2O_2 – oxidant; V(MeCN) = 20 mL; DBT = dibenzothiophene	DBT removal efficiency of 100 % within 90 min (*k* = 0.0761 min^{-1}) for POM = $[PMo_{12}O_{40}]^{3-}$	5	[45]
$[(n\text{-}C_4H_9)_4N)_7H_5Si_2W^{VI}_{18}Cd_4O_{68}]$@PVA; PVA = polyvinyl alcohol	sandwich type	T = 40 °C; model oil (500 ppm of Th or BT in n-heptane); V(model oil) = 50 mL; oxidant – 2:1 H_2O_2/acetic acid; V(oxidant) = 6 mL; m(catalyst) = 0.1 g; t = 2 h; BT = benzothiophene; Th = thiophene	BT and Th removal efficiency of 98 % and 97 % within 2 h, respectively	5	[46]
$[C_3SO_3Hnhm]_3PW^{VI}_{12}O_{40}$; $[C_3SO_3Hnhm]^+$ = organic sulfonic acid-functionalized heterocyclic ammonium cation	Keggin	T = 50 °C; t = 105 min; model oil (DBT in n-octane); n(O)/n(S) = 15; H_2O_2 – oxidant; solvent – DMF; DBT = dibenzothiophene	DBT removal efficiency of 99.4 wt % within 105 min	at least 6	[47]
50-DTA-Mo^{VI}O-TiO_2; $Mo^{VI}O = [Mo^{VI}_6O_{19}]^{2-}$, 50-DTA = 50 % loading of dodecyltrimethylammonium	Lindqvist	T = 60 °C; t = 40 min; model oil ([DBT] = 500 ppm in n-octane); V(model oil) = 5 mL; m(catalyst) = 0.010 g; n(O/S) = 2; H_2O_2 – oxidant; $V(H_2O_2$, 30 %) = 11 µL; DBT = dibenzothiophene	DBT removal efficiency of 100 % within 40 min	7	[48]
$[mim(CH_2)_3COO]_3PW^{VI}$@UiO-66; $[mim(CH_2)_3COO]^+$ = methylimidazolium propionate derivative cation, UiO-66 = zirconium-based metal-organic framework	Keggin	T = 70 °C; t = 60 min; model oil (DBT in n-octane; [S] = 1000 ppm); V(model oil) = 5 mL; m(catalyst) = 40 mg; V(MeCN) = 4.5 mL; n(O/S) = 5; $m(H_2O_2$, 30 %) = 0.09 g; H_2O_2 – oxidant; DBT = dibenzothiophene	DBT removal efficiency of 100 % within 60 min	at least 10	[49]
TBA$[PW^{VI}_{11}]$; TBA = tetrabutylammonium	lacunary Keggin	T = 70 °C; t = 40-190 min; model diesel (BT, DBT, 4-MDBT and 4,6-DMDBT in n-octane, [S] = 2000 ppm); V(model diesel) = 1 mL; $n(H_2O_2/S)$ = 3; p = 1 atm; n(catalyst) = 3 µmol; $n(H_2O_2)$ = 0.24 mmol; H_2O_2 – oxidant; DBT = dibenzothiophene; 4,6-DMDBT = 4,6-dimethyldibenzothiophene; 4-MDBT = 4-methyldibenzothiophene; BT = benzothiophene	complete desulfurization of multicomponent model diesel within 190 min	10	[50]
$5\%[(C_6H_{13})_3PC_{14}H_{29}]_3PMo^{VI}_{12}O_{40}$/g-$C_3N_4$; g-$C_3N_4$ = graphitic carbon nitride	Keggin	T = 60 °C; t = 180 min; n(O/S) = 4; V(model oil) = 5 mL; m(catalyst) = 0.05 g; DBT = dibenzothiophene; 4,6-DMDBT = 4,6-dimethyldibenzothiophene	DBT and 4,6-DMDBT removal efficiency of 100 % and 94.8 % within 180 min, respectively	6	[51]
$[H_3PW^{VI}_{12}O_{40}]/ZrO_2$	Keggin	T = 60 °C; t = 2 h; model oil (DBT ([S] = 500 ppm, in 200 mL petroleum ether); V(model oil) = 20 mL; m(catalyst) = 0.05 g; solvent – MeCN; V(MeCN) = 10 mL; n(O/S) = 4; $V(H_2O_2$, 30 wt % aq) = 64 µL; H_2O_2 – oxidant; DBT = dibenzothiophene	complete removal of DBT within 2 h (*k* = 0.0421 min^{-1})	20	[52]

$[H_3PMo^{VI}_{12}O_{40}]/SiO_2@C$;<br>$SiO_2@C$ = silica (SiO_2) coated or supported on carbon (C) materials	Keggin	T = 40 °C; t = 3 h; model oil (800 ppm of DBT in n-octane); V(model oil) = 2 mL; V(MeCN) = 2 mL; n(H_2O_2, 30 wt %) = 0.15 mmol; H_2O_2 – oxidant; n(catalyst) = 0.002 mmol;<br>DBT = dibenzothiophene	DBT removal efficiency of > 99 % within 3 h	5	[53]
POM/PIL/Gr;<br>POM = $[(NH_4)_3Co(OH)_6Mo^{VI}_6O_{18}]$,<br>PIL = poly(ionic) liquid,<br>Gr = graphene	Anderson-Evans	T = 100 °C; t = 3 h; model oil (DBT, BT and 4,6-DMDBT in decahydronaphtalene, [S] = 500 ppm); V(model oil) = 20 mL; m(catalyst) = 10 mg;<br>DBT = dibenzothiophene; 4,6-DMDBT = 4,6-dimethyldibenzothiophene; BT = benzothiophene	DBT, BT and 4,6-DMDBT removal efficiency of 100 %, 72.5 % and 100 % within 3 h, respectively	6	[54]
$C_3H_4N_2$-$[H_3PW^{VI}_{12}O_{40}]$@CTS;<br>CTS = chitosan	Keggin	T = 35 °C; t = 1 h; $CH_3COOH:H_2O_2$ (v/v = 2:1) as oxidant; V(oxidant) = 6 mL;<br>model oil (DBT, BT and Th in n-heptane, [S] = 500 ppm); V(model oil) = 50 mL; V(MeCN) = 10 mL;<br>DBT = dibenzothiophene; BT = benzothiophene; Th = thiophene	Th, BT and DBT removal efficiency of 96 %, 97 % and 97 % within 1 h, respectively	5	[55]
IMo^{VI}_6@iPAF-1;<br>IMo^{VI}_6 = $[Na_5[IMo^{VI}_6O_{24}]\cdot 3H_2O]$,<br>iPAF-1 = ionic porous aromatic framework	Anderson-Evans	T = 90-100 °C; t = 5-6 h; O_2 – oxidant; m(catalyst) = 10-20 mg; model oil (500 mg/L DBT in 6 mL decalin); V(diesel or gasoline) = 10 mL;<br>DBT = dibenzothiophene	-model oil: DBT removal efficiency of 100 % at 90 °C within 5 h;<br>-real gasoline and diesel: sulfur removal of 99.3 % and 99.4 % at 100 °C within 6 h, respectively	9	[56]
1.5HPA@MOF-199@CA;<br>HPA = $[H_3PMo^{VI}_6W^{VI}_6O_{40}\cdot nH_2O]$,<br>MOF-199 = porous copper-based MOF platform,<br>CA = carbon aerogel	Keggin	T = 40 °C; t = 3 h; O_2 – oxidant; [catalyst] = 1.8 g/L; model oil (thiophene in n-octane, [S] = 1000 ppm); V(model oil) = 50 mL	sulfur removal efficiency of 99.23 % within 3 h<br>(k = 1.655 h^{-1})	10	[57]
Fe_3O_4@NH_2-MIL-101-POM;<br>POM = $[H_3PMo^{VI}_6W^{VI}_6O_{40}]$,<br>$NH_2$-MIL-101 = metal-organic framework with amino groups (NH_2)	Keggin	T = 50 °C; t = 60 min; air flow rate = 1000 L/min; model oil (DBT in n-dodecane, [S] = 2000 ppm); [catalyst] = 0.7-0.8 g/L;<br>DBT = dibenzothiophene	DBT removal efficiency of 100 % in 60 min	15	[58]
Cu-SPOM@PbO@PVA;<br>Cu-SPOM = $Na_{13}[(CuW^{VI}_9O_{34})_2H_3Cu_4(H_2O)_2]\cdot 39H_2O$,<br>PVA = polyvinyl alcohol	lacunary Keggin	T = 35 °C; t = 1 h; model oil (Th (500 ppm), BT (500 ppm) and DBT (500 ppm) in n-heptane); V(model oil) = 50 mL; V(H_2O_2/CH_3COOH, v/v 2:1) = 3 mL; H_2O_2/CH_3COOH – oxidant; real gasoline ([S] = 4996 ppmw); m(catalyst) = 0.1 g;<br>DBT = dibenzothiophene; BT = benzothiophene; Th = thiophene; ppmw = parts per million by weight	Model oil: Th, BT and DBT removal efficiencies of 97 %, 98 % and 98 % within 1 h, respectively;<br>Real gasoline: sulfur removal efficiency of 97 % within 1 h	5	[59]
$[\beta\text{-}SiMo^{VI}_3W^{VI}_9O_{40}]$@1-CCNF;<br>1-CCNF = 1-dimensional carbon chain nanofibers	Keggin	T = 60 °C; t = 2 h; model oils (500, 1000 and 2000 ppm of DBT, BT and 4,6-DMDBT in heptane); m(catalyst) = 10 mg; V(model oil) = 5 mL; V(H_2O_2, 30 wt %) = 50 µL; H_2O_2 – oxidant;<br>DBT = dibenzothiophene; 4,6-DMDBT = 4,6-dimethyldibenzothiophene; BT = benzothiophene	DBT, BT and 4,6-DMDBT removal efficiencies of 99, 89 and 100 % within 2 h, respectively	at least 3	[60]
$[H_3PW^{VI}_{12}O_{40}]/SiO_2$	Keggin	T = 30 °C; t = 100 min; H_2O_2 – oxidant; model oils ([S] = 500 mg/L, DBT, BT or 4,6-DMDBT in petroleum ether); V(model oil) = 10 mL; m(catalyst) = 0.1 g; V(MeCN) = 10 mL; V(H_2O_2, 30 wt %) = 63 µL;<br>DBT = dibenzothiophene; 4,6-DMDBT = 4,6-dimethyldibenzothiophene; BT = benzothiophene	complete removal of DBT within 100 min at 30 °C	6	[61]
SmPOM@TMA-LPMS;<br>SmPOM = $[Sm(PMo^{VI}_{11}O_{39})_2]^{11-}$,<br>TMA-LMPS = trimethylammonium-functionalized (TMA) large-pore mesoporous silica spheres (LMPS)	Keggin sandwich-type	T = 70 °C; t = 1-2 h; n(H_2O_2/S) = 13-16; real diesel ([S] = 23100 ppm); H_2O_2 – oxidant; model diesel (BT, DBT, 4-DMDBT and 4,6-DMDBT in n-octane, [S] =2100 ppm); extractant system – model diesel/[BMIM]PF_6 (v/v = 1:1);<br>DBT = dibenzothiophene; 4,6-DMDBT = 4,6-dimethyldibenzothiophene; BT = benzothiophene; 4-DMDBT = 4-methyldibenzothiophene; [BMIM]PF_6 = 1-	a) Model diesel: complete desulfurization within 1 h;<br>b) Real diesel: sulfur removal of 74 % in 2 h	3	[62]

		butyl-3-methylimidazolium hexafluorophosphate			
[PMoV]@CuO@PAN; PMoV = $K_4[PMo^{VI}_{11}V^{V}O_{40}]$, PAN = polyaniline	Keggin	T = 35 °C; t = 1 h; model oil (500 ppmw of Th, BT and DBT in n-heptane); V(model oil) = 50 mL; 700 rpm; V(oxidant) = 3 mL; oxidant – H_2O_2/acetic acid (v/v = 2:1); solvent – MeCN; m(catalyst) = 0.1 g; V(MeCN) = 10 mL; DBT = dibenzothiophene; BT = benzothiophene; Th = thiophene; ppmw = parts per million by weight	a) Th, BT and DBT removal efficiency of 96 %, 97 % and 97 % respectively b) Real gasoline- sulfur removal efficiency of 96 % within 1 h	5	[63]
$[PW^{VI}_{11}]$@TMA-SBA-15 and $[PW_{11}]$@TMA-PMOE; $[PW_{11}] = [PW^{VI}_{11}O_{39}]^{7-}$, TMA-SBA-15 = SBA-15 aminosilylated mesoporous silica (SBA-15) functionalized with trimethylammonium groups, TMA-PMOE = trimethylammonium-functionalized (TMA) periodic mesoporous organosilica (PMOE)	Keggin	T = 70 °C; t = 60 min; model oil (500 ppm of 1-BT, DBT, 4-MDBT and 4,6-DMDBT in n-octane, [S] = 2000 ppm); n(POM) = 3 µmol; biphasic system: 1:1 of model diesel/MeCN (V = 1.5 mL); oxidant – H_2O_2; V(H_2O_2, 30 %) = 40 µL; n(H_2O_2/S) = 4; solvent-free experiments: V(model diesel) = 750 µL; n(H_2O_2) = 3 µmol; n(H_2O_2/S) = 4; DBT = dibenzothiophene; 4,6-DMDBT = 4,6-dimethyldibenzothiophene; 1-BT = 1-benzothiophene; 4-DMDBT = 4-methyldibenzothiophene	a) Complete conversion of DBT, 4-MDBT and 4,6-DMDBT within 30 min under solvent-free system (both catalyst); for 1-BT: removal efficiency of 99.6 % within 60 min only with PW_{11}@TMA-SBA-15 b) Biphasic system: sulfur removal efficiency of 93.1 % with PW_{11}@TMA-SBA-15	max. 6	[64]
$[PMo^{VI}_{11}Cu]$@$MgCu_2O_4$@CS; CS = chitosan, $[PMo^{VI}_{11}Cu] = [PMo^{VI}_{11}CuO_{40}]^{5-}$	Keggin	T = 35 °C; t = 1 h; m(catalyst) = 0.1 g; oxidant – CH_3COOH/H_2O_2; V(oxidant) = 3 mL; extraction solvent – MeCN; V(solvent) = 10 mL; V(fuel) = 50 mL; DBT = dibenzothiophene; BT = benzothiophene; Th = thiophene	a) DBT, BT and Th removal efficiencies of 99 %, 98 % and 97 % within 1 h, respectively b) Real gasoline – sulfur removal efficiency of 98 %	5	[65]
$[PW^{VI}_{11}Zn]$@aptesSBA-15; aptesSBA-15 = amino-functionalized ((3-aminopropyl)triethoxysilane (aptes)) mesoporous silica (SBA-15), $[PW^{VI}_{11}Zn] = [PW^{VI}_{11}ZnO_{39}]^{5-}$	Keggin	T = 70 °C; t = 60 min; oxidant – H_2O_2; solvent-free: n(H_2O_2/S) = 4; biphasic: n(H_2O_2/S) = 8; model diesel (1-BT, DBT, 4-MDBT and 4,6-DMDBT in n-octane, [S] = 2000 ppm); p = 1 atm, extraction solvent – MeCN; DBT = dibenzothiophene; 4,6-DMDBT = 4,6-dimethyldibenzothiophene; 1-BT = 1-benzothiophene; 4-DMDBT = 4-methyldibenzothiophene	a) Solvent-free: complete desulfurization within 60 min; b) Biphasic system: 97 % of desulfurization within 60 min	5	[66]
$[PMo^{VI}_{12}O_{40}]$@PPy-MSN, $[BPy]_3[PMo^{VI}_{12}O_{40}]$ and $[BMIM]_3[PMo^{VI}_{12}O_{40}]$; PPy-MSN = polypyrrole-coated mesoporous silica nanoparticles, [BPy] = 1-butylpyridinium cation, $[BMIM]^+$ = 1-butyl-3-methylimidazolium cation	Keggin	T = 70 °C; t = 3 h; n(POM) = 3 µmol; oxidant – H_2O_2; V(H_2O_2) = 75 µL; n(H_2O_2)/n(S) = 11; 1:1 model diesel/[BMIM][PF_6] ionic liquid; model oil (1-BT, DBT, 4-MDBT and 4,6-DMDBT in n-octane, [S] = 2350 ppm); DBT = dibenzothiophene; 4,6-DMDBT = 4,6-dimethyldibenzothiophene; 1-BT = 1-benzothiophene; 4-DMDBT = 4-methyldibenzothiophene	sulfur removal efficiency of 98 % within 3 h	3	[67]
(TBA)$[PW^{VI}Fe]$/PVA/CTS; (TBA)PWFe = (*n*-$C_4H_9)_4N)_4[PW^{VI}_{11}Fe(H_2O)O_{39}]$, PVA = polyvinyl alcohol, CTS = chitosan	Keggin	T = 60 °C; t = 2 h; model oil (BT, DBT, 4-MDBT and 4,6-DMDBT in n-heptane, [S] = 500 ppm); V(model oil) = 50 mL; oxidant – H_2O_2/acetic acid (v/v = 1:1); V(oxidant) = 6 mL; m(catalyst) = 0.1 g; extraction solvent – MeCN; V(MeCN) = 10 mL; BT = benzothiophene; DBT = dibenzothiophene; 4,6-DMDBT = 4,6-dimethyldibenzothiophene; 4-DMDBT = 4-methyldibenzothiophene	a) Gas oil: sulfur removal efficiency of 97 % in 2 h b) Model oil: BT, DBT, 4-MDBT and 4,6-DMDBT removal efficiency of 96 %, 98 %, 97 % and 97 %, respectively	5	[68]
$[Ni_2Cl(TMR4A)_2(CH_3CN)_2]\cdot[PMo^{VI}_{12}O_{40}]\cdot4CH_3CN$ and $[Co_2Cl(TMR4A)_2(CH_3CN)_2]\cdot[PMo^{VI}_{12}O_{40}]\cdot4CH_3CN$; TMR4A = resorcin[4]arene-based ligand	Keggin	T = 50 °C; t = 3-14 h; n(catalyst) = 2 µmol; oxidant – TBHP; n(TBHP) = 1 mmol; n(substrate) = 0.4 mmol; V(CH_2Cl_2) = 5 mL; BT = benzothiophene; DBT = dibenzothiophene; 4,6-DMDBT = 4,6-dimethyldibenzothiophene; THBP = tert-butyl hydroperoxide	Removal efficiencies of both catalysts: MBT – 100% conversion within 3 h; DBT – 99 % conversion within 10 h; 4,6-DMDBT – 88 % conversion within 14 h and BT – 69 % within 14 h	5	[69]
		T = 60 °C; t = 120 min; m(catalyst) = 0.02 g; n(O/S) = 2; model fuel (DBT, BT or 4,6-DMDBT in petroleum ether, [S] = 1000			

		μg/g); V(model oil) = 20 mL; V(MeCN) = 20 mL;<br>BT = benzothiophene; DBT = dibenzothiophene; 4,6-DMDBT = 4,6-dimethyldibenzothiophene			
$[PW^{VI}_{11}]$@aptesSBA-15;<br>$[PW^{VI}_{11}] = [PW^{VI}_{11}O_{39}]^{7-}$,<br>aptesSBA-15 = amino-functionalized ((3-aminopropyl)triethoxysilane (aptes)) mesoporous silica (SBA-15)	lacunary Keggin	T = 70 °C; t = 1 h; model diesel (500 ppm of 1-BT, DBT, 4-MDBT and 4,6-DMDBT in n-octane); biphasic system: 1:1 diesel/MeCN; oxidant − H_2O_2; n(O/S) = 8; n(POM) = 3 μmol;<br>real diesel ([S] = 2300 ppm); n(O/S) = 4; t= 2 h;<br>1-BT = 1-benzothiophene; DBT = dibenzothiophene; 4,6-DMDBT = 4,6-dimethyldibenzothiophene; 4-DMDBT = 4-methyldibenzothiophene	a) Model oil – complete desulfurization of within 1 h;<br>b) Real diesel: sulfur removal efficiency of 83.4 % within 2 h	8	[71]
$(DODMAC)_3Co(OH)_6Mo^{VI}_6O_{18}\cdot 3H_2O$;<br>DODMAC = dodecyltrimethylammonium chloride surfactant	Anderson-Evans	T = 90 °C; t = 6 h; oxidant – O_2; flow rate = 60 mL/min; biphasic system – model diesel/$[Opy]BF_4$; model diesel (DBT, 4-DMDBT or 4,6-DMDBT in decalin, [S] = 500 ppm); V(model oil) = 20 mL; m(catalyst) = 10 mg; V($[Opy]BF_4$)/V(model oil) = 1:5;<br>DBT = dibenzothiophene; 4,6-DMDBT = 4,6-dimethyldibenzothiophene; 4-DMDBT = 4-methyldibenzothiophene; $[Opy]^+$ = 1-octylpyridinium cation	complete removal of DBT within 6 h	7	[72]
$K_6[\alpha\text{-}P_2W^{VI}_{18}O_{62}]\cdot 14H_2O$/mGO;<br>mGO = modified graphene oxide	Wells-Dawson	T = 60 °C; m(catalyst) = 0.5 wt %; t = 300 min; model oil (DBT, BT and 4,6-DMDBT in n-octane); V(model oil) = 60 mL; oxidant – O_2; flow rate = 200 mL/min;<br>BT = benzothiophene; DBT = dibenzothiophene; 4,6-DMDBT = 4,6-dimethyldibenzothiophene	sulfur removal efficiency of 96.10 % within 300 min	5	[73]
$(NH_4)_3Co(OH)_6Mo^{VI}_6O_{18}$/*p*-TsOH/PEG4000 (1:2);<br>*p*-TsOH = para-toluenesulfonic acid, PEG4000 = polyethylene glycol with average molecular weight 4000	Anderson-Evans	T = 60 °C; t = 1 h; model diesel ([S] = 500 ppm); V(model diesel) = 20 mL; V(DES) = 4 mL; m(catalyst) = 20 mg; p = 1 atm; oxidant – O_2; flow rate = 60 mL/min; solvent – MeCN;<br>DBT = dibenzothiophene; DES = deep eutectic solvent	DBT removal efficiency of 99 % in 1 h	5	[74]
$[C_7H_7(CH_3)_3N]_9PW^{VI}_9O_{34}$	Keggin	T = 60 °C; m(catalyst) = 0.2015 g; n(O/S) = 10; oxidant – H_2O_2; t = 3 h; model oil (DBT in n-octane; $[S]_0$ = 500 ppm); m(H_2O_2) = 0.749 g;<br>DBT = dibenzothiophene	DBT removal efficiency of 100 % within 3 h	4	[75]
$Fe_2W_{18}Fe_4$@NiO@CTS;<br>$Fe_2W_{18}Fe_4$ = $Na_9K[(FeW^{VI}_9O_{34})_2Fe_4(H_2O)_2]\cdot 32H_2O)$,<br>CTS = chitosan	sandwich-type	T = 35 °C; t = 60 min; model oil (Th, BT and DBT in n-heptane, [S] = 500 ppm); V(model oil) = 50 mL; m(catalyst) = 0.1 g; oxidant − H_2O_2/acetic acid (v/v = 2:1); V(oxidant) = 3 mL; V(MeCN) = 10 mL;<br>Th = thiophene; BT = benzothiophene; DBT = dibenzothiophene	a) Gasoline: sulfur removal efficiency of 97 % within 60 min;<br>b) Model oil: Th, BT and DBT removal efficiencies of 97 %, 98 % and 99 %, respectively	5	[76]
$[PyPS]_3(NH_4)_3Mo^{VI}_7O_{24}$;<br>[PyPS] = pyridylphosphinosulfonate	heptamolybdate	T = 25 °C; m(catalyst) =10 mg;<br>t = 60 min; n(H_2O_2)/n(S) = 3, V(H_2O_2) = 0.053 mL; oxidant – H_2O_2; V_{IL}/V_{Oil} = 1/10; V($[Omim]BF_4$) = 1 mL; model oil (DBT in n-octane);<br>real diesel: V(H_2O_2) = 2 mL; m(catalyst) = 0.2 g; T = 80 °C; t = 4 h;<br>DBT = dibenzothiophene; $[Omim]^+$ = 1-methyl-3-octylimidazolium cation; IL = ionic liquid	a) DBT removal efficiency of 99% within 60 min;<br>b) Real diesel: sulfur removal efficiency of 96 %	5	[77]
POM-PMI_n;<br>POM = $[\beta\text{-}Mo^{VI}_8O_{26}]^{4-}$,<br>$PMI_n$ = poly(2,*p*-methylphenylionene)	octamolybdate	T = 50 °C; t = 2 h; m(catalyst) = 40 mg; model oil (BT, DBT and 4,6-DMDBT in n-octane ([S] = 250, 500 or 1000 ppm)); m(model oil)= 10 g; n(H_2O_2/S) = 5; V(MeCN) = 10 mL; oxidant – H_2O_2;<br>BT = benzothiophene; DBT = dibenzothiophene; 4,6-DMDBT = 4,6-dimethyldibenzothiophene	DBT removal efficiency of 98.9 % within 2 h	6	[78]

3DOM HPW/Al–TiO_2; HPW = [$H_3PW^{VI}_{12}O_{40}$], 3DOM HPW/Al–TiO_2 = three-dimensionally ordered macroporous (3DOM) alumina (Al) doped phosphotungstic acid (HPW)-TiO_2 material	Keggin	T = 60 °C; m(catalyst) = 0.03 g; n(O/S) = 4; t = 1 h; oxidant – H_2O_2; [S] = 500 ppm; V(model oil) = 10 mL; V(MeCN) = 10 mL; DBT = dibenzothiophene	DBT removal efficiency of 99.7 % within 1 h	6	[79]
PMo_{12}@TBA-MSN; PMo_{12} = [$PMo^{VI}_{12}O_{40}$]$^{3-}$, TBA-MSN = tetrabutylammonium-functionalized (TBA) mesoporous silica nanoparticles (MSN)	Keggin	T = 70 °C; t = 2 h; model oil (1-BT, DBT,4-MDBT and 4,6-DMDBT in n-octane, [S] = 2016 ppm); n(catalyst) = 3 µmol; n(O/S) = 13; V(H_2O_2) = 75 µL; oxidant – H_2O_2; extraction solvent − MeCN; 1-BT = 1-benzothiophene; DBT = dibenzothiophene; 4,6-DMDBT = 4,6-dimethyldibenzothiophene; 4-DMDBT = 4-methyldibenzothiophene	complete desulfurization of model oil within 2 h	3	[80]
40 % HPW-GO; 40 % HPW = 40 % (wt %) of [$H_3PW^{VI}_{12}O_{40}$], GO = graphene oxide	Keggin	T = 60 °C; t = 30-60 min; oxidant − H_2O_2; extraction solvent – MeCN; n(O/S) = 6; [catalyst] = 5 g/L; DBT = dibenzothiophene; 4,6-DMDBT = 4,6-dimethyldibenzothiophene	complete removal of DBT and 4,6-DMDBT within 30 min and complete removal of BT within 60 min	8	[81]
TBA-$Si_2W^{VI}_{18}Mn_4$@SAB; TBA-$Si_2W^{VI}_{18}Mn_4$ = (n-C_4H_9)$_4$N)$_7H_5$[($SiW^{VI}_9O_{34}$)$_2$$Mn^{II}_4$($H_2O$)$_2$, SBA = mesoporous silica material	sandwich-type	T = 35 °C; t = 1 h; CH_3COOH/H_2O_2 – oxidant; V(oxidant) = 3 mL; m(catalyst) = 0.1 g; model fuel (DBT, BT and Th in n-heptane, [S] = 500 ppm); V(model fuel) = 50 mL; solvent – MeCN; V(MeCN) = 10 mL; BT = benzothiophene; DBT = dibenzothiophene; Th = thiophene	a) Real gasoline: sulfur removal efficiency of 97 % within 1 h; b) Model fuel: DBT, BT and Th removal efficiency of 98 %, 97 % and 96 %, respectively	5	[82]
[(C_6H_{13})$_3$P($C_{14}H_{29}$)]$_3$$PMo^{VI}_{12}O_{40}$ /ChCl/2Ac; ChCl = choline chloride, Ac = acetate	Keggin	T = 50 °C; t = 120 min; model oil ([S] = 500 ppm in n-octane); V(model oil) = 5 mL; oxidant – H_2O_2; n(catalyst) = 0.0156 mmol; n(O)/n(S) = 4; V(ChCl/2Ac) = 2.5 mL; DBT = dibenzothiophene; 4-MDBT = 4-methyldibenzothiophene; 4,6-DMDBT = 4,6-dimethyldibenzothiophene	DBT, 4-MDBT and 4,6-DMDBT removal efficiencies of 97.2 %, 80.7 % and 76.0 % within 2 h, respectively	5	[83]
HPA–GO; HPA = [$H_3PMo^{VI}_{12}O_{40}$], [$H_3PMo^{VI}_8W^{VI}_4O_{40}$], [$H_3PMo^{VI}_6W^{VI}_6O_{40}$], or [$H_3PW^{VI}_{12}O_{40}$]; GO = graphene oxide	Keggin	T = 50 °C; t = 30 min; catalyst loading: 2.4 g L^{-1}; n(O/S) = 6; extraction solvent – MeCN; [S] = 500 ppm or 1000 ppm	sulfur removal efficiency of 100 % ([S] = 500 ppm) and 97.5 % ([S] = 1000 ppm) within 30 min	nsp	[84]
[C_4mim]$_3$$PMo^{VI}_{12}O_{40}$/$SiO_2$; [$C_4$mim]$^+$ = 1-butyl-3-methylimidazolium cation	Keggin	m(catalyst) = 0.01 g; T = 60 °C; t = 60 min; n(O/S) = 3; model oil (BT, DBT, 4-MDBT and 4,6-DMDBT in n-octane, [S] = 250 ppm); V(model oil) = 5 mL, oxidant − H_2O_2; BT = benzothiophene; DBT = dibenzothiophene; 4,6-DMDBT = 4,6-dimethyldibenzothiophene; 4-DMDBT = 4-methyldibenzothiophene	sulfur removal efficiency of 100 %	7 (7 % activity loss)	[85]
$FeW^{VI}_{11}V$@CTAB-MMT; $FeW^{VI}_{11}V$ = [$Fe^{III}W^{VI}_{11}V^VO_{40}$]$^{7-}$, CTAB-MMT = cetyltrimethylammonium bromide-modified montmorillonite	Keggin	T = 35 °C; t = 1 h; m(catalyst) = 0.10 g; V(oxidant) = 3 mL; oxidant − HOAc/H_2O_2; extraction solvent – MeCN; model oil (Th, BT and DBT in n-heptane, [S] = 500 ppmw); V(gasoline) = 50 mL; BT = benzothiophene; DBT = dibenzothiophene; Th = thiophene; ppmw = parts per million by weight	BT, DBT and Th removal efficiencies of 98 %, 99 % and 97 % within 1 h, respectively gasoline: sulfur removal efficiency of 97 % within 1 h	5	[86]
[$C_n$mim]$_3H_3V^V_{10}O_{28}$/g-BN; n = 8, 12, 16; [$C_n$mim] = 1-cyanomethyl-3-methylimidazolium, g-BN = graphitic boron nitride	decavanadat e	T = 120 °C; t = 4 h; m(catalyst) = 0.08 g; model oil ([S] = 500 ppm); V(model oil) = 40 mL; oxidant – air; flow rate = 100 mL/min; DBT = dibenzothiophene	DBT removal efficiency of up to 99.8 % within 4 h	6 (0.4 % activity loss)	[87]
42 % PTA@MOF-808A; 42 % PTA = 42 % (wt %) loading of [$H_3PW^{VI}_{12}O_{40}$], MOF-808A = zirconium-based metal-organic framework	Keggin	T = 60 °C; t = 30 min; m(catalyst) = 12 mg; extraction solvent – MeCN; V(MeCN) = 2 mL; model oil ([S] = 1000 ppm); V(model oil) = 2 mL; oxidant – H_2O_2; V(H_2O_2) = 21 µL	complete desulfurization within 30 min (k = 0.16 min^{-1})	at least 5	[88]

CNTs@MOF-199-$Mo_{16}V_2$;<br>$Mo_{16}V_2$ = $[H_8P_2Mo^{VI}_{16}V^{V}_2O_{62}\cdot mH_2O)$,<br>CNTs = carbon nanotubes,<br>MOF-199 = copper-based metal-organic framework	Wells-Dawson	T = 80 °C; t = 180 min; m(catalyst) = 0.12 g; model oil (2.87 g DBT in 250 mL n-octane, [S] = 2000 ppm); V(model oil) = 50 mL; oxidant – O_2; flow rate = 1.5 L/min;<br>DBT = dibenzothiophene	DBT removal efficiency of 98.30 % within 180 min	7	[89]
PMo/BzPN-SiO_2;<br>PMo = $[PMo^{VI}_{12}O_{40}]^{3-}$,<br>BzPN-$SiO_2$ = benzyl-modified porous silica (SiO_2)	Keggin	T = 60 °C; t = 3 h; p = ambient pressure; model oil (DBT, BT or 4,6-DMDBT (0.50 mmol) in heptane (10 mL)); n(POM) = 0.0056 mmol; oxidant – 30% H_2O_2; n(H_2O_2) = 1.51 mmol;<br>DBT = dibenzothiophene; 4,6-DMDBT = 4,6-dimethyldibenzothiophene	100 % of DBT and 4,6-DMDBT conversion	nsp	[90]
$[H_8PV^{V}_5Mo^{VI}_7O_{40}]$	Keggin	T = 120 °C; t = 12 h; n(oxalic acid) = 0.5 mmol; n(POM) = 0.5 mmol in 100 mL of H_2O; model oil (0.25 mmol of DBT in 10 mL n-tetradecane); p = 20 bar; oxidant – O_2;<br>DBT = dibenzothiophene	complete desulfurization of model oil within 12 h	at least 5	[91]
$[C_nquin]_4Mo^{VI}_8O_{26}$,<br>n = 2, 5, 8;<br>$[C_nquin]^+$ = *N*-alkylquinolinium cation with alkyl chain lengths n = 2, 5, 8	octamolybdate	T = 60 °C; t = 90 min; n(H_2O_2)/n(DBT) = 4; n(DBT)/n(catalyst) = 100; model oil (500 ppm of S-compounds in n-dodecane); V(model oil) = 5 mL; V($[C_8mim]BF_4$) = 1 mL; $[C_8mim]BF_4$ – ionic liquid phase;<br>DBT = dibenzothiophene; $[C_8mim]^+$ = 1-octyl-3-methylimidazolium cation	DBT removal efficiency of up to 99.2 % within 90 min	5 (4.8 % of activity loss)	[92]
PMo_{12}@MOF;<br>PMo_{12} = $[PMo^{VI}_{12}O_{40}]^{3-}$,<br>MOF = metal-organic framework NH_2-MIL-101(Cr)	Keggin	model diesel (S-compounds ([S] = 2000 ppm) in n-octane); V(model diesel) = 0.75 mL; oxidant – H_2O_2; T = 50 °C; extraction solvent – MeCN or [BMIM][BF_6]; n(POM) = 3 µmol; V(extracting solvent) = 0.75 mL; n(H_2O_2) = 0.30 mmol; t = 2 h;<br>[BMIM] = 1-butyl-3-methylimidazolium	a) Real diesel: sulfur removal efficiency of 80 % within 2 h;<br>b) Model diesel: sulfur removal efficiency of 95 %	3	[93]
POM@MOF-199@LZSM-5;<br>POM = $[H_3PMo^{VI}_6W^{VI}_6O_{40}]$,<br>MOF-199 = copper-based metal-organic framework,<br>LZSM-5 = large pore size zeolite	Keggin	T = 60 °C; t =120 min; [catalyst] = 1.5 g/L; [S] = 2000 ppm; oxidant – O_2; flow rate = 1000 mL/min	complete desulfurization within 120 min	10 (8.96 % of activity loss)	[94]
$[Dda\text{-}pX]_2[\beta\text{-}Mo^{VI}_8O_{26}]$;<br>[Dda-pX] = *N*,*N*-didodecylammonium with a *p*-substituted group "X" on the aromatic or alkyl chain	octamolybdate	T = 40 °C; t = 120 min; m(catalyst) = 40 mg; m(model oil) = 10 g; oxidant – H_2O_2; n(H_2O_2)/n(S) = 6; model oil (DBT in n-octane, [S] = 1000 ppm);<br>DBT = dibenzothiophene	DBT removal efficiency of 99.7 % within 120 min	6	[95]
$[H_3PMo^{VI}_{12}O_{40}]$-GO;<br>GO = graphene oxide	Keggin	T = 50 °C; t = 30 min; [catalyst] = 2.5 g/L; n(O/S) = 6; oxidant – H_2O_2; model oil (DBT in n-hexane, [S] = 500 ppm); V(model oil) = 5 mL;<br>DBT = dibenzothiophene	complete desulfurization within 30 min	6	[96]
ODA_7PW_{11};<br>ODA^+ = octadecylammonium cation,<br>PW_{11} = $[PW^{VI}_{11}O_{39}]^{7-}$	lacunary Keggin	T = 70 °C; t = 40 min; oxidant – H_2O_2; n(H_2O_2)/n(S) = 3 or 8; n(catalyst) = 3 µmol; model diesel ([S] = 500 ppm); V(model diesel) = 0.75 mL	complete desulfurization within 40 min	nsp	[97]
C@$PMo^{VI}_{10}V^{V}_2$;<br>C = carbon composite,<br>$PMo^{VI}_{10}V^{V}_2$ = $[H_5PMo^{VI}_{10}V^{V}_2O_{40}]$	Keggin	T = rt; t = 2 h; m(catalyst) = 1.4 x 10^{-4} g; V(H_2O_2) = 8 µl; solvent – EtOH:n-heptane (v/v = 1:1); model oil ([S] = 250, 500 or 1000 ppm); V(model oil) = 5 mL;<br>rt = room temperature	sulfur removal efficiency of 94 % within 2 h	at least 4	[98]
$Cs_5[PM(H_2O)Mo^{VI}_{11}O_{39}]\cdot 5H_2O$;<br>M = Co^{2+}, Ni^{2+}, Zn^{2+}, and Mn^{2+}	Keggin	T = 60 °C; t = 100 min; model oil (refractory S-compounds in n-octane, [S] = 500 ppm); V(model oil) = 5 mL; solvent – MeCN; V(MeCN) = 5 mL; oxidant – H_2O_2 30 %; n(catalyst) = 4 µmol; n(H_2O_2)/n(DBT) = 8; BT = benzothiophene;<br>DBT = dibenzothiophene; 4,6-DMDBT = 4,6-dimethyldibenzothiophene	for Co^{II}-POM: DBT, 4,6-DMDBT and BT removal efficiencies of 99.8 %, 92.9 % and 85.3 % within 100 min, respectively	at least 4	[99]
$PW_{11}Zn$@$BMIMPF_6$;<br>$PW_{11}Zn$ = $TBA_4H[PW^{VI}_{11}Zn(H_2O)O_{39}]\cdot 5H_2O$,<br>TBA = tetrabutylammonium	Keggin	T = 50 °C; t = 3 h; oxidant – H_2O_2; model oil (DBT, 1-BT and 4,6-DMDBT in n-octane, [S] = 500 ppm of each); biphasic system – model diesel/ $BMIMPF_6$ (v/v = 1:1); V(H_2O_2) = 30 µL;	a) Model diesel: complete desulfurization within 3 h;<br>b) Real diesel: desulfurization	at least 3	[100]

$BMIMPF_6$ = 1-butyl-3-methylimidazolium hexafluorophosphate		1-BT = 1-benzothiophene; DBT = dibenzothiophene; 4,6-DMDBT = 4,6-dimethyldibenzothiophene			
$[cetrimonium]_{11}[P_2W^{VI}_{13}V^{V}_5O_{64}]$	Wells-Dawson	T= 60 °C; t = 60 min; [catalyst] = 7.5 g/L; n(O)/n(S) = 4; oxidant – H_2O_2/formic acid (n(O)/n(acid) = 1); model oil (500 ppmw of DBT and 500 ppmw of BT in isooctane); extracting solvent – MeCN; BT = benzothiophene; DBT = dibenzothiophene; ppmw = parts per million by weight	a) Model oil: DBT and BT removal efficiencies of 98 % and 82 %, respectively<br>b) Real diesel: total sulfur removal efficiency of 90 %	8	[101]
$TBA_4[PW^{VI}_{11}Fe^{III}(H_2O)O_{39}]$@PbO; TBA = tetrabutylammonium	Lacunary Keggin	T = 60 °C; t = 2 h; oxidant – CH_3COOH/H_2O_2 (v/v = 1:1); model oil (aromatic sulfur compounds in n-heptane (BT, DBT, 4-MDBT and 4,6-DMDBT, [S] = 500 ppmw of each compound); V(model oil) = 50 mL; V(oxidant) = 6 mL; m(catalyst) = 0.1 g; V(MeCN) = 10 mL; BT = benzothiophene; DBT = dibenzothiophene; 4-MDBT = 4-methyl dibenzothiophene; 4,6-DMDBT = 4,6-dimethyldibenzothiophene; ppmw = parts per million by weight	a) Real gas oil: sulfur removal efficiency of 97 % after 2 h;<br>b) Model oil: BT, DBT, 4-MDBT and 4,6-DMDBT removal efficiencies of 93 %, 97 %, 94 % and 95 %, respectively	5 (4 % of activity loss)	[102]
$(OTA)_3PW^{VI}_{11}SnO_{39}/TiO_2$; OTA = octadecyltrimethylammonium	Lacunary Keggin	T = 60 °C; t = 20 min; m(catalyst) = 0.02 g; n(O)/n(S) = 6; oxidant – H_2O_2; model oil ([S] = 500 ppm in n-octane); V(model oil) = 5 mL; V($[BMIM]PF_6$) = 1 mL; $[BMIM]PF_6$ = 1-butyl-3-methylimidazolium hexafluorophosphate; DBT = dibenzothiophene	DBT removal efficiency of 100 % within 20 min	7	[103]
$[PSPy]_3[PMo^{VI}_{12}O_{40}]$/GC; [PSPy] = *N*-(3-sulfonatepropyl)-pyridinium, GC = graphite carbon	Keggin	T = 50 °C; t = 60 min; model oil (500 ppmw of DBT in n-octane); V(model oil) = 5 mL; V(H_2O_2, 30 %) = 24 µL; n(O)/n(S) = 3; m(catalyst) = 0.05 g; DBT = dibenzothiophene; ppmw = parts per million by weight	DBT removal efficiency of 100 % within 60 min	6 (2.8 % of activity loss)	[104]
$[Na_3PW^{VI}_{12}O_{40}]$	Keggin	T = 70 °C; t = 30 min; model oil (500 ppm of DBT or BT in toluene); oxidant – H_2O_2; BT = benzothiophene; DBT = dibenzothiophene	DBT removal efficiency of 97.4 % within 30 min<br>(k = 0.4008 min^{-1})	nd	[105]
[VimAm]Br–$PMo^{VI}_6W^{VI}_6O_{40}$@CA; CA = green fiber, [VimAm]Br = 1-vinyl-3-amyl imidazolium bromide	Keggin	T = 60 °C, t = 60 min; [catalyst] = 2.0 g/L; n(O/S) = 7; [S] = 1000 ppm; DBT = dibenzothiophene	DBT removal efficiency of 99.91 % within 60 min	5	[106]
HPW@HUSY; HPW = $[H_3PW^{VI}_{12}O_{40}]$, HUSY = H-type ultrastable Y zeolite	Keggin	T = 333 K; t = 120 min; model oil (500 ppm DBT in n-octane); V(model oil) = 20 mL; n(O/S) = 5; m(catalyst) = 0.1 g; DBT = dibenzothiophene	99.2 % DBT removal within 120 min	6	[107]
Mo_8/h-BN; Mo_8 = $[Mo^{VI}_8O_{26}]^{4-}$, h-BN = hexagonal boron nitride	octamolybdate	T = rt; t = 35 min; n(O/S) = 4; POM loading amount = 50 wt %; V($[BMIM]BF_6$) = 1 mL; [S] = 500 ppm; $BMIMPF_6$ = 1-butyl-3-methylimidazolium hexafluorophosphate; DBT = dibenzothiophene; rt = room temperature	100 % DBT conversion within 35 min	5	[108]
Fe_3O_4@CTS@PMoW; CTS = chitosan, PMoW = $PMo^{VI}_6W^{VI}_6O_{40}$	Keggin	Model oil (5 mL hexane with 500 ppm S-compounds); n(O/S) = 5; T = 60 °C; t = 120 min	99 % S-removal efficiency within 60 min	5	[109]
Fe_3O_4@CTS@HPWV; CTS = chitosan, HPWV = $H_{11}P_2W^{VI}_{13}V^{V}_5O_{62}$	Wells-Dawson		97 % S-removal efficiency within 60 min		
$PMo_{11}V$/NiO/PAN; $PMo_{11}V$ = $H_4PMo^{VI}_{11}V^{V}O_{40}$, PAN = polyaniline	Keggin	T = 35 °C; [S] = 500 ppm; t = 60 min; [catalyst] = 0.10 g; V(H_2O_2, 30 %) = 3 mL; V(MeCN) = 10 mL	97 % S-removal efficiency after 60 min	5	[110]
IL($CoMo_6$)-MIL-101-NH_2; $CoMo_6$ = $(NH_4)_3H_6CoMo^{VI}_6O_{24}$, Metal-organic framework = IL(Br)-MIL-101-NH_2	Anderson-Evans	T = 90 °C; t = 120 min; model oil ([S] = 500 ppm in n-octane); m(catalyst) = 20 mg; O_2 (1 atm, 60 mL/min); DBT = dibenzothiophene	100 % DBT removal efficiency within 120 min	6	[111]

HPW/H-β-TPAOH@TiO2@SiO2-T+S; H-β-TPAOH = tetrapropyl ammonium hydroxide H-β zeolite ($n(SiO_2/Al_2O_3)$ = 20.5), T+S = simultaneous titanium silylation treatment, HPW = $[PW^{VI}_{12}O_{40}]^{3-}$	Keggin	T = 50 °C; t = 1 h; $m_{oil}/m_{catalyst}$ = 35:1; n(O/S) = 13; model oil ([S] = 1100 ppmw in n-octane); DBT = dibenzothiophene; BT = benzothiophene; ppmw = parts per million by weight	99.9 % DBT and BT removal efficiency in 60 min	4	[112]
$PMo_{10}V_2$/APTES-HMSNS; $PMo_{10}V_2$ = $H_5PMo^{VI}_{10}V^{V}_2O_{40}$, APTES-HMSNS = 3-aminopropyltriethoxysilane - hollow mesoporous silica nanomaterials	Keggin	T = 60°C; t = 60 min; [catalyst] = 2.5 g/L; n(O/S) = 10; model oil ([S] = 2000 ppm in n-octane); DBT = dibenzothiophene	99.99 % DBT removal in 60 min	8	[113]
$[C_{12}mim]_3PW^{VI}_{12}O_{40}$/RE-UiO-66; RE = rare-earth metals (Y or La), UiO-66 = Zr-based metal-organic framework, $[C_{12}mim]$ = 1-dodecyl-3-methylimidazolium	Keggin	T = 60 °C; t = 40 min; n(O/S) = 5, m(catalyst) = 0.1 g; model oil ([S] = 500 µg/g); V(model oil) = 10 mL; V(MeCN) = 0.5 mL; DBT = dibenzothiophene	100 % DBT removal efficiency within 40 min	13	[114]
n[$H_3PW^{VI}_{12}$-MIL-101(Cr)]/m(TiO_2), n/m = 1:5.78, MIL-101(Cr) = Cr-based metal-organic framework MIL-101	Keggin	T = 50 °C; n(O/S) = 4; t = 180 min, m(catalyst) = 0.02 g; model oil ([S] = 500 ppm in n-octane)	99 % desulfurization efficiency within 180 min	6	[115]
$[C_{16}mim]PMo^{VI}_{12}O_{40}$/MIL-101; $[C_{16}mim]$ = 1-hexadecyl-3-methylimidazolium, MIL-101 = Cr-based metal-organic framework	Keggin	$[C_{16}mim]Cl$ – ionic liquid; model oil ([S] = 200 ppm in n-octane); V(model oil) = 5 mL; m(catalyst) = 50 mg; T = 50 °C; n(O/S) = 5; DBT = dibenzothiophene	99.8 % DBT removal efficiency	8	[116]
p-C_4VIM-V_{10}; V_{10} = $[V^{V}_{10}O_{28}]^{6-}$ $[C_4VIM]$ = 1-vinyl-3-alkylimidazolium (ionic liquid)	decavanadat e	T = 120 °C, t = 3 h; air flow = 100 mL/min; m(catalyst) = 0.05 g	98.8 % sulfur removal efficiency in 3 h	10	[117]
$P_2W_{18}Co_4$@$ZnFe_2O_4$@PVA PVA = polyviniyl alcohol, $P_2W_{18}Co_4$ = $[(PW^{VI}_9O_{34})_2Co_4(H_2O)_2]^{10-}$	sandwich-type	[S] = 100 ppm; UV lamp: 50 W Hg lamp, λ = 313 nm; t = 90 min; m (catalyst) = 0.03 g; BT = benzothiophene; DBT = dibenzothiophene	97 % BT and 94 % DBT removal efficiency in 90 min	5	[118]
Fe_3O_4@C@P_2W_{18}, P_2W_{18} = $[P_2W^{VI}_{18}O_{62}]^{6-}$, C = carbon composite	Wells-Dawson	T = 70 °C; t = 5 min; n(O/S) = 2; model oil ([S] = 2000 ppm in n-octane); m(catalyst) = 0.03 g	100 % sulfur removal efficiency within 5 min at 70 °C	12	[119]
PDC-PMo_{12}, PDC-Mo_8, and PDC-Mo_6; PMo_{12} = $H_3PMo^{VI}_{12}O_{40}$, Mo_8 = $[(n\text{-}C_4H_9)_4N]_4[\alpha\text{-}Mo^{VI}_8O_{26}]$, Mo_6 = $[(n\text{-}C_4H_9)_4N]_2[Mo^{VI}_6O_{19}]$, PDC = polyionic liquid (prepared from 3-propionic acid-1-vinylimidazolium bromine)	Keggin, Lindqvist and octamolybda te	T = 50 °C; t = 40 min; model oil ([S] = 500 ppm in n-octane); n(O/S) = 4; m(catalyst) = 10 mg; DBT = dibenzothiophene	PDC-PMo_{12}: 14.6 % DBT removal efficiency within 40 min; PDC-Mo_8: 100 % DBT removal efficiency within 40 min; and PDC-Mo_6: 52.4 % DBT removal efficiency within 40 min	5	[120]
$[C_{16}MIM]_4[PW^{VI}_{11}Fe^{III}(H_2O)O_{39}]$; $[C_{16}MIM]$ = 1-hexadecyl-3-methylimidazolium	Keggin	model oil ([S] = 500 ppm); t = 150 min; 1 % catalyst dosage (relative to model oil mass); T = 70 °C; H_2O_2 – oxidant; DBT = dibenzothiophene	98.54 % DBT removal efficiency in 150 min	5	[121]
$[H_2BBPTZ]_2Co(BBPTZ)_2[P_2W^{VI}_{18}O_{62}]\cdot 2H_2O$; BBPTZ = 4,4′-*bis*(1,2,4-triazol-1-ylmethyl)biphenyl	Wells-Dawson	T = 50 °C; t = 8-12 h; V(CH_2Cl_2) = 5 mL; n(DBT) = 0.3 mmol; n(catalyst) = 0.075 mmol; n(tert-butyl hydroperoxide) = 2 mmol; DBT = dibenzothiophene	94.6 % DBT removal efficiency within 8 h	6	[122]
$[(Ni_2(TBTZ)_2(H_2O)_4][H_2P_2W^{VI}_{18}O_{62}]\cdot 17.5H_2O$; TBTZ = 1,3,5-*tris*(1,2,4-triazol-1-ylmethyl)-2,4,6-trimethyl benzene			98.3 % DBT removal efficiency within 8 h		

*nsp- not specified by authors

2. POMs and POM-based composites in the removal of various organic pollutants from water

Table S2 A Summary of published POMs and POM-based composites for application in the removal of various organic pollutants from water.

Formula	POM archetype	Pollutants	Conditions	Efficiency	Number of cycles	Ref
SiO_2@[*n*-$C_7H_{15})_4N]_8SiW^{VI}_{11}O_{39}^{8-}$	Keggin	PBV	[PBV] = 32 µM; amount of catalyst = 800 mg;<br>PBV = dye patent blue V	95 %	nsp	[123]
PTMS-treated alumina/PEI-$PV^{V}_2Mo^{VI}_{10}O_{40}$;<br>PTMS = 3-aminopropyl trimethoxysilane,<br>PEI = polyetherimide	Keggin	RB5	[RB5] = 20 ppm; T = 25 °C; Operating time = up to 3 h;<br>RB5 = reactive black 5	100 %	6	[124]
$K_6P_2W^{VI}_{18}O_{62}$@UiO-66;<br>UiO-66 = Zr-based metal-organic framework	Wells-Dawson	RhB	[RhB] = 30 ppm; pH = 3; T = 25 °C;<br>RhB = rhodamine B	99 % within 120 min,<br>(k = 0.00438 g mg^{-1} min^{-1})	nsp	[125]
PW_{12}@MIL-101(Fe);<br>$PW_{12} = [PW^{VI}_{12}O_{40}]^{3-}$,<br>MIL-101(Fe) = Fe-based metal-organic framework	Keggin	MB	[MB] = 5 ppm; amount of catalyst = 10 mg/100 mL;<br>MB = methylene blue	99.5 % within 30 min	3	[126]
$[Cu_2(btx)_2(C_2O_4)][H_2SiW^{VI}_{12}O_{40}]\cdot12H_2O$;<br>btx = 1,4-bis(triazol-1-ylmethyl)benzene)	Keggin	MB	nsp;<br>MB = methylene blue	78 % within 60 min, under visible light;<br>91 % within 60 min, under UV light	nsp	[127]
$[\{Cl_4Cu_{10}(pz)_{11}\}\{As_2W^{VI}_{18}O_{62}\}]\cdot1.5H_2O$;<br>pz = pyrazine	Wells-Dawson	RhB	[RhB] = 10^{-5} M; amount of catalyst = 50 mg; light source = 250 W Hg lamp; RhB = rhodamine B	96.8 % within 80 min,<br>(k = 0.005 min^{-1})	5	[128]
$(CH_3NH_2CH_3)[Cu_2(TPB)_2(PW^{VI}_{12}O_{40})]\cdot4DMF\cdot6H_2O$;<br>TPB = 1,2,4,5-tetra(4-pyridyl)benzene	Keggin	MO, MB	[dye] = 10 ppm; amount of adsorbent = 50 mg; light source = 300 W Xe lamp; H_2O_2 = 2 ml (30 %);<br>MB = methylene blue; MO = methyl orange	> 95 % MB removal within 120 min;<br>98.8 % MO removal within 120 min under visible light	nsp	[129]
$[NaP_5W^{VI}_{30}O_{110}]^{14-}$/MIL-101(Cr);<br>MIL-101(Cr) = Cr-based metal-organic framework	Preyssler	MB	[MB] = 50 ppm; amount of catalyst = 30 mg; pH = 2;<br>MB = methylene blue	100 % within 8 min,<br>(k = 1.19 g mg^{-1} min^{-1})	4	[130]
$[P_2W^{VI}_{18}O_{62}]^{6-}/CoFe_2O_4$/MIL-101(Cr);<br>MIL-101(Cr) = Cr-based metal-organic framework	Wells-Dawson	MB	[MB] = 100 ppm; amount of catalyst = 30 mg; pH = 6; T = 25 °C;<br>MB = methylene blue	100 %	3	[131]
$H_6P_2W^{VI}_{18}O_{62}@Cu_3(BTC)_2$;<br>BTC = trimesic acid	Wells-Dawson	MB	[MB] = 10 ppm; amount of catalyst = 20 mg/mL;<br>MB = methylene blue	80 % within 60 min,<br>(k = 0.0047 g mg^{-1} min^{-1})	nsp	[132]
$[PMo^{VI}_{12}O_{40}]@[Cu^{II}_6O(TZI)_3(H_2O)_9]_4\cdot OH\cdot31H_2O$ (**HLJU-1**)<br>$[SiMo^{VI}_{12}O_{40}]@[Cu^{II}_6O(TZI)_3(H_2O)_9]_4\cdot32H_2O$ (**HLJU-2**)<br>$[PW^{VI}_{12}O_{40}]@[Cu^{II}_6O(TZI)_3(H_2O)_6]_4\cdot OH\cdot31H_2O$ (**HLJU-3**);<br>H_3TZI = 5-tetrazolylisophthalic acid	Keggin	RhB, CV	[RhB] = 9.5 ppm; [CV] = 15 ppm; amount of catalyst = 50 mg;<br>RhB = rhodamine B; CV = crystal violet	> 60 % RhB removal and CV for HLJU-3, 80 % < for HLJU-1 and > 95 % for HLJU-2 within 360	5	[133]
$H_6P_2W_{18}O_{62}$/MOF-5;<br>MOF-5 = Zn-based metal-organic framework	Wells-Dawson	MB	[MB] = 20 ppm; amount of catalyst = 15 mg/20 mL;<br>MB = methylene blue	100 % within 10 min,<br>(k = 0.2953 g mg^{-1} min^{-1})	nsp	[134]
FcSiW,<br>Fc = ferrocene,<br>SiW = $H_4SiW^{VI}_{12}O_{40}\cdot xH_2O$, x = nsp	Keggin	4-CP	amount of catalyst = 0.1 g L^{-1}; [H_2O_2] = 10 mM; [4-CP] = 50 mg L^{-1}; pH = 6.5; light source = 250 W Hg lamp; T = 35 °C;<br>4-CP = 4-chlorophenol	100 % within 100 min under UV light; 97 % within 100 min in dark	3	[135]
[illegible]	[illegible]	[illegible]	amount of catalyst = 50 mg; [Phenol] = 10	86.6 % within 300 min,	[illegible]	[illegible]

			ppm; light source = 300 W Xe lamp; O_2 pressure = 0.1 MPa	(k = 0.0065 min^{-1})		
$[Ag_4(H_2pyttz\text{-}I)(H_2pyttz\text{-}II)(Hpyttz\text{-}II)][HSiW^{VI}_{12}O_{40}]\cdot 4H_2O$ (**1**)<br>$[Ag_4(H_2pyttz\text{-}II)(Hpyttz\text{-}II)_2][H_2SiW^{VI}_{12}O_{40}]\cdot 3H_2O$ (**2**);<br>H_2pyttz-I = 3-(pyrid-2-yl)-5-(1H-1,2,4-triazol-3-yl)-1,2,4-triazolyl;<br>H_2pyttz-II = 3-(pyrid-4-yl)-5-(1H-1,2,4-triazol-3-yl)-1,2,4-triazolyl	Keggin	RhB	[RhB] = 10^{-5} M; amount of catalyst = 50 mg; light source = 125 W Hg lamp;<br>RhB = rhodamine B	63.3 % removal for (**1**) within 150 min,<br>(k = 0.006 min^{-1});<br>81 % removal for (**2**) within 150 min,<br>(k = 0.1 min^{-1})	nsp	[137]
per-6-deoxy-6-ethylenediamine-β-cyclodextrin/$H_3PW^{VI}_{12}O_{40}$	Keggin	RhB, MO, MB, XO, CV, NFZ, TCY, BE	[dye/antibiotic] = 1 mM; amount of catalyst = 0.055 mM POM + 0.03 mM EDA-CD; [H_2O_2] = 50 µL; light source = 50 W Hg lamp;<br>RhB = rhodamine B; MB = methylene blue; MO = methyl orange; XO = xylenol orange; CV = crystal violet; NFZ = nitrofurazone; TCY = tetracyclines; BE = berberine	> 95 % removal efficiency of RhB within 4 min,<br>(k = 0.868 ± 0.061 min^{-1});<br>> 95 % removal efficiency of XO within 15 min,<br>(k = 0.214 ± 0.023 min^{-1});<br>> 95 % removal efficiency of MO within 20 min,<br>(k = 0.164 ± 0.016 min^{-1});<br>> 95 % removal efficiency of MB within 30 min,<br>(k = 0.119 ± 0.002 min^{-1});<br>> 95 % removal efficiency of CV within 35 min,<br>(k = 0.084 ± 0.003 min^{-1});<br>> 95 % removal efficiency of NFZ within 19 min,<br>(k = 0.163 ± 0.016 min^{-1});<br>> 95 % removal efficiency of TCY within 25 min,<br>(k = 0.152 ± 0.016 min^{-1});<br>> 95 % removal efficiency of BE within 30 min,<br>(k = 0.115 ± 0.007 min^{-1})	nsp	[138]
$[PW^{VI}_{12}O_{40}]^{3-}$	Keggin	lindane	[lindane] = 2.4 × 10^{-5} M; amount of catalyst = 7×10^{-4} M; pH = 1	100 % within 10 h	nsp	[139]
Fe_2O_3@SiO_2@$[n\text{-}C_7H_{15})_4N]_8SiW^{VI}_{11}O_{39}{}^{8-}$	Keggin	PBV	[PBV] = 32 µM; amount of catalyst = 50 mg;<br>PBV = dye patent blue V	99 % within 24 h	nsp	[140]
$\{HCu^{II}$(N,N′-bis(2-pyrazinecarboxamide)-1,2-ethane)$[Cr^{III}Mo^{VI}_6(OH)_6O_{18}]\}\cdot 4H_2O$ (**1**)<br>$[Cu^{II}_3$(N,N′-bis(2-pyrazinecarboxamide)-1,2-ethane$)_{0.5}(Te^{VI}Mo^{VI}_6O_{24})(H_2O)_9]$ (**2**)	Anderson-Evans	GV, MB, TB, MV	[dye] = 10 ppm; amount of catalyst = 50 mg; T = room temperature;<br>GV = gentian violet; MB = methylene blue; TB = toluidine blue dye; MV = methylene violet	99 % GV removal for (1) and 71.43 % for (**2**);<br>95.63% MB removal for (**1**) and 81.25 % for (**2**);<br>90.65 % TB removal for (**1**) and 77.84 % for (**2**);<br>65.79 % MV removal for (**1**) and 44.87 % for (**2**)	4	[141]
$[(NH_4)_6(Mo^{VI}_7O_{24})]\cdot 4H_2O$	heptamolybdate	MB	[MB] = 220 ppm; amount of catalyst = 50 mg; pH = 1; T = 303 K;<br>MB = methylene blue	> 97 % MB removal within 60 min,<br>*(k = 0.000234 min^{-1})*	nsp	[142]
poly-[N,N-dimethyl-dodecyl-(4-vinylbenzyl)ammonium chloride]/($[Mo^{VI}_8O_{26}]^{4-}$)	octamolybdate	AR87	[AR87] = 200 ppm; T = 20 °C;<br>AR87 = acid red 87	> 98 %	5	[143]
$\{HCu^{II}(HPCAP)_2[Cr^{III}Mo^{VI}_6(OH)_6O_{18}]\}\cdot 2H_2O$ (**1**)<br>$\{Zn_4(PCAP)_2[Cr^{III}Mo^{VI}_6(OH)_6O_{18}]_2(H_2O)_{12}\}\cdot 4H_2O$ (**2**)<br>$\{Zn_3(PCAP)_2[Cr^{III}Mo^{VI}_6(OH)_5O_{19}](H_2O)_6\}\cdot 6H_2O$ (**3**)<br>$\{Ni^{II}_3(PCAP)_2[Ni^{II}Mo^{VI}_6(OH)_5O_{19}](H_2O)_6\}\cdot 8H_2O$ (**4**)<br>$\{Cu^{II}_3(PCAP)_2[AlMo^{VI}_6(OH)_5O_{19}](H_2O)_6\}\cdot 6H_2O$ (**5**)	Anderson-Evans	GV, MB	[dye] = 10 ppm; amount of catalyst = 50 mg;<br>GV = gentian violet; MB = methylene blue	96.9 % GV removal for (**1**), 97.9% for (**2**), 97.3 % for (**3**), 96.6 % for (**4**), 98.6 % for (**5**) and 97.1 % for (**6**),<br>87.3 % MB removal for (**1**), 92.1 % for (**2**), 93.8 % for (**3**), 94.4 %	nsp	[144]

$\{Co^{II}_3(HPCAP)_2[AlMo^{VI}_6(OH)_6O_{18}](H_2O)_{10}\}[AlMo^{VI}_6(OH)_6O_{18}]\cdot 6H_2O$ (**6**); HPCAP = 3-(2-pyridinecarboxylic acid amido)pyridine						
$[Ni^{II}(2,2'\text{-biimidazole})_3]_2[\beta\text{-}Mo^{VI}_8O_{26}]\cdot 8DMF$ (**1**) $(\text{dimethyl-ammonium})_2[Ni^{II}(2,2'\text{-biimidazole})_2(H_2O)_2][\beta\text{-}Mo^{VI}_8O_{26}]\cdot 4DMF$ (**2**) $(\text{dimethyl-ammonium})_2[Co^{II}(2,2'\text{-biimidazole})_2(H_2O)_2][\beta\text{-}Mo^{VI}_8O_{26}]\cdot 4DMF$ (**3**) $[Zn(2,2'\text{-biimidazole})(DMF)_3]_2[\beta\text{-}Mo^{VI}_8O_{26}]\cdot 2DMF$ (**4**) $[Cu^{II}(2,2'\text{-biimidazole})(DMF)_3]_2[\beta\text{-}Mo^{VI}_8O_{26}]\cdot 2DMF$ (**5**)	octamolybdate	MB	[MB] = 10 ppm; amount of catalyst = 15 mg; MB = methylene blue	> 80 % for compounds (**2**)-(**5**); < 10 % for (**1**) within 10 min	3	[145]
$Fe_3O_4@[Ni^{II}(2\text{-acetylpyridine-thiosemicarbazone})_2]_2H_2[P_2Mo^{VI}_5O_{23}]\cdot 4H_2O$ (**1**) $Fe_3O_4@[2\text{-acetylpyridine-thiosemicarbazone}]_5H[P_2Mo^{VI}_5O_{23}]\cdot 12H_2O$ (**2**)	Strandberg	MO, MB	[dye] = 15 ppm; amount of catalyst = 25 mg; MB = methylene blue; MO = methyl orange	94.8 % MB removal for (1) within 240 min, 97.67 % for (**2**) within 60 min; 13.13 % MO removal for (**1**) within 240 min and 8.84 % for (**2**) within 60 min	7	[146]
$[N(C_4H_9)_4]_3[Mn^{II}Mo^{VI}_6O_{18}\{(OCH_2)_3CN = CHC_6H_4OH\}_2]$/hexachlorocyclotriphosphazene	Anderson-Evans	AOG, PS, BF, MB	[MB] = 100 ppm; amount of catalyst = 0.125 mgL^{-1}; AOG = acid orange G; PS = ponceau S; BF = basic fuchsin; MB = methylene blue	98 % MB removal within 240 min, < 2 % AOG and PS removal and 95 % BF removal	nsp	[147]
$H_6P_2W^{VI}_{18}O_{62}$@Cu-BTC; BTC = trimesic acid	Wells-Dawson	TBBPA	[TBBPA] = 2 ppm; amount of catalyst = 40 mg; T = 298 K; TBBPA = tetrabromobisphenol-A	95 %, (k = 5.56 g mg^{-1} min^{-1})	6	[148]
aminopropylsilanized-$Co_3O_4/H_3PW^{VI}_{12}O_{40}$	Keggin	RhB, MO, MB	[dye] = 25 ppm; amount of catalyst = 20 mg; RhB = rhodamine B; MB = methylene blue; MO = methyl orange	98 % MB removal within 12 min, (k = 0.037 g mg^{-1} min^{-1}); 20 % RhB removal within 32 min; the removal efficiency of MO was negligible	3	[149]
$NH_2\text{-}Fe_3O_4/[Cu^{II}(pca)_2(SiW^{VI}_{12}O_{40})](py)_2$; pca = pyridine-2-carboxylic acid; py = pyrazine	Keggin	TCY	pH = 6.8, TCY = tetracycline	88.6 %	5	[150]
$LaMnO_3@SiO_2/PMo^{VI}_{12}$ (**1**) $LaMnO_3@SiO_2/PW^{VI}_{12}$ (**2**) $LaMnO_3@SiO_2/SiW^{VI}_{12}$ (**3**)	Keggin	MB	[MB] = 25 ppm; amount of catalyst = 25 mg; T = 25 °C; MB = methylene blue	100 % removal for (**1**) within 1 min, 98 % for (**2**) within 30 min and 100 % for (**3**) within 0.5 min	3	[151]
$LaNiO_3@SiO_2/PW^{VI}_{12}$	Keggin	MB	[MB] = 25 ppm; amount of catalyst = 30 mg; T = 25 °C; MB = methylene blue	98.5 % within 60 min, (k = 0.066 g mg^{-1} min^{-1})	3	[152]
m-phenylenediamine/$P_5W^{VI}_{30}$	Preyssler	MB	[MB] = 20 ppm; amount of catalyst = 5 mg; MB = methylene blue	> 95 % within 15 min	nsp	[153]
$[Cd(\text{pyridine-2-carbaldehyde semicarbazone})_6][\text{pyridine-2-carbaldehyde semicarbazone}]^+_4[PMo^{VI}_{12}O_{40}]_4\cdot 18MeOH\cdot 4H_2O$	Keggin	RhB, MB	[dye] = 25 ppm; amount of catalyst = 30 mg; T = 25 °C; RhB = rhodamine B; MB = methylene blue	98 % MB removal within 5 min and 86 % RhB removal within 5 min	3	[154]
$CuS@PANI/PW^{VI}_{12}$ (**1**) $CuS@PANI/PMo^{VI}_{12}$ (**2**) $CuS@PANI/SiW^{VI}_{12}$ (**3**); PANI = polyaniline	Keggin	MB	[MB] = 25 ppm; amount of catalyst = 25 mg; pH = 6; T = 25 °C; MB = methylene blue	93 % removal efficiency for (**1**) within 20 min (k = 0.0036 g mg^{-1} min^{-1}), 94 % for (**2**) within 0.5 min and 100 % for (**3**) within 2 min	4	[155]
amide-functionalized *N*-dodecyl-*N'*-acetamideimidazolium bromide/PW^{VI}_{12}	Keggin	RhB	[RhB] = 0.25 mM; amount of catalyst = 0.2 mg/mL; T = 25 °C; RhB = rhodamine B	100 % within 1 min	5	[156]

PW^{VI}_{12}/BEA zeolite; BEA = microporous crystalline aluminosilicate (zeolite)	Keggin	nicosulfuron	PW_{12}/zeolite weight ratio = 20 %; UltraSound = 30 min	adsorption capacity = 25.5 mg g^{-1}	nsp	[157]
POM−GA; POM = HSiW, GA = 3D graphene aerogels	Keggin	various water-soluble organic pollutants	reduction in N_2H_4; POM content = 30 % wt	absorption capacities = 100-210 g g^{-1}	10	[158]
$H_3K_2[Ag_5(DTB)_5][SiW^{VI}_{12}O_{40}]_2\cdot Cl_2\cdot 8H_2O$; DTB = 1,4-di(1H-1,2,4-triazol-1-yl)benzene	Keggin	MB, BY, CV, Rh6G, RhB, EB*, CFB, MO	amount of catalyst = 20 mg; MB = methylene blue; BY = basic yellow 1; CV = crystal violet; Rh6G = rhodamine 6G; RhB = rhodamine B; EB*= eosin B; CFB = chromotrope FB; MO = methyl orange	74.33 % removal efficiency of CV; 84.94 % removal efficiency of MB; 40.51 % removal efficiency of BY; 30.23 % removal efficiency of Rh6G; 12.91 % removal efficiency of RhB; < 10 % removal efficiency for EB*, CFB, and MO within 90 min	nsp	[159]
NiAl-$SiW^{VI}_{12}O_{40}^{4-}$	Keggin	MG	[MG] = 12 ppm; amount of catalyst = 50 mg; MG = malachite green	94 % within 120 min, (*k* = 0.0218 g/mg min)	5	[160]
DODA·Br-$PV^{V}_2Mo^{VI}_{10}$/PVDF; DODA·Br = dimethyldioctadecylammonium bromide, $PV^{V}_2Mo^{VI}_{10}$ = $H_5[PV^{V}_2Mo^{VI}_{10}O_{40}]$, PVDF = polyvinylidene fluoride	Keggin	RB5	[RB5] = 15 ppm; amount of catalyst = 26 mg; T = 45 °C; RB5 = reactive black 5	97.5 % within 120 min	3	[161]
amine-functionalized graphene oxide/$PTi_2W^{VI}_{10}O_{40}^{7-}$	Keggin	RhB, MB	[dye] = 100 ppm; amount of catalyst = 10 mg; RhB = rhodamine B; MB = methylene blue	adsorption capacity of MB = 1095 mg g^{-1}; adsorption capacity of RhB = 540 mg g^{-1}	5	[162]
ZnAlFe-P_2W_{17} (**1**) ZnAlFe-CoW_{12} (**2**); P_2W_{17} = $[P_2W^{VI}_{17}]^{10-}$, CoW_{12} = $[CoW^{VI}_{12}]^{5-}$	Wells-Dawson, Keggin	MB	pH = 6.3; light source = 25 W Xe lamp; MB = methylene blue	< 10 % removal efficiency for (**1**) within 6 h and > 90 % for (**2**) within 6 h	nsp	[163]
$[C_{16}H_{33}(CH_3)_3N]H_4PMo^{VI}_{10}V^{V}_2O_{40}$	Keggin	DEP	[DEP] = 0.45 mM; amount of catalyst = 3.0 mM; $[H_2O_2]$ = 0.014 M; T = 25 °C; pH = 7.0; DEP = diethyl phthalate	90.2 % within 30 min	10	[164]
FePW/LDH (**1**) MnPW/LDH (**2**); LDH = layered double hydroxide, FePW = $[PFe^{III}W^{VI}_{11}O_{39}]^{4-}$, MnPW = $[PMn^{II}W^{VI}_{11}O_{39}]^{6-}$	Keggin	AR27	[AR27] = 20 ppm; amount of catalyst = 0.5 g/L; pH = 3; $[H_2O_2]$ = 0.2 mL/L; T = 40 °C; AR27 = acid red 27	98 % removal efficiency of AR27 for (**1**) within 30 min, and 99 % for (**2**) within 30 min	4	[165]
$PW^{VI}_{12}O_{40}^{3-}$-γ-Fe_2O_3/$SrCO_3$	Keggin	IBP	[IBP] = 10 ppm; amount of catalyst = 50 mg; light source = sunlight; IBP = ibuprofen	nsp	3	[166]
$KH[SiW^{VI}_{12}O_{40}][Ni^{II}(H_2O)_6]$cucurbit[6]uril·$7H_2O$	Keggin	MO	[MO] = 10 ppm; amount of catalyst = 0.5 gL^{-1}; pH = 2.5; $[H_2O_2]$ = 1.5 mmol L^{-1}; MO = methyl orange	95.6 % within 120 min	4	[167]
$Cs_3PMo^{VI}_{12}O_{40}$	Keggin	BR46	[BR46] = 10 ppm; amount of catalyst = 2 gL^{-1}; $[H_2O_2]$ = 2 mM; light source = 300 W Xe lamp; BR46 = basic red 46	100 % within 90 min	3	[168]
{[(Cu₄Cl)(4-(4-carboxyphenyl)-1,2,4-triazolate)₄]·($HSiW^{VI}_{12}O_{40}$)·31H_2O}	Keggin	RhB	[RhB] = 10 ppm; amount of catalyst = 15 mg; $[H_2O_2]$ = 2 mL (30%); light source = 300 W Xe lamp; RhB = rhodamine B	99 % within 80 min	3	[169]
$Na_3PW^{VI}_{12}O_{40}$/D201 resin; D201 = type I anion exchange resin	Keggin	RhB	[RhB] = 2 x 10^{-5} M; $[H_2O_2]$ = 2 x 10^{-3} M; pH = 2.5; light source = 500 W halogen lamp; RhB = rhodamine B	99 % within 240 min	7	[170]
$K_3PW^{VI}_{12}O_{40}$	Keggin	RhB	[RhB] = 2 x 10^{-5} M; amount of catalyst = 0.5 gL^{-1}; $[H_2O_2]$ = 2 x 10^{-3} M; pH = 2.1; light source = 500 W halogen lamp; RhB = rhodamine B	100 % within 150 min	7	[171]

$\{[Cu(en)_2]_{1.5}[Cu(en)(2,2'\text{-}bipy)(H_2O)_n]Ce[(\alpha\text{-}PW^{VI}_{11}O_{39})_2]\}^{6-}$ (**1**)<br>$\{[Cu(en)_2]_{1.5}[Cu(en)(2,2'\text{-}bipy)(H_2O)_n]Pr[(\alpha\text{-}PW^{VI}_{11}O_{39})_2]\}^{6-}$ (**2**)<br>$\{[Cu(en)_2]_2(H_2O)[Cu(en)(2,2'\text{-}bipy)]Gd[(\alpha\text{-}HPW^{VI}_{11}O_{39})_2]\}^{4-}$ (**3**)<br>$\{[Cu(en)_2]_2(H_2O)[Cu(en)(2,2'\text{-}bipy)]Tb[(\alpha\text{-}HPW^{VI}_{11}O_{39})_2]\}^{4-}$ (**4**)<br>$\{[Cu(en)_2]_2(H_2O)[Cu(en)(2,2'\text{-}bipy)]Er[(\alpha\text{-}HPW^{VI}_{11}O_{39})_2]\}^{4-}$ (**5**)<br>$\{[Cu(en)_2]_{1.5}[Cu(en)(2,2'\text{-}bipy)]Nd[(\alpha\text{-}H_5PW^{VI}_{11}O_{39})_2]\}^{3-}$ (**6**);<br>2,2′-bipy =2,2′-bipyridine;<br>en = ethylenediamine	Lacunary Keggin	RhB	[RhB] = 2 x 10^{-5} M; amount of catalyst = 2 x 10^{-6} mol; light source = 500 W Hg lamp; RhB = rhodamine B	26 % removal efficiency for (**1**), 34 % for (**2**), 29 % for (**3**), 35 % for (**4**) and 46 % for (**5**)	nsp	[172]
$H_3PW^{VI}_{12}O_{40}/ZrO_2$	Keggin	BCG, RhB, MO, MB, CV, 4-nitrophenol, DCP	[dye] = 10^{-5} M; amount of catalyst = 25 mg for dye degradation and 40 mg for herbicides; PW_{12}/ZrO_2 weight ratio = 1/3; light source = 50 W Hg lamp;<br>BCG = bromo cresol green; RhB = rhodamine B; MO = methyl orange; MB = methylene blue; CV = crystal violet; DCP = 2,4- dichlorophenoxy acetic acid	78 % removal efficiency of MB within 70 min<br>(k = 0.0218 min^{-1});<br>99 % removal efficiency of RhB within 80 min<br>(k = 0.0456 min^{-1});<br>82 % removal efficiency of MO within 60 min<br>(k = 0.0261 min^{-1});<br>89 % removal efficiency of CV within 50 min<br>(k = 0.0342 min^{-1});<br>73 % removal efficiency of BCG within 20 min;<br>90 % removal efficiency of 4-nitrophenol within 90 min<br>(k = 0.02373 min^{-1});<br>85 % removal efficiency of DCP within 120 min<br>(k = 0.015 min^{-1})	3	[173]
$[Ag(bbi)][\{Ag(bbi)\}_4\{Ag_3(V^{V}{}_4O_{12})_2\}]\cdot 2H_2O$;<br>bbi = 1,1′-(1,4-butanediyl)bis(imidazole)	octavanadate	MB	[MB] = 10 ppm; amount of catalyst = 150 mg; light source = 125 W Hg lamp;<br>MB = methylene blue	70 % within 90 min	5	[174]
$\{(H_2O)_2[Cu_8(\mu_4\text{-}OH)_6Cu_6(H_2O)_6(cpt)_{12}](SiW^{VI}{}_{12}O_{40})_3(EtOH)_4(H_2O)_7\}$;<br>Hcpt = 4-(4′-carboxyphenyl)-1,2,4-triazole	Keggin	MB, RhB	[dye] = 10^{-5} M; amount of catalyst = 20 mg; light source = Xe lamp;<br>MB = methylene blue; RhB = rhodamine B	94.3 % removal efficiency of MB within 50 min;<br>85.4 % removal efficiency of RhB within 70 min	nsp	[175]
$H_3PMo^{VI}_{12}O_{40}$@MOG-Cr;<br>MOG-Cr = metal-organic gel	Keggin	MB, RhB, MO	[dye] = 10 ppm; amount of catalyst = 10 mg; light source = 50 W Xe lamp;<br>MB = methylene blue; RhB = rhodamine B; MO = methyl orange	99 % removal efficiency of MB within 60 min;<br>97 % removal efficiency of RhB within 60 min and 91 % removal efficiency of MO within 120 min	3	[176]
Fe-$PW^{VI}_{12}O_{40}/TiO_2$	Keggin	BPA	[BPA] = 50 ppm; amount of catalyst = 50 mg;<br>BPA = bisphenol A	100 % within 24 min	4	[177]
$H_3PW^{VI}_{12}O_{40}/TiO_2$	Keggin	CR, MO, PG, OII, EB, AS, MB, NR, RhB, FA	[dye] = 50 ppm; amount of catalyst = 250 mg; light source = 400 W Xe lamp;<br>CR = Congo red; MO = methyl orange; PG = Ponceau G; OII = orange II; EB = eriochrome blue black B; AS = alizarin S;<br>MB = methylene blue; NR = neutral red;	92 % removal efficiency of CR within 120 min;<br>72.4 % removal efficiency of MO within 240 min;<br>94.8 % removal efficiency of PG within 180 min;<br>67.2 % removal efficiency of OII within 240 min;<br>75.8 % removal efficiency of EB within 180 min;<br>72.8 % removal efficiency of AS within 240 min;	nsp	[178]

				96 % removal efficiency of MB within 60 min; 98.2 % removal efficiency of NR within 60 min; 98 % removal efficiency of RhB within 60 min; 75 % removal efficiency of FA within 240 min		
$[Cu_2(CPBPY)_4(H_2O)_2][PW^{VI}_{12}O_{40}][OH]\cdot 6H_2O$; CPBY = *N*-(3-carboxyphenyl)-4,4'-bipyridinium	Keggin	MB	[MB] = 10 ppm; amount of catalyst = 50 mg; pH = 6.3; light source = 300 W Xe lamp; MB = methylene blue	98.2 % within 60 min under visible light and 97.7 % within 60 min under NIR light	nsp	[179]
$(NH_4)_5[\{PW^{VI}_{11}O_{39}\}Mn^{II}(H_2O)]$ (**1**) $(NH_4)_5[\{PW^{VI}_{11}O_{39}\}Fe^{II}(H_2O)]$ (**2**) $(NH_4)_5[\{PW^{VI}_{11}O_{39}\}Co^{II}(H_2O)]$ (**3**) $(NH_4)_3[PW^{VI}_{12}O_{40}]$ (**4**) $(NH_4)_5[\{PW^{VI}_{11}O_{39}\}Ni^{II}(H_2O)]$ (**5**) $(NH_4)_5[\{PW^{VI}_{11}O_{39}\}Cu^{II}(H_2O)]$ (**6**) $(NH_4)_5[\{PW^{VI}_{11}O_{39}\}Zn(H_2O)]$ (**7**)	Lacunary Keggin	MG	[MG] = 10 μM; amount of catalyst = 24 mg; pH = 5.77; light source = 500 W Xe lamp; in the presence of O_2; MG = malachite green	< 80 % removal efficiency of MG for (**1**), (**2**), (**3**), (**4**); > 80 % removal efficiency of MG for (**5**), (**6**), (**7**) within 30 min	5	[180]
SiW_{12} (**1**), $SiW_{11}V$ (**2**), $SiW_{10}V_2$ (**3**), SiW_9V_3 (**4**) PW_{12} (**5**), $PW_{11}V$ (**6**), $PW_{10}V_2$ (**7**), PW_9V_3 (**8**); $SiW_{12} = [\alpha\text{-}SiW^{VI}_{12}O_{40}]^{4-}$, $SiW_{11}V = [\alpha\text{-}SiV^{V}W^{VI}_{11}O_{40}]^{5-}$, $SiW_{10}V_2 = [\alpha\text{-}SiV^{V}_2W^{VI}_{10}O_{40}]^{6-}$, $SiW_9V_3 = [\alpha\text{-}SiV^{V}_3W^{VI}_9O_{40}]^{7-}$, $PW_{12} = [PW^{VI}_{12}O_{40}]^{4-}$, $PW_{11}V = [PV^{V}W^{VI}_{11}O_{40}]^{4-}$, $PW_{10}V_2 = [PV^{V}_2W^{VI}_{10}O_{40}]^{5-}$, $PW_9V_3 = [PV^{V}_3W^{VI}_9O_{40}]^{6-}$	Keggin	atrazine, chlorpyrifos, dieldrin	[pesticides] = 100 ppm; light source = 100 W Hg lamp	> 80 % removal efficiency of atrazine for (**1**); < 40 % removal efficiency of atrazine for (**4**) within 90 min; < 60 % removal efficiency of atrazine for (**5**); < 30 % removal efficiency of atrazine for (**8**) within 90 min; > 70 % removal efficiency of chlorpyrifos for (**1**); < 40 % removal efficiency of chlorpyrifos for (**4**) within 120 min; > 45 % removal efficiency of chlorpyrifos for (**5**); < 25 % removal efficiency of chlorpyrifos for (**8**) within 120 min; > 40 % removal efficiency of dieldrin for (**1**); < 30 % removal efficiency of dieldrin for (**4**) within 120 min; > 40 % removal efficiency of dieldrin for (**5**); < 20 % removal efficiency of Dieldrin for (**8**) within 120 min	nsp	[181]
$W^{VI}_{10}O_{32}{}^{4-}$	decatungstate	NAD	[NAD] = 3×10^{-4} M; amount of catalyst = 3×10^{-4} M; light source = 1000 W Xe lamp with a monochromator (λ = 365); NAD = 2-(1-naphthyl)acetamide	100 % within 22 h	nsp	[182]
APS-functionalized $TiO_2/CoPW^{VI}_{11}$ (**1**) APS-functionalized $TiO_2/NiPW^{VI}_{11}$ (**2**); APS = 3-aminopropyltriethoxysilane, $CoPW^{VI}_{11} = K_5[Co^{III}(H_2O)PW^{VI}_{11}O_{39}]$, $NiPW^{VI}_{11} = K_5[Ni^{II}(H_2O)PW^{VI}_{11}O_{39}]$	Keggin	CR, MO, AS, NR, HCB	[dye] = 50 ppm; [pesticide] = 2 ppm; amount of catalyst = 100 mg; light source = 125 W Hg lamp; CR = Congo red; MO = methyl orange; AS = alizarin S; NR = neutral red; HCB = hexachlorobenzene	94 % removal efficiency of CR for (**1**) and 93 % for (**2**) within 60 min; 98 % removal efficiency of MO for (**1**) and 95 % for (**2**) within 30 min; 94 % removal efficiency of NR for (**1**) and 90 % for (**2**) within 40 min; 89% removal efficiency of AS for (**1**) and 83 % for (**2**) within 240 min; 99.4 % removal efficiency of HCB for (**1**) and 97.8 % for (**2**) within	nsp	[183]

				60 min		
$[Fe(phen)_3]_2[SiW^{VI}_{12}O_{40}]\cdot 3$ DMF;<br>phen = 1,10-phenanthroline,<br>DMF = *N,N*-dimethylformamide	Keggin	DCP	[DCP] = 10 ppm; amount of catalyst = 300 ppm; light source = 400 W Hg lamp;<br>DCP = 2,4-dichlorophenol	100 % within 60 h	5	[184]
$(H_2bimb)[Cu^{II}(bimb)][SiW^{VI}_{12}O_{40}]\cdot 2H_2O$ (**1**)<br>$(H_2bimb)_2[Co^{II}(H_2O)_3(bimb)][SiW^{VI}_{11}Co^{II}O_{39}]\cdot 6H_2O$ (**2**)<br>$KH[Cu^{II}(bimb)]_2[SiW^{VI}_{11}Co^{II}O_{39}^{-??????}(H_2O)]\cdot 2H_2O$ (**3**)<br>$[Cu^{II}(bimb)]_4[GeW^{VI}_{12}O_{40}]\cdot H_2O$ (**4**);<br>bimb = 1,4-bis(1-imidazolyl)benzene;	Keggin	MB	[MB] = 10 ppm; amount of catalyst = 50 mg; light source = 125 W Hg lamp;<br>MB = methylene blue	89.5 % removal efficiency for (**1**);<br>87.5 % removal efficiency for (**2**);<br>90.4 % removal efficiency for (**3**);<br>84.7 % removal efficiency for (**4**) within 90 min	nsp	[185]
$H_3PW^{VI}_{12}O_{40}/SiO_2$ (**1**);<br>$H_4SiW^{VI}_{12}O_{40}/SiO_2$ (**2**)	Keggin	HCH, PCNB	[pesticides] = 6.5 ppm; amount of catalyst = 250 ppm for 1 and 350 ppm for 2; light source = 125 W Hg lamp;<br>HCH = hexachlorocyclohexane; PCNB = pentachloroe????nitrobenzene	95 % removal efficiency of HCH for (**1**) and 75 % for (**2**) within 4 h;<br>100 % removal efficiency of PCNB for (**2**) within 50 min	8	[186]
PW^{VI}_{12} (**1**)<br>SiW^{VI}_{12} (**2**)<br>GeW^{VI}_{12} (**3**);<br>$XW_{12} = [X^{n+}W^{VI}_{12}O_{40}]^{(8-n)-}$<br>($X^{n+} = P^{5+}, Si^{4+}, Ge^{4+}$)	Keggin	X-3B	[X-3B] = 6.37 × 10^{-5} M; amount of catalyst = 50 mg; pH = 1;<br>X-3B = reactive brilliant red	$k = 0.0004$ min^{-1} for (**1**);<br>$k = 0.0037$ min^{-1} for (**2**);<br>$k = 0.001$ min^{-1} for (**3**)	nsp	[187]
PW_{12}/TiO_2;<br>$PW_{12} = [PW^{VI}_{12}O_{40}]^{3-}$	Keggin	DEP, DMP, DBP	[dye] = 5 ppm; amount of catalyst = 100 mg; light source = 300 W Xe lamp;<br>DEP = diethyl phthalate; DMP = dimethyl phthalate; DBP = di-*n*-butyl phthalate	84 % removal efficiency of DEP;<br>80 % removal efficiency of DMP;<br>98 % removal efficiency of DBP within 90 min	nsp	[188]
$TEOS/PW_{12}$ (**1**)<br>$TEOS/SiW_{12}$ (**2**);<br>TEOS = tetraethoxysilane,<br>$PW_{12} = [PW^{VI}_{12}O_{40}]^{3-}$,<br>$SiW_{12} = [SiW^{VI}_{12}O_{40}]^{4-}$	Keggin	MB, RhB	[dye] = 2.975 × 10^{-5} M; amount of catalyst = 75 mg; light source = 100 W Hg lamp;<br>MB = methylene blue; RhB = rhodamine B	100 % removal efficiency of MB for (**1**) within 5 min;<br>89 % removal efficiency of RhB for (**1**) and 89 % for (**2**) within 5 min	nsp	[189]
TiO_2/PW_{12};<br>$PW_{12} = [PW^{VI}_{12}O_{40}]^{3-}$	Keggin	MO	[MO] = 10 ppm; pH = 2; light source = 300 W Hg lamp;<br>MO = methyl orange	93.2 %	nsp	[190]
$[SiW^{VI}_{12}O_{40}]^{4-}/rGO$;<br>rGO = reduced graphene oxide	Keggin	MB, RhB	[dye] = 35 ppm; $NaBH_4$ = 0.05 M; amount of catalyst = 0.5 mL;<br>MB = methylene blue; RhB = rhodamine B	reduction reactions completed in 34 min for MB and in 81 min for RhB	-	[191]
phenyl/amine Janus silica/ $PW^{VI}_{12}O_{40}^{3-}$	Keggin	MO	[MO] = 50 ppm; v[H_2O_2] = 10 µL;<br>MO = methyl orange	99.2 % within 3 h	6	[192]
$H_3PW^{VI}_{12}O_{40}$/*N*-decyl-*N'*-carboxymethyl imidazolium bromide	Keggin	MO	[MO] = 1.2 mM; amount of catalyst = 3.0 mL; pH = 3-6.5;<br>MO = methyl orange	Highly efficient degradation of MO without light irradiation in the presence of H_2O_2	6–8	[193]
$(NH_4)_3PMo^{VI}_{12}O_{40}$	Keggin	MB	[MB] = 10^{-5} M; amount of catalyst = 125 mg; pH = 5;<br>MB = methylene blue	94.6 %	16	[194]
GO-$H_3PW^{VI}_{12}O_{40}$-triethylenetetramine;<br>GO = graphene oxide	Keggin	MB	[MB] = 25 ppm; amount of catalyst = 25 mg; light source = sunlight; MB = methylene blue	84 % within 150 min	5	[195]
POMOF/wood filter;<br>POM = $H_3PMo^{VI}_{12}O_{40}$,<br>MOF = UiO-66,<br>MOF = metal-organic framework	Keggin	MB, GV	[dye] = 8 ppm;<br>MB = methylene blue; GV = gentian violet	96.63 %;<br>97.41 %	3	[196]
$(Et_4N)_4[V^{V}Mo^{VI}_{12}O_{40}]\cdot MeCN$;<br>MeCN = CH_3CN	Keggin	AB10B, MB	[dye] = 5 ppm; amount of catalyst = 3 mg for AB10B and 2 mg for MB; H_2O_2 30 % (2.0 mmol);<br>AB10B = amido black 10B; MB = methylene blue	99 % removal efficiency within 45 min for AB10B;<br>99 % removal efficiency within 20 min for MB	3	[197]
$H_6P_2Mo^{VI}_{15}W^{VI}_3O_{62}$ @MIL-96(Al);<br>MIL-96(Al) = Al-based metal-organic framework	Wells-Dawson	MB	[MB] = 40 ppm; amount of catalyst = 10 mg; pH = 4;<br>MB = methylene blue	92.4 % removal within 5 min	5	[198]
SPT-T-G;<br>SPT = $Na_6[H_2W^{VI}_{12}O_{40}]$,	Polytungs	AO	[AO] = 15 ppm; amount of catalyst = 40 mg; pH = 5;	100 % within 110 min	nsp	[199]

T = $C_4H_6O_6$,<br>G = $C_5H_9NO_4$			AO = auramine-O			
$[Cu^{II}_2(ipbp)_2(H_2O)_2][Cu^{II}W^{VI}_{12}O_{40}]\cdot 3H_2O$ (**1**)<br>$[Co^{II}_2(H_2ipbp)_3(H_2O)_2(Co^{II}O_6)_2]$ $[SiW^{VI}_{12}O_{40}]\cdot H_2O$ (**2**);<br>H_2ipbp·Cl = 1-(3,5-dicarboxyphenyl)-4,4′-bipyridinium	Keggin	MB, RhB	[dye] = 0.02 mM/L; amount of catalyst = 25 mg; under visible light;<br>MB = methylene blue; RhB = rhodamine B	complete degradation within 40 min for MB and 180 min for RhB by **1**;<br>complete degradation within 50 min for MB and 60 min for RhB by **2**	4-6	[200]
$(Et_3NH)_5[PMo^{V}_6Mo^{VI}_6O_{40}(V^{IV}O)_2]$	Keggin	MB, CV, RhB	[dye] = 100 mg/L; amount of catalyst = 300, 140, and 80 mg for MB, CV, and RhB;<br>MB = methylene blue; CV = crystal violet; RhB = rhodamine B	99.7 %, 99.8 %, 99.7 % flocculation efficiency for MB, CV, and RhB within 5 min	5	[201]
$[SiW^{VI}_9V^{V}_3O_{40}]^{7-}$@MIL-101(Cr);<br>MIL-101(Cr) = Cr-based metal-organic framework	Keggin	MB, RhB	[dye] = 10 mg/L; amount of catalyst = 30 mg;<br>MB = methylene blue; RhB = rhodamine B	98 % removal efficiency within 12 min for MB and 18 min for RhB	3	[202]
$[PW^{VI}_{12}O_{40}]^{3-}$@ZIF67 (**1**)<br>$[PM^{VI}o_{12}O_{40}]^{3-}$@ZIF-67 (**2**)<br>ZIF-67 = Co-based metal-organic framework	Keggin	*E. coli*<br>*S. aureus*	[sample] = 1 mg/ml	84.6% antibacterial efficacy against *E. coli* and 98.8% against *S. aureus* for (**1**) and 69.2% antibacterial efficacy against *E. coli* and 97.8% against *S. aureus* for (**2**)	nsp	[203]
$[Co(L)(V^{V}_4O_{12})_{0.5}(H_2O)]\cdot 2H_2O$<br>L = N,N′-bis(3-methylpyridin-3-yl)-2,6-naphthalenediamide	$[V_4O_{12}]^{4-}$ cluster	phenol, 2-CP, *m*-cresol	[phenolic compounds] = 400 mg/L; amount of catalyst = 10 mg; under visible light; $n(H_2O_2)/(phenol)$ = 46; 2-CP = 2-chlorophenol	94%, 92.9%, 92.6% degradation efficiency for phenol, 2-chlorophenol and m-cresol within 140 min	4	[204]
FeTCPP-PW^{VI}_{12} (**1**)<br>FeTCPP-PMo^{VI}_{12} (**2**)<br>H_4TCPP = tetrakis(4-carboxyphenyl) porphyrin	Keggin	phenol	[phenol] = 400 mg/L; amount of catalyst = 20 mg; light source = 300 W Xe lamp; H_2O_2 = 3ml (30%)	100% degradation efficiency within 20 min for (**1**) and 10 min for (**2**)	5	[205]
Pd-PTA-MIL-100(Fe)<br>Pd = Palladium nanoparticle<br>PTA = PW^{VI}_{12}<br>MIL-100(Fe) = Iron-based metal-organic framework	Keggin	theophylline, IBP	[pollutant] = 20 mg/L; T = 30 °C; amount of catalyst = 5 mg; pH = 6; H_2O_2 = 40 µL; light source = 300 W Xe lamp; IBP = ibuprofen	99% degradation efficiency for theophylline and ~ 50% for ibuprofen within 90 min	4	[206]
PMV-TiO_2/Ag<br>PMV = $[PMo^{VI}_{10}V^{V}_2O_{40}]^{5-}$<br>Ag = Silver nanoparticle	Keggin	2,4-DFP<br>2,4-DCP<br>2,4-DBP<br>HFBPA<br>TC<br>Cr(VI)<br>MO	[DHP] = 10 ppm; [HFBPA] = 10 ppm; [TC] = 20 ppm; [Cr(VI)] = 80 ppm; [MO] = 20 ppm; T = 10 °C; amount of catalyst = 20 mg; light source = 300 W Xe lamp<br>DHP = dihalophenol; HFBPA = hexafluorobisphenol; TC = tetracycline; Cr(VI) = $K_2Cr_2O_7$; MO = methyl orange	100% degradation efficiency for 2,4-DFP within 120 min; 100% degradation efficiency for 2,4-DCP within 180 min; 100% degradation efficiency for 2,4-DBP within 210 min; 100% degradation efficiency for HFBPA within 210 min; 90% degradation efficiency for TC within 80 min; 100% reduction efficiency for Cr(VI) within 30 min; 100% degradation efficiency for MO within 30 min;	4	[207]

*nsp- not specified by authors

References

1 Te M, Fairbridge C, Ring Z. Oxidation reactivities of dibenzothiophenes in polyoxometalate/H_2O_2 and formic acid/H_2O_2 systems. *Appl Catal A Gen.* 2001;219(1):267–80. doi: 10.1016/S0926-860X(01)00699-8

2 Chi M, Zhu Z, Sun L, Su T, Liao W, Deng C, et al. Construction of biomimetic catalysis system coupling polyoxometalates with deep eutectic solvents for selective aerobic oxidation desulfurization. *Appl Catal B Environ.* 2019;259:118089. doi: 10.1016/j.apcatb.2019.118089

3 Bertleff B, Claußnitzer J, Korth W, Wasserscheid P, Jess A, Albert J. Extraction coupled oxidative desulfurization of fuels to sulfate and water-soluble sulfur compounds using polyoxometalate catalysts and molecular oxygen. *ACS Sustain Chem Eng.* 2017;5(5):4110–8. doi: 10.1021/acssuschemeng.7b00087

4 Gao Y, Gao R, Zhang G, Zheng Y, Zhao J. Oxidative desulfurization of model fuel in the presence of molecular oxygen over polyoxometalate based catalysts supported on carbon nanotubes. *Fuel.* 2018;224:261–70. doi: 10.1016/j.fuel.2018.03.034

5 Zhang M, Liu J, Li H, Wei Y, Fu Y, Liao W, et al. Tuning the electrophilicity of vanadium-substituted polyoxometalate based ionic liquids for high-efficiency aerobic oxidative desulfurization. *Appl Catal B Environ.* 2020;271:118936. doi: 10.1016/j.apcatb.2020.118936

6 Li Y, Zhang H, Jiang Y, Shi M, Bawa M, Wang X, et al. Assembly of metallophthalocyanine-polyoxometalate hybrid for

highly efficient desulfurization of organic and inorganic sulfur under aerobic conditions. *Fuel.* 2019;241:861–9. doi: 10.1016/j.fuel.2018.12.091

7 Ribeiro S, Granadeiro CM, Silva P, Almeida Paz FA, Fabrizi de Biani F, Cunha-Silva L, et al. An efficient oxidative desulfurization process using terbium-polyoxometalate@MIL-101(Cr). *Catal Sci Technol.* 2013;3(9):2404–14. doi: 10.1039/C3CY00287J

8 Xun S, Zheng D, Yin S, Qin Y, Zhang M, Jiang W, et al. TiO_2 microspheres supported polyoxometalate-based ionic liquids induced catalytic oxidative deep-desulfurization. *RSC Adv.* 2016;6(48):42402–12. doi: 10.1039/C6RA03895F

9 Yang H, Jiang B, Sun Y, Zhang L, Huang Z, Sun Z, et al. Heterogeneous oxidative desulfurization of diesel fuel catalyzed by mesoporous polyoxometallate-based polymeric hybrid. *J Hazard Mater.* 2017;333:63–72. doi: 10.1016/j.jhazmat.2017.03.017

10 Komintarachat C, Trakarnpruk W. Oxidative desulfurization using polyoxometalates. *Ind Eng Chem Res.* 2006;45(6):1853–6. doi: 10.1021/ie051199x

11 Xu J, Zhu Z, Su T, Liao W, Deng C, Hao D, et al. Green aerobic oxidative desulfurization of diesel by constructing an Fe-Anderson type polyoxometalate and benzene sulfonic acid-based deep eutectic solvent biomimetic cycle. *Chin J Catal.* 2020;41(5):868–76. doi: 10.1016/S1872-2067(19)63500-X

12 Liu H, Xu H, Hua M, Chen L, Wei Y, Wang C, et al. Extraction combined catalytic oxidation desulfurization of petcoke in ionic liquid under mild conditions. *Fuel.* 2020;260:116200. doi: 10.1016/j.fuel.2019.116200

13 He J, Guan L, Zhou Y, Shao P, Yao Y, Lu S, et al. One-pot preparation of mesoporous $K_xPMo_{12}O_{40}$ (x = 1, 2, 3, 4) materials for oxidative desulfurization: electrochemically-active surface area (ECSA) determines their activity. *React Chem Eng.* 2020;5(9):1776–82. doi: 10.1039/D0RE00213E

14 Gao Y, Lv Z, Gao R, Hu G, Zhao J. Dawson type polyoxometalate based-poly ionic liquid supported on different carbon materials for high-efficiency oxidative desulfurization with molecular oxygen as the oxidant. *New J Chem.* 2020;44(46):20358–66. doi: 10.1039/D0NJ04526H

15 Tang A, Gu C, Zhan G, Zhang Z, Zhu W, Dai C, et al. Phosphomolybdic ionic liquid supported hydroxyapatite for heterogeneous oxidative desulfurization of fuels. *J Nanopart Res.* 2020;22(9):250. doi: 10.1007/s11051-020-04961-0

16 Wang P, Jiang L, Zou X, Tan H, Zhang P, Li J, et al. Confining polyoxometalate clusters into porous aromatic framework materials for catalytic desulfurization of dibenzothiophene. *ACS Appl Mater Interfaces*. 2020;12(23):25910–9. doi: 10.1021/acsami.0c05392

17 Ye G, Hu L, Gu Y, Lancelot C, Rives A, Lamonier C, et al. Synthesis of polyoxometalate encapsulated in UiO-66(Zr) with hierarchical porosity and double active sites for oxidation desulfurization of fuel oil at room temperature. *J Mater Chem A.* 2020;8(37):19396–404. doi: 10.1039/D0TA04337K

18 Ribeiro SO, Almeida PL, Pires J, de Castro B, Balula SS. Polyoxometalate@periodic mesoporous organosilicas as active materials for oxidative desulfurization of diesels. *Microporous Mesoporous Mater.* 2020;302:110193. doi: 10.1016/j.micromeso.2020.110193

19 Yang H, Zhang Q, Zhang J, Yang L, Ma Z, Wang L, et al. Cellulose nanocrystal shelled with poly(ionic liquid)/polyoxometalate hybrid as efficient catalyst for aerobic oxidative desulfurization. *J Colloid Interface Sci*. 2019;554:572–9. doi: 10.1016/j.jcis.2019.07.036

20 Akbari A, Chamack M, Omidkhah M. Reverse microemulsion synthesis of polyoxometalate-based heterogeneous hybrid catalysts for oxidative desulfurization. *J Mater Sci.* 2020;55(15):6513–24. doi: 10.1007/s10853-020-04458

21 Rezvani MA, Mirsadri SA. Synthesis and characterization of new hybrid inorganic-organic polymer nanocomposite as efficient catalyst for oxidative desulfurization of real fuel. *Appl Organomet Chem.* 2020;34(5):e5585. doi: 10.1002/aoc.5585

22 Rezvani MA, Hadi M, Mirsadri SA. Synthesis of new nanocomposite based on nanoceramic and mono substituted polyoxometalate, $PMo_{11}Cd@MnFe_2O_4$, with superior catalytic activity for oxidative desulfurization of real fuel. *Appl Organomet Chem.* 2020;34(10):e5882. doi: 10.1002/aoc.5882

23 Eseva E, Akopyan A, Schepina A, Anisimov A, Maximov A. Deep aerobic oxidative desulfurization of model fuel by Anderson-type polyoxometalate catalysts. *Catal Commun.* 2021;149:106256. doi: 10.1016/j.catcom.2020.106256

24 Lu Y, Yue C, Liu B, Zhang M, Li Y, Yang W, et al. The encapsulation of POM clusters into MIL-101(Cr) at molecular level: $LaW_{10}O_{36}$@MIL-101(Cr), an efficient catalyst for oxidative desulfurization. *Microporous Mesoporous Mater*. 2021;311:110694. doi: 10.1016/j.micromeso.2020.110694

25 Abazari R, Esrafili L, Morsali A, Wu Y, Gao J. PMo_{12}@UiO-67 nanocomposite as a novel non-leaching catalyst with enhanced performance durability for sulfur removal from liquid fuels with exceptionally diluted oxidant. *Appl Catal B Environ.* 2021;283:119582. doi: 10.1016/j.apcatb.2020.119582

26 Rezvani MA, Khandan S. Synthesis and characterization of new sandwich-type polyoxometalate/nanoceramic nanocomposite, $Fe_2W_{18}Fe_4@FeTiO_3$, as a highly efficient heterogeneous nanocatalyst for desulfurization of fuel. *Solid State Sci.* 2019;98:106036. doi: 10.1016/j.solidstatesciences.2019.106036

27 Ghahramaninezhad M, Pakdel F, Niknam Shahrak M. Boosting oxidative desulfurization of model fuel by POM-grafting ZIF-8 as a novel and efficient catalyst. *Polyhedron.* 2019;170:364–72. doi: 10.1016/j.poly.2019.05.058

28 Banisharif F, Dehghani MR, Capel-Sanchez MC, Campos-Martin JM. Highly catalytic oxidative desulfurization and denitrogenation of diesel using anchored-silica-gel vanadium-substituted Dawson-type polyoxometalate. *Catal Today*. 2019;333:219–25. doi: 10.1016/j.cattod.2018.07.009

29 Gao H, Wu X, Sun D, Niu G, Guan J, Meng X, et al. Preparation of core-shell $PW_{12}@TiO_2$ microspheres and oxidative desulfurization performance. *Dalton Trans.* 2019;48(17):5749–55. doi: 10.1039/C9DT00203K

30 Rezvani MA, Khandan S, Sabahi N, Saeidian H. Deep oxidative desulfurization of gas oil based on sandwich-type polysilicotungstate supported β-cyclodextrin composite as an efficient heterogeneous catalyst. *Chin J Chem Eng*. 2019;27(10):2418–26. doi: 10.1016/j.cjche.2018.10.024

31 Abdelrahman AA, Betiha MA, Rabie AM, Ahmed HS, Elshahat MF. Removal of refractory organo-sulfur compounds using an efficient and recyclable {Mo132} nanoball supported graphene oxide. *J Mol Liq.* 2018;252:121–32. doi: 10.1016/j.molliq.2017.12.124

32 Shi M, Zhang D, Yu X, Li Y, Wang X, Yang W. Deep oxidative desulfurization catalyzed by $(NH_4)_5H_6PV_8Mo_4O_{40}$ using molecular oxygen as an oxidant. *Fuel Process Technol*. 2017;160:136–42. doi: 10.1016/j.fuproc.2017.02.038

33 Rafiee E, Mirnezami F. Keggin-structured polyoxometalate-based ionic liquid salts: thermoregulated catalysts for rapid oxidation of sulfur-based compounds using H_2O_2 and extractive oxidation desulfurization of sulfur-containing model oil. *J Mol Liq.* 2014;199:156–61. doi: 10.1016/j.molliq.2014.08.036

34 Zhang G, Wang R, Yu F, Zhao H. Clean fuel-oriented investigation of thiophene oxidation by hydrogen peroxide using polyoxometalate as catalyst. *Chem Pap.* 2009;63(5):617–9. doi: 10.2478/s11696-009-0063-1

35 Chi M, Su T, Sun L, Zhu Z, Liao W, Ren W, et al. Biomimetic oxygen activation and electron transfer mechanism for oxidative desulfurization. *Appl Catal B Environ.* 2020;275:119134. doi: 10.1016/j.apcatb.2020.119134

36 Wu J, Li Y, Jiang M, Huo Y, Wang X, Wang X. Achieving deep desulfurization with inverse-micellar polyoxometalates and oxygen. *RSC Adv.* 2021;11(16):9043−7. doi: 10.1039/D1RA00428J
37 Li J, Guo Y, Tan J, Hu B. Polyoxometalate dicationic ionic liquids as catalyst for extractive coupled catalytic oxidative desulfurization. *Catalysts*. 2021;11(3):356. doi: 10.3390/catal11030356
38 Rezvani MA, Aghmasheh M. Synthesis of t-B.PWFe/NiO nanocomposite as an efficient and heterogeneous green nanocatalyst for catalytic oxidative-extractive desulfurization of gasoline. *Environ Prog Sustain Energy.* 2021;40(4):e13616. doi: 10.1002/ep.13616
39 Liu Y, Chu J, Lian L, Chen X, An S, Hong L, et al. Ultrafast oxidative desulfurization of diesel fuels by mass transfer enhancement of polyoxometalate modified alumina catalysts. Energy *Fuels.* 2021;35(3):2110−20. doi: 10.1021/acs.energyfuels.0c03673
40 Rezvani MA, Hadi M, Rezvani H. Synthesis of new nanocomposite based on ceramic and heteropolymolybdate using leaf extract of Aloe vera as a high-performance nanocatalyst to desulfurization of real fuel. *Appl Organomet Chem*. 2021;35(5):e6176. doi: 10.1002/aoc.6176
41 Liu Y, Wang F, Lv Y, Yu S, Wang R, Jiao W. Three-dimensional graphene oxide covalently functionalized with Dawson-type polyoxotungstates for oxidative desulfurization of model fuels. *Ind Eng Chem Res.* 2021;60(1):114−27. doi: 10.1021/acs.iecr.0c04384
42 Gao Y, Cheng L, Gao R, Hu G, Zhao J. Deep desulfurization of fuels using supported ionic liquid-polyoxometalate hybrid as catalyst: a comparison of different types of ionic liquids. *J Hazard Mater.* 2021;401:123267. doi: 10.1016/j.jhazmat.2020.123267
43 Du ZY, Yu YZ, Hong YL, Li NF, Han YM, Cao JP, et al. Polyoxometalate-based metal-organic frameworks with unique high-nuclearity water clusters. *ACS Appl Mater Interfaces*. 2020;12(51):57174−81. doi: 10.1021/acsami.0c18970
44 Vedachalam S, Boahene P, Dalai AK. Oxidative desulfurization of heavy gas oil over a Ti-TUD-1-supported Keggin-type molybdenum heteropolyacids. *Energy Fuels.* 2020;34(12):15299−5312. doi: 10.1021/acs.energyfuels.0c01527
45 Liu Y, Zuo P, Wang F, Lv Y, Wang R, Jiao W. Extraction combined oxidation desulfurization of dibenzothiophene using polyoxometalate-supported magnetic chitosan microspheres. *J Taiwan Inst Chem Eng.* 2020;117:112−22. doi: 10.1016/j.jtice.2020.12.003
46 Rezvani MA, Khalafi N. Synthesis of a new nanocomposite based on sandwich-type silicotungstate and polyvinyl alcohol with superior catalytic activity for deep desulfurization of real/thiophenic mode fuel. *J Coord Chem.* 2020;73(24):3395−411. doi: 10.1080/00958972.2020.1855332
47 Wang L, Wang H, Wang Y. Research of desulfurization of dibenzothiophene with SO_3H-functionalized morpholine heteropolyacid ionic liquid catalyst. *J Mol Struct.* 2020;1220:128779. doi: 10.1016/j.molstruc.2020.128779
48 Fu J, Ma W, Guo Y, Li X, Wang H, Fu C, et al. The ultra-deep desulfurization of model oil using amphipathic Lindqvist-type polyoxometalate-based TiO_2 nanofibres as catalysts. *Catal Lett.* 2021;151(7):2027−37. doi: 10.1007/s10562-020-03432-4
49 Qi Z, Huang Z, Wang H, Li L, Ye C, Qiu T. In situ bridging encapsulation of a carboxyl-functionalized phosphotungstic acid ionic liquid in UiO-66: a remarkable catalyst for oxidative desulfurization. *Chem Eng Sci.* 2020;225:115818. doi: 10.1016/j.ces.2020.115818
50 Mirante F, de Castro B, Granadeiro CM, Balula SS. Solvent-free desulfurization system to produce low-sulfur diesel using hybrid monovacant Keggin-type catalyst. *Molecules*. 2020;25(21):4961. doi: 10.3390/molecules25214961
51 Xun S, Ti Q, Wu L, He M, Wang C, Chen L, et al. Few layer g-C3N4 dispersed quaternary phosphonium ionic liquid for highly efficient catalytic oxidative desulfurization of fuel. *Energy Fuels*. 2020;34(10):12379−87. doi: 10.1021/acs.energyfuels.0c02357
52 Du Y, Zhou L, Liu Z, Yang L. Polyoxometalate-based 3DOM ZrO_2 material for deep oxidative desulfurization of DBT with ultrahigh stability. *J Porous Mater.* 2021;28(1):109−16. doi: 10.1007/s10934-020-00969-y
53 Liu H, Li Z, Dong J, Liu D, Liu C, Chi Y, et al. Polyoxometalates encapsulated into hollow double-shelled nanospheres as amphiphilic nanoreactors for an effective oxidative desulfurization. *Nanoscale.* 2020;12(31):16586−95. doi: 10.1039/D0NR03951A
54 Ma Z, Zhang J, Zhan H, Xu M, Yang H, Yang L, et al. Immobilization of monodisperse metal-oxo-cluster on graphene for aerobic oxidative desulfurization of fuel. *Process Saf???? Environ Prot.* 2020;140:26−33. doi: 10.1016/j.psep.2020.04.041
55 Rezvani MA, Aghmasheh M. Synthesis of a nanocomposite based on chitosan and modified heteropolyanion as a nanocatalyst for oxidative desulfurization of real and thiophenic model fuels. *J Coord Chem*. 2020;73(9):1407−24. doi: 10.1080/00958972.2020.1789916
56 Li Y, Song J, Jiang M, Bawa M, Wang X, Tian Y, et al. The fabrication of IMo_6@iPAF-1 as an enzyme mimic in heterogeneous catalysis for oxidative desulfurization under O_2 or air. *J Mater Chem A*. 2020;8(19):9813−24. doi: 10.1039/C9TA14066B
57 Li J, Yang Z, Hu G, Zhao J. Heteropolyacid supported MOF fibers for oxidative desulfurization of fuel. *Chem Eng J*. 2020;388:124325. doi: 10.1016/j.cej.2020.124325
58 Li SW, Wang W, Zhao JS. Effective and reusable oxidative desulfurization of dibenzothiophene via magnetic amino-MIL-101 supported $H_3PMo_6W_6O_{40}$ components: comparison influence on various types of MIL-101. *Energy Fuels*. 2020;34(4):4837−48. doi: 10.1021/acs.energyfuels.9b03996
59 Rezvani MA, Khalafi N. Deep oxidative desulfurization of real fuel and thiophenic model fuels using polyoxometalate-based catalytic nanohybrid material. *Mater Today Commun.* 2020;22:100730. doi: 10.1016/j.mtcomm.2019.100730
60 Gan M, Yang G, Wang Z, Sui X, Hou Y. Highly efficient oxidative desulfurization catalyzed by a polyoxometalate/carbonized cellulose nanofiber composite. Energy *Fuels.* 2020;34(1):778−86. doi: 10.1021/acs.energyfuels.9b03712
61 Yue D, Lei J, Zhou L, Du X, Guo Z, Li J. Oxidative desulfurization of fuels at room temperature using ordered meso/macroporous $H_3PW_{12}O_{40}/SiO_2$ catalyst with high specific surface areas. *Arab J Chem.* 2020;13(1):2649−58. doi: 10.1016/j.arabjc.2018.06.017
62 Mirante F, Alves AC, Julião D, Almeida PL, Gago S, Valença R, et al. Large-pore silica spheres as support for samarium-coordinated undecamolybdophosphate: oxidative desulfurization of diesels. *Fuel.* 2020;259:116213. doi: 10.1016/j.fuel.2019.116213
63 Rezvani MA, Rahmani P. Synthesis and characterization of new nanosphere hybrid nanocomposite polyoxometalate@ceramic@polyaniline as a heterogeneous catalyst for oxidative desulfurization of real fuel. *Adv Powder Technol.* 2019;30(12):3214−23. doi: 10.1016/j.apt.2019.09.030
64 Ribeiro SO, Granadeiro CM, Corvo MC, Pires J, Campos-Martin JM, de Castro B, et al. Mesoporous silica vs. organosilica composites to desulfurize diesel. *Front Chem.* 2019;7:756. doi: 10.3389/fchem.2019.00756
65 Rezvani MA, Shaterian M, Aghbolagh ZS, Akbarzadeh F. Synthesis and characterization of new inorganic-organic hybrid nanocomposite $PMo_{11}Cu@MgCu_2O_4$@CS as an efficient heterogeneous nanocatalyst for ODS of real fuel. *Chem Select.* 2019;4(20):6370−6. doi: 10.1002/slct.201900202
66 Ribeiro SO, Granadeiro CM, Almeida PL, Pires J, Valença R, Campos-Martin JM, et al. Effective zinc-substituted Keggin composite to catalyze the removal of sulfur from real diesels under

a solvent-free system. *Ind Eng Chem Res.* 2019;58(40):18540–9. doi: 10.1021/acs.iecr.9b02340
67 Mirante F, Gomes N, Corvo MC, Gago S, Balula SS. Polyoxomolybdate based ionic-liquids as active catalysts for oxidative desulfurization of simulated diesel. *Polyhedron.* 2019;170:762–70. doi: 10.1016/j.poly.2019.06.019
68 Rezvani MA, Oghoulbeyk ZN. Synthesis and characterization of new organic-inorganic nanohybrid film (TBA)PWFe/PVA/CTS as an efficient and reusable amphiphilic catalyst for ODS of real fuel. *Appl Organomet Chem.* 2019;33(12):e5241. doi: 10.1002/aoc.5241
69 Yu MY, Yang J, Xu X, Ma JF, Wang Z. Highly stable polyoxometalate-resorcinarene-based inorganic-organic complexes for catalytic oxidation desulfurization. *Appl Organomet Chem.* 2019;33(10):e5169. doi: 10.1002/aoc.5169
70 Yan XM, Mei P, Xiong L, Gao L, Yang Q, Gong L. Mesoporous titania-silica-polyoxometalate nanocomposite materials for catalytic oxidation desulfurization of fuel oil. *Catal Sci Technol.* 2013;3:1985–92. doi: 10.1039/C3CY20732C
71 Ribeiro SO, Granadeiro CM, Almeida PL, Pires J, Capel-Sanchez MC, Campos-Martin JM, et al. Oxidative desulfurization strategies using Keggin-type polyoxometalate catalysts: biphasic versus solvent-free systems. *Catal Today.* 2019;333:226–36. doi: 10.1016/j.cattod.2018.10.046
72 Sun L, Su T, Li P, Xu J, Chen N, Liao W, et al. Extraction coupled with aerobic oxidative desulfurization of model diesel using a B-type Anderson polyoxometalate catalyst in ionic liquids. *Catal Lett.* 2019;149(7):1888–93. doi: 10.1007/s10562-019-02791-x
73 Dou SY, Wang R. Ultradeep desulfurization of model oil through the oxidative adsorption process using Dawson-type polyoxometalates and graphene oxide multifunctional composites. *Appl Organomet Chem.* 2019;33(6):e4924. doi: 10.1002/aoc.4924
74 Sun L, Su T, Xu J, Hao D, Liao W, Zhao Y, et al. Aerobic oxidative desulfurization coupling of Co polyanion catalysts and p-TsOH-based deep eutectic solvents through a biomimetic approach. *Green Chem.* 2019;21(10):2629–34. doi: 10.1039/C8GC03941K
75 Ding J, Zhang Y, Wang R. Homogeneous oxidative desulfurization catalyzed by a recoverable reaction-controlled phase transfer catalyst based on trilacunary Keggin polyoxometalate. *New J Chem.* 2019;43(19):7363–70. doi: 10.1039/C9NJ00002J
76 Rezvani MA, Maleki Z. Facile synthesis of inorganic-organic $Fe_2W_{18}Fe_4$@NiO@CTS hybrid nanocatalyst induced efficient performance in oxidative desulfurization of real fuel. *Appl Organomet Chem.* 2019;33(5):e4895. doi: 10.1002/aoc.4895
77 Hao L, Sun L, Su T, Hao D, Liao W, Deng C, et al. Polyoxometalate-based ionic liquid catalyst with unprecedented activity and selectivity for oxidative desulfurization of diesel in [Omim]BF4. *Chem Eng J.* 2019;358:419–26. doi: 10.1016/j.cej.2018.10.006
78 Zhang L, Song S, Yang N, Tantai X, Xiao X, Jiang B, et al. Porous hybrid nanoflower self-assembled from polyoxometalate and polyionene for efficient oxidative desulfurization. *Ind Eng Chem Res.* 2019;58(9):3618–29. doi: 10.1021/acs.iecr.8b05905
79 Yue D, Lei J, Lina Z, Zhenran G, Du X, Li J. Three-dimensional ordered macroporous HPW/titania-alumina catalysts for catalytic oxidative desulfurization of fuels. *J Porous Mater.* 2019;26(1):133–44. doi: 10.1007/s10934-018-0627-y
80 Mirante F, Gomes N, Branco LC, Cunha-Silva L, Almeida PL, Pillinger M, et al. Mesoporous nanosilica-supported polyoxomolybdate as catalysts for sustainable desulfurization. *Microporous Mesoporous Mater.* 2019;275:163–71. doi: 10.1016/j.micromeso.2018.07.036
81 Dizaji AK, Mokhtarani B, Mortaheb HR. Deep and fast oxidative desulfurization of fuels using graphene oxide-based phosphotungstic acid catalysts. *Fuel.* 2019;236:717–29. doi: 10.1016/j.fuel.2018.09.076
82 Rezvani MA, Aghbolagh ZS, Monfared HH. Green and efficient organic-inorganic hybrid nanocatalyst for oxidative desulfurization of gasoline. *Appl Organomet Chem.* 2018;32(12):e4592. doi: 10.1002/aoc.4592
83 Jiang W, Jia H, Zheng Z, Zhu L, Dong L, Liu W, et al. Catalytic oxidative desulfurization of fuels in acidic deep eutectic solvents with $[(C_6H_{13})_3P(C_{14}H_{29})]_3PMo_{12}O_{40}$ as a catalyst. *Pet Sci.* 2018;15(4):841–8. doi: 10.1007/s12182-018-0263-9
84 Khodadadi Dizaji A, Mortaheb HR, Mokhtarani B. Extractive-catalytic oxidative desulfurization with graphene oxide-based heteropolyacid catalysts: investigation of affective parameters and kinetic modeling. *Catal Lett.* 2019;149(1):259–71. doi: 10.1007/s10562-018-2595-x
85 Zhang M, Wang M, Yang J, Li H, Liu J, Chen X, et al. Polyoxometalate-based silica-supported ionic liquids for heterogeneous oxidative desulfurization in fuels. *Pet Sci.* 2018;15(4):882–9. doi: 10.1007/s12182-018-0267-5
86 Rezvani MA, Khandan S. Synthesis and characterization of a new nanocomposite ($FeW_{11}V$@CTAB-MMT) as an efficient heterogeneous catalyst for oxidative desulfurization of gasoline. *Appl Organomet Chem.* 2018;32(11):e4524. doi: 10.1002/aoc.4524
87 Wang C, Chen Z, Yao X, Chao Y, Xun S, Xiong J, et al. Decavanadates anchored into micropores of graphene-like boron nitride: efficient heterogeneous catalysts for aerobic oxidative desulfurization. *Fuel.* 2018;230:104–12. doi: 10.1016/j.fuel.2018.04.153
88 Lin ZJ, Zheng HQ, Chen J, Zhuang WE, Lin YX, Su JW, et al. Encapsulation of phosphotungstic acid into metal-organic frameworks with tunable window sizes: screening of PTA@MOF catalysts for efficient oxidative desulfurization. *Inorg Chem.* 2018;57(20):13009–19. doi: 10.1021/acs.inorgchem.8b02272
89 Gao Y, Lv Z, Gao R, Zhang G, Zheng Y, Zhao J. Oxidative desulfurization process of model fuel under molecular oxygen by polyoxometalate loaded in hybrid material CNTs@MOF-199 as catalyst. *J Hazard Mater.* 2018;359:258–65. doi: 10.1016/j.jhazmat.2018.07.008
90 Craven M, Xiao D, Kunstmann-Olsen C, Kozhevnikova EF, Blanc F, Steiner A, et al. Oxidative desulfurization of diesel fuel catalyzed by polyoxometalate immobilized on phosphazene-functionalized silica. *Appl Catal B Environ.* 2018;231:82–91. doi: 10.1016/j.apcatb.2018.03.005
91 Bertleff B, Claußnitzer J, Korth W, Wasserscheid P, Jess A, Albert J. Catalyst activation and influence of the oil matrix on extractive oxidative desulfurization using aqueous polyoxometalate solutions and molecular oxygen. *Energy Fuels.* 2018;32(8):8683–8. doi: 10.1021/acs.energyfuels.8b01514
92 Yue S, Song Q, Zang S, Deng G, Li J. Synthesis of polyoxomolybdate-quinoline compounds beads for catalytic oxidative desulfurization. *Mol Catal.* 2018;455:88–94. doi: 10.1016/j.mcat.2018.02.014
93 Granadeiro CM, Ferreira PMC, Julião D, Ribeiro LA, Valença R, Ribeiro JC, et al. Efficient oxidative desulfurization processes using polyoxomolybdate based catalysts. *Energies.* 2018;11(7):1696. doi: 10.3390/en11071696
94 Li SW, Gao RM, Zhang W, Zhang Y, Zhao J. Heteropolyacids supported on macroporous materials POM@MOF-199@LZSM-5: highly catalytic performance in oxidative desulfurization of fuel oil with oxygen. *Fuel.* 2018;221:1–11. doi: 10.1016/j.fuel.2017.12.093
95 Yang H, Jiang B, Sun Y, Tantai X, Xiao X, Wang J, et al. Construction of polyoxomettallate-based organic-inorganic hybrid nanowires for efficient oxidative desulfurization. *Mol Catal.* 2018;448:38–45. doi: 10.1016/j.mcat.2018.01.016
96 Khodadadi Dizaji A, Mortaheb HR, Mokhtarani B. Complete oxidative desulfurization using graphene oxide-based phosphomolybdic acid catalyst: process optimization by two

phase mass balance approach. *Chem Eng J.* 2018;335:362-72. doi: 10.1016/j.cej.2017.10.129
97 Mirante F, Dias L, Silva M, Ribeiro SO, Corvo MC, de Castro B, et al. Efficient heterogeneous polyoxometalate-hybrid catalysts for the oxidative desulfurization of fuels. *Catal Commun.* 2018;104:1-8. doi: 10.1016/j.catcom.2017.10.006
98 Rafiee E, Joshaghani M, Ghaderi-Shekhi AP. Oxidative desulfurization of diesel by potato based-carbon as green support for H5PMo10V2O40: efficient composite nanorod catalyst. *J Saudi Chem Soc.* 2017;21(5):599–609. doi: 10.1016/j.jscs.2017.02.003
99 Zhang Y, Gu Y, Dong X, Wu P, Li Y, Hu H, et al. Deep oxidative desulfurization of refractory sulfur compounds with cesium salts of mono-substituted phosphomolybdate as efficient catalyst. *Catal Lett.* 2017;147(7):1811–9. doi: 10.1007/s10562-017-2078-5
100 Julião D, Valença R, Ribeiro JC, de Castro B, Balula SS. Efficient eco-sustainable ionic liquid-polyoxometalate desulfurization processes for model and real diesel. *Appl Catal A Gen.* 2017;537:93–9. doi: 10.1016/j.apcata.2017.02.021
101 Banisharif F, Dehghani MR, Campos-Martin JM. Oxidative desulfurization of diesel using vanadium-substituted Dawson-type emulsion catalysts. *Energy Fuels.* 2017;31(5):5419–27. doi: 10.1021/acs.energyfuels.6b02791
102 Rezvani MA, Khandan S, Sabahi N. Oxidative desulfurization of gas oil catalyzed by $(TBA)_4PW_{11}Fe$@PbO as an efficient and recoverable heterogeneous phase-transfer nanocatalyst. *Energy Fuels.* 2017;31(5):5472–81. doi: 10.1021/acs.energyfuels.7b00948
103 Ma W, Xu Y, Ma K, Luo Y, Liu Y, Zhang H. Synthesis of $PW_{11}Sn/TiO_2$ nanofibre catalytic materials with tunable rutile/anatase phase and application in ultra-deep desulfurization. *Mol Catal.* 2017;433:28–36. doi: 10.1016/j.mcat.2016.12.024
104 Jiang W, Zheng D, Xun S, Qin Y, Lu Q, Zhu W, et al. Polyoxometalate-based ionic liquid supported on graphite carbon induced solvent-free ultra-deep oxidative desulfurization of model fuels. *Fuel.* 2017;190:1–9. doi: 10.1016/j.fuel.2016.11.024
105 Choi AES, Roces S, Dugos N, Wan MW. Oxidation by H_2O_2 of benzothiophene and dibenzothiophene over different polyoxometalate catalysts in the frame of ultrasound and mixing assisted oxidative desulfurization. *Fuel.* 2016;180:127–36. doi: 10.1016/j.fuel.2016.04.014
106 Li ZZ, Wu JQ, Xue LL, Dong ZQ, Li SW, Wang W, et al. Ultra-deep and fast oxidative desulfurization by IL-POM immobilized on bar-shaped green fiber toward the high efficiency. *Appl Organomet Chem.* 2024;38(9):e7610. doi: 10.1002/aoc.7610
107 Zhang J, Wang F, Zhang X, Zhai Y, Wang K, Bing C, et al. In situ synthesis of hierarchical POM@HUSY with tunable Lewis and Brønsted acid sites for deep oxidative desulfurization of dibenzothiophene. *Ind Eng Chem Res.* 2024;63(25):10914–26. doi: 10.1021/acs.iecr.4c00279
108 Li Z, Huang L, Yu X, Huang K, Cui W, Zhang H. Iso-polyoxometalate $[Mo_8O_{26}]$4-(Mo_8) supported with mesoporous hexagonal boron nitride (h-BN): a green and efficient composite catalyst for ultradeep desulfurization of model fuel at room temperature. Energy *Fuels.* 2024;38(14):13218–27. doi: 10.1021/acs.energyfuels.4c00891
109 Gooneh-Farahani S, Anbia M. Investigating the catalytic performance of polyoxometalate immobilized on magnetic chitosan in the oxidative desulfurization process. *J Ind Eng Chem.* 2025;141:477–88. doi: 10.1016/j.jiec.2024.07.009
110 Rezvani MA, Hemmatzadeh A, Oroumi G, Feghh miri O. Enhanced catalytic activity of $PMo_{11}V$/NiO/PAN nanosphere composite for real fuel oxidation desulfurization: synthesis and characterization. *Mater Chem Phys.* 2024;311:128505. doi: 10.1016/j.matchemphys.2023.128504
111 Chu F, Wei X, Lu B, Zhao G, Li Y, Yang K, et al. Flexible encapsulation polyoxometalate with electrostatically interaction into metal-organic framework for ultra-deep aerobic oxidation desulfurization of diesel via a biomimetic approach in deep eutectic solvents. *Chem Eng J.* 2024;481:148549. doi: 10.1016/j.cej.2024.148549
112 Li J, Li H, Song Z, Guo Y, Tai M, Han M, et al. Catalytic oxidative desulfurization performance of a modified nano-sized β zeolite loaded with different structural polyoxometalates. *New J Chem.* 2024;48:8743–52. doi: 10.1039/D4NJ00214H
113 Wu JQ, Ma Ll, Li ZZ, Li X, Li SW, Li C, et al. Construction of large-aperture mesoporous silica spheres supported polyoxometalate heterogeneous catalysts and their high-efficiency for the ultra-deep desulfurization. *Fuel.* 2024;371(Pt A):131902. doi: 10.1016/j.fuel.2024.131902
114 Liao M, Xiao Y, Pi X, Zhang L, Zheng M, Liu J, et al. Zr-based metal-organic framework (UiO-66) doped with rare-earth element (La, Y)-supported polyoxometalate liquid for oxidative desulfurization of dibenzothiophene oxidation. *Energy Fuels*. 2024;38(14):12555–68. doi: 10.1021/acs.energyfuels.4c01125
115 Ma W, Ma Y, Kong L, Yang L, Wang R, Li D, et al. PW_{12}-MIL-101(Cr)/TiO_2 nanofibers: electrospinning synthesis and deep desulfurization performance under mild condition. *Energy Fuels.* 2024;38(7):6325–34. doi: 10.1021/acs.energyfuels.4c00249
116 Zhang J, Wu H, Yesire Y, Zhang Y, Ding J, Fan Y, et al. Amphiphilic catalysts comprising phosphomolybdic acid fastened on MIL-101(Cr): enabling efficient oxidative desulfurization under solvent-free and moderate reaction conditions. *Energy Fuels.* 2024;38(10):8553–63. doi: 10.1021/acs.energyfuels.4c00459
117 Liu F, Huang T, Chen M, He J, Li H, Wang C, et al. Facile preparation of decavanadate-based poly (ionic liquids) for efficient aerobic oxidative desulfurization. *J Mol Struct.* 2025;1324:140768. doi: 10.1016/j.molstruc.2024.140768
118 Afshari P, Khoshnavazi R, Rezvani MA. Synthesis and characterization of new nanocomposite $P_2W_{18}Co_4$@$ZnFe_2O_4$@PVA as an efficient and excellent magnetically recoverable nanocatalyst for desulfurization of fuels. *Mater Sci Eng B.* 2025;321:118512. doi: 10.1016/j.mseb.2025.118512
119 Ming P, Liu Y, Yu L, Li X, Liu R, Wang R. Development of magnetical Wells-Dawson polyoxometalates with defects for the highly effective oxidative desulfurization of fuel oil. *Sep Purif Technol.* 2025;360(1):130851. doi: 10.1016/j.seppur.2024.130851
120 Mao SX, Huang SY, Feng T, Pang JY, Dang DB, Bai Y. Enhanced oxidative desulfurization using carboxylic functionalized poly(ionic liquid)/polyoxomolybdates with double terminal oxygen active sites. *Sep Purif Technol.* 2025;362(1):131617. doi: 10.1016/j.seppur.2025.131617
121 Zhang K, Wang R. High-efficiency utilization of phase transfer catalysts based on Keggin polyoxometalate in oxidative desulfurization. *Sep Purif Technol.* 2025;371:133398. doi: 10.1016/j.seppur.2025.133398
122 Hao XL, Zhang CH, Feng CK, Su TR, Zhao Mm, Wen YZ. Two Dawson-type polyoxometalate-based metal coordination polymers as heterogeneous catalysts for desulfurization. *New J Chem.* 2025:Advance Article. doi: 10.1039/D5NJ02349A
123 Herrmann S, De Matteis L, de la Fuente JM, Mitchell SG, Streb C. Removal of multiple contaminants from water by polyoxometalate supported ionic liquid phases (POM-SILPs). *Angew Chem Int Ed.* 2017;56(6):1667–70. doi: 10.1002/anie.201611072
124 Yao L, Zhang L, Wang R, Chou S, Dong Z. A new integrated approach for dye removal from wastewater by polyoxometalates functionalized membranes. *J Hazard Mater.* 2016;301:462–70. doi: 10.1016/j.jhazmat.2015.09.027
125 Zeng L, Xiao L, Long Y, Shi X. Trichloroacetic acid-modulated synthesis of polyoxometalate@UiO-66 for selective adsorption of cationic dyes. *J Colloid Interface Sci.* 2018;516:274–83. doi: 10.1016/j.jcis.2018.01.070
126 Zhu TT, Zhang ZM, Chen WL, Liu ZJ, Wang EB. Encapsulation of tungstophosphoric acid into harmless MIL-101(Fe) for effectively removing cationic dye from aqueous solution. *RSC Adv.* 2016;6(85):81622–30. doi: 10.1039/C6RA16716K

127 Chai DF, Wang M, Zhang C, Ning F, Xu W, Pang H, et al. A novel 3D POMOF based on dinuclear copper(II)-oxalate complexes and Keggin polyoxoanions with excellent photocatalytic activity. *Inorg Chem Commun.* 2017;83:16–9. doi: 10.1016/j.inoche.2017.05.028
128 Cong BW, Su ZH, Zhao ZF, Zhao WQ, Ma XJ, Xu Q, et al. A new 3D POMOF with two channels consisting of Wells-Dawson arsenotungstate and $\{Cl_4Cu_{10}(pz)_{11}\}$ complexes: synthesis, crystal structure, and properties. *New J Chem.* 2018;42(6):4596–602. doi: 10.1039/C7NJ04854H
129 Huo M, Yang W, Zhang H, Zhang L, Liao J, Lin L, et al. A new POM-MOF hybrid microporous material with ultrahigh thermal stability and selective adsorption of organic dyes. RSC Adv. 2016;6(112):111549–55. doi: 10.1039/C6RA10422C
130 Hoseini A, Farhadi S, Zabardasti A. Yolk-shell microspheres assembled from Preyssler-type NaP5W30O11014-polyoxometalate and MIL-101(Cr) metal-organic framework: a new inorganic-organic nanohybrid for fast and selective removal of cationic organic dyes from aqueous media. *Appl Organomet Chem.* 2019;33(2):e4656. doi: 10.1002/aoc.4656
131 Jarrah A, Farhadi S. Preparation and characterization of novel polyoxometalate/$CoFe_2O_4$/metal-organic framework magnetic core-shell nanocomposites for the rapid removal of organic dyes from water. *RSC Adv.* 2020;10(65):39881–93. doi: 10.1039/D0RA04603E
132 Liu X, Luo J, Zhu Y, Yang Y, Yang S. Removal of methylene blue from aqueous solutions by an adsorbent based on metal-organic framework and polyoxometalate. *J Alloys Compd.* 2015;648:986–93. doi: 10.1016/j.jallcom.2015.07.065
133 Sun JW, Yan PF, An GH, Sha JQ, Li GM, Yang GY. Immobilization of polyoxometalate in the metal-organic framework rht-MOF-1: towards a highly effective heterogeneous catalyst and dye scavenger. *Sci Rep*. 2016;6:25595. doi: 10.1038/srep25595
134 Liu X, Gong W, Luo J, Zou C, Yang Y, Yang S. Selective adsorption of cationic dyes from aqueous solution by polyoxometalate-based metal-organic framework composite. *Appl Surf Sci.* 2016;362:517–24. doi: 10.1016/j.apsusc.2015.11.151
135 Zhang L, Chen H, Zhao X, Zhai Q, Yin D, Sun Y, et al. The marriage of ferrocene and silicotungstate: an ingenious heterogeneous Fenton-like synergistic photocatalyst. *Appl Catal B Environ.* 2016;193:47–57. doi: 10.1016/j.apcatb.2016.04.019
136 Wang Q, Liu E, Zhang C, Huang S, Cong Y, Zhang Y. Synthesis of $Cs_3PMo_{12}O_{40}/Bi_2O_3$ composite with highly enhanced photocatalytic activity under visible-light irradiation. *J Colloid Interface Sci.* 2018;516:304–11. doi: 10.1016/j.jcis.2018.01.065
137 Li L, Sun JW, Sha JQ, Li GM, Yan PF, Wang C, et al. Structure refinement and photocatalytic properties of porous POMCPs by selecting the isomerous PYTTZ. *Dalton Trans.* 2015;44(4):1948–54. doi: 10.1039/C4DT02960G
138 Wang J, Chen Y, Cheng N, Feng L, Gu BH, Liu Y. Multivalent supramolecular self-assembly between β-cyclodextrin derivatives and polyoxometalate for photodegradation of dyes and antibiotics. *ACS Appl Bio Mater.* 2019;2(12):5898–904. doi: 10.1021/acsabm.9b00845
139 Antonaraki S, Triantis TM, Papaconstantinou E, Hiskia A. Photocatalytic degradation of lindane by polyoxometalates: intermediates and mechanistic aspects. *Catal Today.* 2010;151(1-2):119–24. doi: 10.1016/j.cattod.2010.02.017
140 Misra A, Zambrzycki C, Kloker G, Kotyrba A, Anjass MH, Franco Castillo I, et al. Water purification and microplastics removal using magnetic polyoxometalate-supported ionic liquid phases (MagPOM-SILPs). *Angew Chem Int Ed Engl*. 2020;59(4):1601-5. doi: 10.1002/anie.201912111
141 Cui ZW, Wang XL, Lin HY, Xu N, Wang X, Liu GC, et al. Two Anderson-type polyoxometalate-based metal-organic complexes with a flexible bis(pyrazine)-bis(amide) ligand for rapid adsorption and selective separation of cationic dyes. *Inorg Chim Acta.* 2020;513:119937. doi: 10.1016/j.ica.2020.119937
142 Sabarinathan C, Karuppasamy P, Vijayakumar C, Arumuganathan T. Development of methylene blue removal methodology by adsorption using molecular polyoxometalate: kinetics, thermodynamics and mechanistic study. *Microchem J.* 2019;146:315–26. doi: 10.1016/j.microc.2019.01.015
143 Yang H, Bai L, Wei D, Yang L, Wang W, Chen H, et al. Ionic self-assembly of poly(ionic liquid)-polyoxometalate hybrids for selective adsorption of anionic dyes. *Chem Eng J.* 2019;358:850–9. doi: 10.1016/j.cej.2018.10.100
144 Li JH, Wang XL, Song G, Lin HY, Wang X, Liu GC. Various Anderson-type polyoxometalate-based metal-organic complexes induced by diverse solvents: assembly, structures and selective adsorption for organic dyes. *Dalton Trans.* 2020;49(4):1265–75. doi: 10.1039/C9DT04397G
145 Liang S, Nie YM, Li SH, Zhou JL, Yan J. A comprehensive study on the dye adsorption behavior of polyoxometalate-complex nano-hybrids containing classic β-octamolybdate and biimidazole units. *Molecules.* 2019;24(4):806. doi: 10.3390/molecules24040806
146 Li J, Zhao H, Ma C, Han Q, Li M, Liu H. Preparation of Fe_3O_4@polyoxometalates nanocomposites and their efficient adsorption of cationic dyes from aqueous solution. *Nanomaterials.* 2019;9(4):649. doi: 10.3390/nano9040649
147 Zhang J, Miao Z, Yan J, Zhang X, Li X, Zhang Q, et al. Synthesis of negative-charged metal-containing cyclomatrix polyphosphazene microspheres based on polyoxometalates and application in charge-selective dye adsorption. *Macromol Rapid Commun.* 2019;40(17):1800730. doi: 10.1002/marc.201800730
148 Li X, Pillai SC, Wei L, Liu Z, Huang L, Huang Q, et al. Facile synthesis of polyoxometalate-modified metal organic frameworks for eliminating tetrabromobisphenol-A from water. *J Hazard Mater.* 2020;399:122946. doi: 10.1016/j.jhazmat.2020.122946
149 Pourzare K, Farhadi S, Mansourpanah Y. Anchoring $H_3PW_{12}O_{40}$ on aminopropylsilanized spinel-type cobalt oxide (Co_3O_4-$SiPrNH_2$/$H_3PW_{12}O_{40}$): a novel nanohybrid adsorbent for removing cationic organic dye pollutants from aqueous solutions. *Appl Organomet Chem.* 2018;32(5):e4341. doi: 10.1002/aoc.4341
150 Ou J, Mei M, Xu X. Magnetic adsorbent constructed from the loading of amino functionalized Fe_3O_4 on coordination complex modified polyoxometalates nanoparticle and its tetracycline adsorption removal property study. *J Solid State Chem.* 2016;238:182–8. doi: 10.1016/j.jssc.2016.03.021
151 Farhadi S, Mahmoudi F, Amini MM, Dusek M, Jarosova M. Synthesis and characterization of a series of novel perovskite-type $LaMnO_3$/Keggin-type polyoxometalate hybrid nanomaterials for fast and selective removal of cationic dyes from aqueous solutions. *Dalton Trans.* 2017;46(10):3252–64. doi: 10.1039/C6DT04866H
152 Farhadi S, Mahmoudi F. Improving the adsorption ability of perovskite-type $LaNiO_3$ nanomaterial towards organic dyes by hybridizing with phosphotungstic acid. *Polyhedron.* 2019;169:39–50. doi: 10.1016/j.poly.2019.05.008
153 Cheng X, Sun P, Zhang S, Sun D, Jiang B, Wang W, et al. Self-assembly of m-phenylenediamine and polyoxometalate into hollow-sphere and core-in-hollow-shell nanostructures for selective adsorption of dyes. *J Mol Liq.* 2019;287:110982. doi: 10.1016/j.molliq.2019.110982
154 Farhadi S, Amini MM, Dusek M, Kucerakova M, Mahmoudi F. A new nanohybrid material constructed from Keggin-type polyoxometalate and Cd(II) semicarbazone Schiff base complex with excellent adsorption properties for the removal of cationic dye pollutants. *J Mol Struct.* 2017;1130:592–602. doi: 10.1016/j.molstruc.2016.10.081
155 Siadatnasab F, Karami K, Khataee A. Keggin-type polyoxometalates supported on PANI-coated CuS: synthesis, characterization and application as the efficient adsorbents for

selective dye removal. *J Ind Eng Chem.* 2019;80:205–16. doi: 10.1016/j.jiec.2019.07.050
156 Qi L, Gong Y, Fang M, Jia Z, Cheng N, Yu L. Surface-active ionic-liquid-encapsulated polyoxometalate nanospheres: construction, self-assembly, adsorption behavior, and application for dye removal. *ACS Appl Nano Mater.* 2020;3(1):375–83. doi: 10.1021/acsanm.9b02012
157 Bajuk-Bogdanović D, Jović A, Nedić Vasiljević B, Milojević-Rakić M, Kragović M, Krajišnik D, et al. 12-Tungstophosphoric acid/BEA zeolite composites–characterization and application for pesticide removal. *Mater Sci Eng B.* 2017;225:60–7. doi: 10.1016/j.mseb.2017.08.011
158 Sen W, Xiao W, Xiao-yu S, Cai-xia M, Cheng-lin S, Zhong-Shuai W. A three-dimensional polyoxometalate/graphene aerogel as a highly efficient and recyclable absorbent for oil/water separation. *New Carbon Mater.* 2021;36(1):189–97. doi: 10.1016/S1872-5805(21)60013-6
159 Zhang HY, Liu L, Wang HJ, Sun JW. Asymmetrical modification of Keggin polyoxometalates by sextuple Ag-N coordination polymeric chains: synthesis, structure and selective separation of cationic dyes. *J Solid State Chem.* 2021;296:121986. doi: 10.1016/j.jssc.2021.121986
160 Palapa NR, Taher T, Mohadi R, Rachmat A, Mardiyanto M, Miksusanti M, et al. NiAl-layered double hydroxide intercalated with Keggin polyoxometalate as adsorbent of malachite green: kinetic and equilibrium studies. *Chem Eng Commun.* 2022;209(5):684–95. doi: 10.1080/00986445.2021.1895773
161 Yao L, Lua SK, Zhang L, Wang R, Dong Z. Dye removal by surfactant encapsulated polyoxometalates. *J Hazard Mater.* 2014;280:428–35. doi: 10.1016/j.jhazmat.2014.08.026
162 Liu Y, Luo F, Liu S, Liu S, Lai X, Li X, et al. Aminated graphene oxide impregnated with photocatalytic polyoxometalate for efficient adsorption of dye pollutants and its facile and complete photoregeneration. *Small.* 2017;13(14):1603174. doi: 10.1002/smll.201603174
163 Xu M, Bi B, Xu B, Sun Z, Xu L. Polyoxometalate-intercalated ZnAlFe-layered double hydroxides for adsorbing removal and photocatalytic degradation of cationic dye. *Appl Clay Sci.* 2018;157:86–91. doi: 10.1016/j.clay.2018.02.023
164 Zhang D, Li Y, Gao Y, Bawa M, Huo M, Wang X, et al. Fast degradation of phthalate acid esters by polyoxometalate nanocatalysts through adsorption, esterolysis and oxidation. *J Hazard Mater.* 2019;368:788–96. doi: 10.1016/j.jhazmat.2019.01.113
165 Wu X, Luo B, Chen M, Chen F. Tunable surface charge of Fe, Mn substituted polyoxometalates/hydrotalcites for efficient removal of multiple dyes. *Appl Surf Sci.* 2020;509:145344. doi: 10.1016/j.apsusc.2020.145344
166 Bastami TR, Ahmadpour A. Preparation of magnetic photocatalyst nanohybrid decorated by polyoxometalate for the degradation of a pharmaceutical pollutant under solar light. *Environ Sci Pollut Res.* 2016;23(9):8849–60. doi: 10.1007/s11356-015-5985-2
167 Cao M, Lin J, Lü J, You Y, Liu T, Cao R. Development of a polyoxometallate-based photocatalyst assembled with cucurbituril via hydrogen bonds for azo dyes degradation. *J Hazard Mater.* 2011;186(1):948–51. doi: 10.1016/j.jhazmat.2010.10.119
168 Ghalebi HR, Aber S, Karimi A. Keggin type of cesium phosphomolybdate synthesized via solid-state reaction as an efficient catalyst for the photodegradation of a dye pollutant in aqueous phase. *J Mol Catal A Chem.* 2016;415:96–103. doi: 10.1016/j.molcata.2016.01.031
169 Chen DM, Liu XH, Zhang NN, Liu CS, Du M. Immobilization of polyoxometalate in a cage-based metal-organic framework towards enhanced stability and highly effective dye degradation. *Polyhedron.* 2018;152:108–13. doi: 10.1016/j.poly.2018.05.059
170 Lei P, Chen C, Yang J, Ma W, Zhao J, Zang L. Degradation of dye pollutants by immobilized polyoxometalate with H2O2 under visible-light irradiation. *Environ Sci Technol.* 2005;39(21):8466–74. doi: 10.1021/es050321g
171 Chen C, Wang Q, Lei P, Song W, Ma W, Zhao J. Photodegradation of dye pollutants catalyzed by porous $K_3PW_{12}O_{40}$ under visible irradiation. *Environ Sci Technol.* 2006;40(12):3965–70. doi: 10.1021/es060146j
172 Niu J, Zhang S, Chen H, Zhao J, Ma P, Wang J. 1-D, 2-D, and 3-D organic-inorganic hybrids assembled from Keggin-type polyoxometalates and 3d-4f heterometals. *Cryst Growth Des.* 2011;11(9):3769–77. doi: 10.1021/cg2001249
173 Sampurnam S, Muthamizh S, Dhanasekaran T, Latha D, Padmanaban A, Selvam P, et al. Synthesis and characterization of Keggin-type polyoxometalate/zirconia nanocomposites: comparison of its photocatalytic activity towards various organic pollutants. *J Photochem Photobiol A Chem.* 2019;370:26–40. doi: 10.1016/j.jphotochem.2018.10.031
174 Hu Y, Luo F, Dong F. Design synthesis and photocatalytic activity of a novel lilac-like silver-vanadate hybrid solid based on dicyclic rings of $[V_4O_{12}]^{4-}$ with $\{Ag_7\}^{7+}$ cluster. *Chem Commun.* 2011;47(2):761–3. doi: 10.1039/C0CC02965C
175 Chen DM, Zhang XJ. A polyoxometalate template metal-organic framework with unusual $\{Cu_8(\mu_4\text{-}OH)_6\}$10+ secondary building unit for photocatalytic dye degradation. *Inorg Chem Commun.* 2019;108:107523. doi: 10.1016/j.inoche.2019.107523
176 Zhou X, Ji YX, Cao JF, Xin ZF. Polyoxometalate encapsulated in metal-organic gel as an efficient catalyst for visible-light-driven dye degradation applications. *Appl Organomet Chem.* 2018;32(3):e4206. doi: 10.1002/aoc.4206
177 Tang Q, An X, Lan H, Liu H, Qu J. Polyoxometalates/TiO_2 photocatalysts with engineered facets for enhanced degradation of bisphenol A through persulfate activation. *Appl Catal B Environ.* 2020;268:118394. doi: 10.1016/j.apcatb.2019.118394
178 Yang Y, Wu Q, Guo Y, Hu C, Wang E. Efficient degradation of dye pollutants on nanoporous polyoxotungstate-anatase composite under visible-light irradiation. *J Mol Catal A Chem.* 2005;225(2):203–12. doi: 10.1016/j.molcata.2004.08.031
179 Sun X, Zhang J, Fu Z. Polyoxometalate cluster sensitized with copper-viologen framework for efficient degradation of organic dye in ultraviolet, visible, and near-infrared light. *ACS Appl Mater Interfaces.* 2018;10(42):35671–5. doi: 10.1021/acsami.8b10777
180 Liu CG, Zheng T, Liu S, Zhang HY. Photodegradation of malachite green dye catalyzed by Keggin-type polyoxometalates under visible-light irradiation: transition metal substituted effects. *J Mol Struct.* 2016;1110:44–52. doi: 10.1016/j.molstruc.2016.01.015
181 Youssef L, El-Rassy H, Younes G, Al-Oweini R. Photocatalytic and kinetic study on the degradation of three food pesticides using vanadium-substituted polyoxotungstates. *Int J Environ Res.* 2019;13(6):899–907. doi: 10.1007/s41742-019-00226-4
182 Da Silva ES, Sarakha M, Burrows HD, Wong-Wah-Chung P. Decatungstate anion as an efficient photocatalytic species for the transformation of the pesticide 2-(1-naphthyl)acetamide in aqueous solution. *J Photochem Photobiol A Chem.* 2017;334:61–73. doi: 10.1016/j.jphotochem.2016.10.036
183 Yang Y, Guo Y, Hu C, Wang Y, Wang E. Preparation of surface modifications of mesoporous titania with monosubstituted Keggin units and their catalytic performance for organochlorine pesticide and dyes under UV irradiation. *Appl Catal A Gen.* 2004;273(1–2):201–10. doi: 10.1016/j.apcata.2004.06.032
184 Shahrnoy AA, Mahjoub AR, Morsali A, Dusek M, Eigner V. Sonochemical synthesis of polyoxometalate based ionic crystal nanostructure: a photocatalyst for degradation of 2,4-dichlorophenol. *Ultrason Sonochem.* 2018;40:174–83. doi: 10.1016/j.ultsonch.2017.07.018
185 Hao HF, Zhou WZ, Zang HY, Tan HQ, Qi YF, Wang YH, et al. Keggin-type polyoxometalate-based metal-organic networks for

photocatalytic dye degradation. *Chem Asian J.* 2015;10(8):1676−83. doi: 10.1002/asia.201500424
186 Guo Y, Wang Y, Hu C, Wang Y, Wang E, Zhou Y, et al. Microporous polyoxometalates POMs/SiO_2: synthesis and photocatalytic degradation of aqueous organochlorine pesticides. *Chem Mater.* 2000;12(11):3501−8. doi: 10.1021/cm000074+
187 Jin H, Wu Q, Pang W. Photocatalytic degradation of textile dye X-3B using polyoxometalate-TiO_2 hybrid materials. *J Hazard Mater.* 2007;141(1):123−7. doi: 10.1016/j.jhazmat.2006.06.098
188 Xu L, Yang X, Guo Y, Ma F, Guo Y, Yuan X, et al. Simulated sunlight photodegradation of aqueous phthalate esters catalyzed by the polyoxotungstate/titania nanocomposite. *J Hazard Mater.* 2010;178(1-3):1070−7. doi: 10.1016/j.jhazmat.2010.02.049
189 Yahya F, El-Rassy H, Younes G, Al-Oweini R. Synthesis and characterisation of mesoporous hybrid silica-polyoxometalate aerogels for photocatalytic degradation of rhodamine B and methylene blue. *Int J Environ Anal Chem.* 2019;99(14):1375−96. doi: 10.1080/03067319.2019.1622010
190 Niu P, Hao J. Fabrication of titanium dioxide and tungstophosphate nanocomposite films and their photocatalytic degradation for methyl orange. *Langmuir.* 2011;27(22):13590−7. doi: 10.1021/la203178s
191 Ucar A, Findik M, Gubbuk IH, Kocak N, Bingol H. Catalytic degradation of organic dye using reduced graphene oxide-polyoxometalate nanocomposite. *Mater Chem Phys.* 2017;196:21−8. doi: 10.1016/j.matchemphys.2017.04.047
192 Ji X, Zhang Q, Qu X, Wang Q, Song XM, Liang F, et al. Poly(ionic liquid) Janus nanosheets towards dye degradation. *RSC Adv.* 2015;5(28):21877−80. doi: 10.1039/C5RA02330K
193 Gong Y, Guo Y, Hu Q, Wang C, Zang L, Yu L. pH-responsive polyoxometalate-based supramolecular hybrid nanomaterials and application as renewable catalyst for dyes. *ACS Sustain Chem Eng.* 2017;5(5):3650−8. doi: 10.1021/acssuschemeng.6b02791
194 Joseph J, Radhakrishnan RC, Johnson JK, Joy SP, Thomas J. Ion-exchange mediated removal of cationic dye-stuffs from water using ammonium phosphomolybdate. *Mater Chem Phys.* 2020;242:122488. doi: 10.1016/j.matchemphys.2019.122488
195 Yu L, Liu Q, Ding S, Yu J, Peng S, Zhang J, et al. The assembly of polyoxometalate-graphene oxide composites for photocatalytic removal of organic dye in water. *Appl Surf Sci.* 2022;602:154095. doi: 10.1016/j.apsusc.2022.154095
196 Cui Z, Wu J, Xu Y, Wu T, Li H, Li J, et al. In-situ growth of polyoxometalate-based metal-organic frameworks on wood as a promising dual-function filter for effective hazardous dye and iodine capture. *Chem Eng J.* 2023;451:138371. doi: 10.1016/j.cej.2022.138371
197 Naslhajian H, Amini M, Hosseinifard M, Farnia SMF, Janczak J. Synthesis and characterization of a new polyoxometalate nanocluster containing Mo and V as an environmentally green catalyst for oxidative degradation of organic pollutants from aquatic environments. *Appl Organomet Chem.* 2021;36:e6511. doi: 10.1002/aoc.6511
198 Hu H, He YP, Zhang YL, Feng SY, Li XJ, Yang Y, et al. Solvothermal synthesis of polyoxometalate modified metal-organic framework for enhanced removal of methylene blue from aqueous solution. *Russ J Phys Chem A.* 2022;96(Suppl 1):S44−50. doi: 10.1134/S0036024422140138
199 Mousavi SM, Hashemi SA, Bahrani S, Mosleh S, Chiang WH, Yousefi K, et al. Hybrid of sodium polytungstate polyoxometalate supported by the green substrate for photocatalytic degradation of auramine-O dye. *Environ Sci Pollut Res.* 2022;29:56055−67. doi: 10.1007/s11356-022-19767-x
200 Huang K, Huang L, Shen Y, Hua Y, Song R, Li Z, et al. Two novel 3D polyoxometalate-based metal-organic frameworks for structure-directed selective adsorption and photodegradation of organic dyes. *Inorg Chem Commun.* 2024;170:113350. doi: 10.1016/j.inoche.2024.113350
201 Chen L, Cui HW, Kong LY, Long JY, Fei BL. Efficient removal of cationic dyes by a bivanadyl capped, highly reduced Keggin polyoxometalate through flocculation. *Colloids Surf A.* 2024;730:135399. doi: 10.1016/j.colsurfa.2024.135399
202 Malmir H, Zonoz FM, Baghayeri M, Tayebee R. Synthesis, characterization, and application of mixed-addenda silicon vanado tungstate polyoxometalate integrated into nanoporous MIL-101(Cr) for the quick removal of organic dyes from water. *RSC Adv.* 2025;15:8918−31. doi: 10.1039/D5RA00443H
203 Mohamed AM, Abbas WA, Khedr GE, Abass W, Allam NK. Computational and experimental elucidation of the boosted stability and antibacterial activity of ZIF-67 upon optimized encapsulation with polyoxometalates. *Sci Rep.* 2022;12:15989. doi: 10.1038/s41598-022-20392-4
204 Li S, Zheng Y, Liu GC, Li XH, Zhang Z, Wang XL. New two-fold interpenetrating 3D polyoxovanadate-based metal-organic framework as bifunctional catalyst for the removal of 2-chloroethyl ethyl sulfide and phenolic compounds. *Polyoxometalates.* 2024;3:9140061. doi: 10.26599/POM.2024.9140061
205 Xia Z, Wang L, Zhang Q, Li F, Xu L. Fast degradation of phenol over porphyrin-polyoxometalate composite photocatalysts under visible light. *Polyoxometalates.* 2022;1:9140001. doi: 10.26599/POM.2022.9140001
206 Liang R, Huang R, Ying S, Wang X, Yan G, Wu L. Facile in situ growth of highly dispersed palladium on phosphotungstic-acid-encapsulated MIL-100(Fe) for the degradation of pharmaceuticals and personal care products under visible light. *Nano Res.* 2018;11:1109−23. doi: 10.1007/s12274-017-1730-0
207 Zhao XY, Wang X, Zhao Y, Sun H, Tan H, Qiu T, et al. Polyoxometalates-doped TiO_2/Ag hybrid heterojunction: removal of multiple pollutants and mechanism investigation. *Environ Sci Nano.* 2021;8:3855−64. doi: 10.1039/D1EN00827G
